# An In-Depth Computational Study of Alkene Cyclopropanation Catalyzed by Fe(porphyrin)(OCH_3_) Complexes. The Environmental Effects on the Energy Barriers

**DOI:** 10.1021/acs.inorgchem.0c00912

**Published:** 2020-07-27

**Authors:** Emanuele Casali, Emma Gallo, Lucio Toma

**Affiliations:** † Dipartimento di Chimica, Università di Pavia, Via Taramelli 12, 27100 Pavia, Italy; ‡ Dipartimento di Chimica, Università di Milano, Via Golgi 19, 20133 Milano, Italy

## Abstract

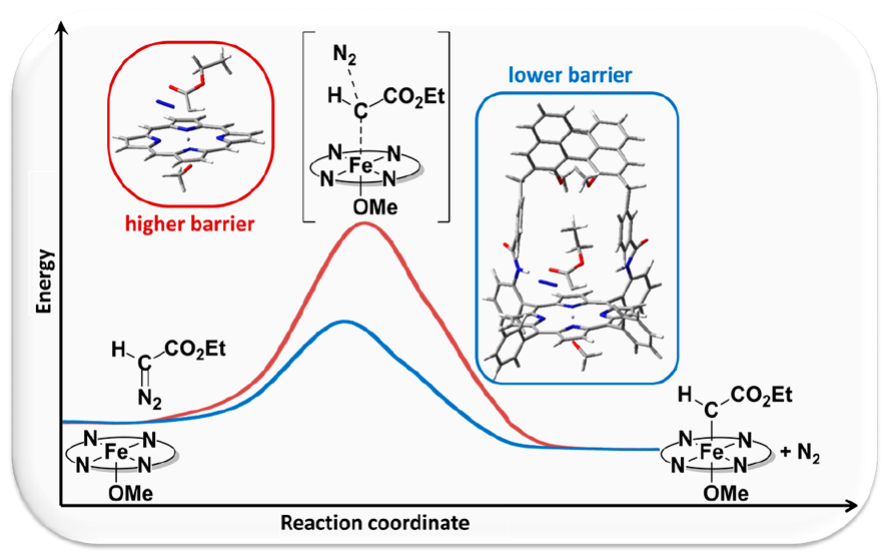

Iron porphyrin methoxy complexes, of the general formula [Fe(porphyrin)(OCH_3_)], are able to catalyze the reaction of diazo compounds with alkenes to give cyclopropane products with very high efficiency and selectivity. The overall mechanism of these reactions was thoroughly investigated with the aid of a computational approach based on density functional theory calculations. The energy profile for the processes catalyzed by the oxidized [Fe^III^(Por)(OCH_3_)] (Por = porphine) as well as the reduced [Fe^II^(Por)(OCH_3_)]^−^ forms of the iron porphyrin was determined. The main reaction step is the same in both of the cases, that is, the one leading to the *terminal*-carbene intermediate [Fe(Por)(OCH_3_)(CHCO_2_Et)] with simultaneous dinitrogen loss; however, the reduced species performs much better than the oxidized one. Contrarily to the iron(III) profile in which the carbene intermediate is directly obtained from the starting reactant complex, the favored iron(II) process is more intricate. The initially formed reactant adduct between [Fe^II^(Por)(OCH_3_)]^−^ and ethyl diazoacetate (**EDA**) is converted into a closer reactant adduct, which is in turn converted into the *terminal* iron porphyrin carbene [Fe(Por)(OCH_3_)(CHCO_2_Et)]^−^. The two corresponding transition states are almost isoenergetic, thus raising the question of whether the rate-determining step corresponds to dinitrogen loss or to the previous structural and electronic rearrangement. The ethylene addition to the *terminal* carbene is a downhill process, which, on the open-shell singlet surface, presents a defined but probably short-living diradicaloid intermediate, though other spin-state surfaces do not show this intermediate allowing a direct access to the cyclopropane product. For the crucial stationary points, the more complex catalyst [Fe(**2**)(OCH_3_)], in which a sterically hindered chiral bulk is mounted onto the porphyrin, was investigated. The corresponding computational data disclose the very significant effect of the porphyrin skeleton on the reaction energy profile. Though the geometrical features around the reactive core of the system remain unchanged, the energy barriers become much lower, thus revealing the profound effects that can be exerted by the three-dimensional organic scaffold surrounding the reaction site.

## Abstract 2

The DFT study of the Fe(porphyrin)(OCH_3_)-catalyzed cyclopropanation of alkenes by diazo compounds disclosed the profound effects that can be exerted by the ligand environment surrounding the reaction site in the formation of the intermediate *terminal* iron carbene, consisting in a significative lowering of the energy barrier for its formation.

## Introduction

Iron porphyrins are recognized as having a fundamental importance in chemistry and biology; among others, they play a key role for their function as a heme in the case of cytochromes. Enzymes of the cytochrome P450 family are able to catalyze numerous oxidative processes with very high selectivity, for example, by inserting oxygen atoms into C–H and C=C bonds through the action of an iron oxene intermediate. It is well-known that these enzymes have been engineered to become able to catalyze carbene transfer reactions through the intermediacy, in this case, of an iron carbene intermediate.^1^ Moreover, bioinspired iron porphyrin systems have been described that are able to transfer a carbene moiety in processes involving the formation of similar iron carbene intermediates,^2^ which show the carbene functionality on one of the two axial positions in the coordination sphere of iron, the other one being considered empty^3^ or, more often, occupied by neutral ligands such as imidazole derivatives^3b,4^ or anionic ligands such as chloride, methoxy, or methylthiolate.^5^ A lot of investigations have been performed to disclose the mechanism of carbene formation as well as of the subsequent carbene transfer reaction to C=C^6^ and C–H^7^ bonds with the fundamental contribution of theoretical calculations. To describe satisfactorily the electronic features of these systems, very simple computational models of the porphyrin complexes have been usually used, for example, simple porphine, thus neglecting the contribution of the overall environment in which the catalytic active metal is operating, whether it is a protein or the ligand skeleton of a bioinspired system.

**Chart 1:**
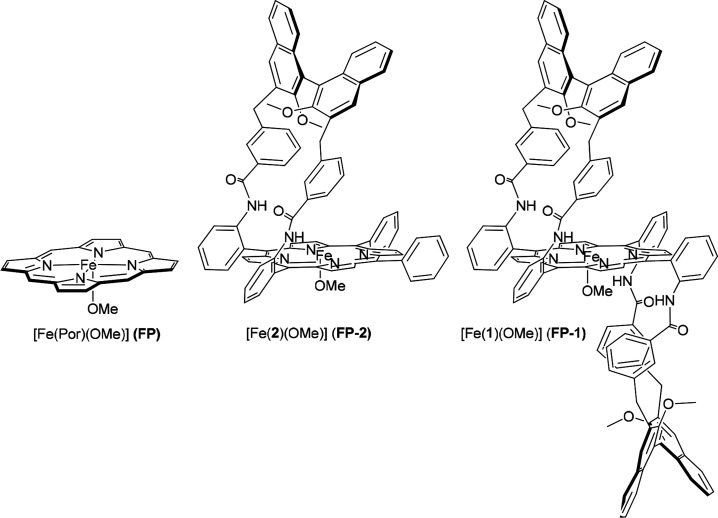
Molecular Structures of Iron Porphyrin Complexes Discussed in the Text

Among the reactions performed via iron porphyrin catalysis, cyclopropanation reactions make use of a diazo compound as the carbene source to be added to a suitable alkene. When chiral moieties are mounted onto the tetrapyrrolic core of the catalyst, stereoselective reactions can be achieved, which mimic the selectivity of the corresponding engineered metalloenzyme-catalyzed reactions.^8^ One of the most-used diazo compounds is ethyl diazoacetate (**EDA**), which, thanks to its considerable chemical stability, can be safely handled in a laboratory and, by addition to a substituted ethylene such as α-methylstyrene, can furnish cyclopropane products that sometimes show a considerable diastereo and enantioselectivity. In this context, we recently described the use of the iron(III) porphyrin methoxy complex [Fe(**1**)(OCH_3_)] (Chart 1), bearing suitable chiral *C*_2_ symmetrical moieties onto the porphyrin core, as catalysts for cyclopropanation reactions showing high turnover number (TON) and turnover frequency (TOF) values, as well as high diastereo and enantioselectivity.^9^ The stereochemical outcome of these reactions was rationalized through theoretical calculations mainly focused on the tridimensional arrangement of the ligand framework of the catalyst.^9b^ However, an in-depth investigation on the various steps of the catalytic cycle is still missing. So, we decided to study the mechanism of the reaction between **EDA** and ethylene, first using the model catalyst containing simple porphine [Fe(Por)(OCH_3_)] (**FP**) (Por = porphine), then extending the study, for the main mechanistic step, to the more complex *mono*-strapped catalyst [Fe(**2**)(OCH_3_)] (**FP-2**), in which one chiral organic moiety is mounted onto the porphyrin tetrapyrrolic core (Chart 1).

Because it has been often reported that **EDA** is able to reduce iron from the Fe(III) to the Fe(II) oxidation state,^5,10^ both electronic states should be taken into account in the mechanistic investigation. Thus, the reaction pathway involving [Fe^III^(Por)(OCH_3_)] (**FP**) and that involving the reduced methoxy porphyrin [Fe^II^(Por)(OCH_3_)]^−^ (**FP**^**–**^) complex were determined during the theoretical investigation of the reasonable reaction mechanisms.

**Scheme 1:**
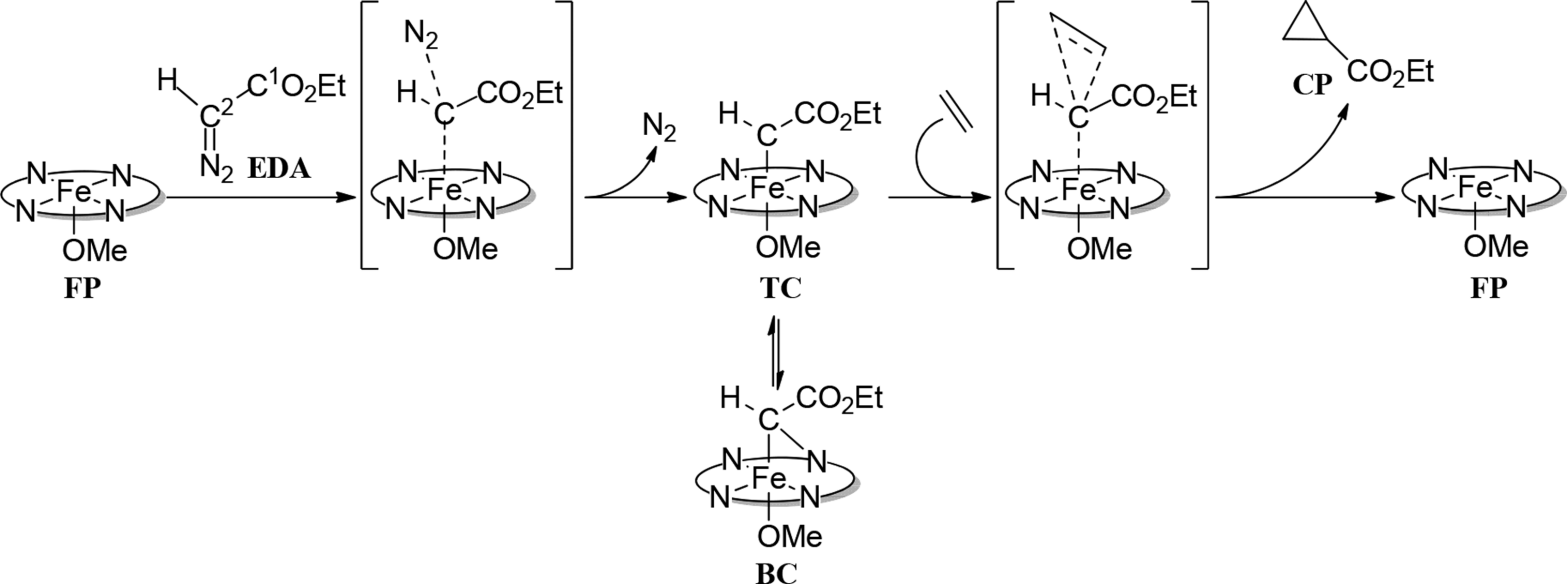
General Scheme of the Cyclopropane Formation Catalyzed by [Fe^III^(Por)(OCH_3_)] (**FP**)

Scheme 1 reports a generic overall picture of the reaction mechanism from the starting reactants to the cyclopropane product (**CP**), which shows that the initial attack of **EDA** on **FP** and the concomitant loss of dinitrogen give rise to the carbene intermediate [Fe(Por)(OCH_3_)(CHCO_2_Et)], which can exist in the two different modes, namely, *terminal*-carbene **TC** and *bridging*-carbene **BC**, though usually the former is considered to lay along the reaction pathway, whereas the latter is in equilibrium with it.^9b,11^ Reaction of ethylene with the intermediate affords the cyclopropane adduct **CP** and restores the catalyst **FP** in its initial state.

## Result and Discussion

### Reaction Catalyzed by [Fe^II^(Por)(OCH_3_)]^−^ (FP^–^)

#### Carbene Intermediates Formation

All the reactants, intermediates, and transition states along the reaction pathway were optimized in toluene using the unrestricted UB3LYP functional at the 6-31G(d) level^12^ for all the atoms, except for iron, for which the effective core potential LanL2DZ was used. With the optimized geometries, single-point energy calculations in toluene were performed using the all-electron Def2-TZVP basis set for all atoms. Dispersion corrections were computed with the Grimme D3 method. For the open-shell structures the stability of the wave function was always checked, optimizing it when found unstable. For all the species containing iron, the closed-shell singlet, open-shell singlet, triplet, and quintet spin states were investigated.

**Figure 1:**
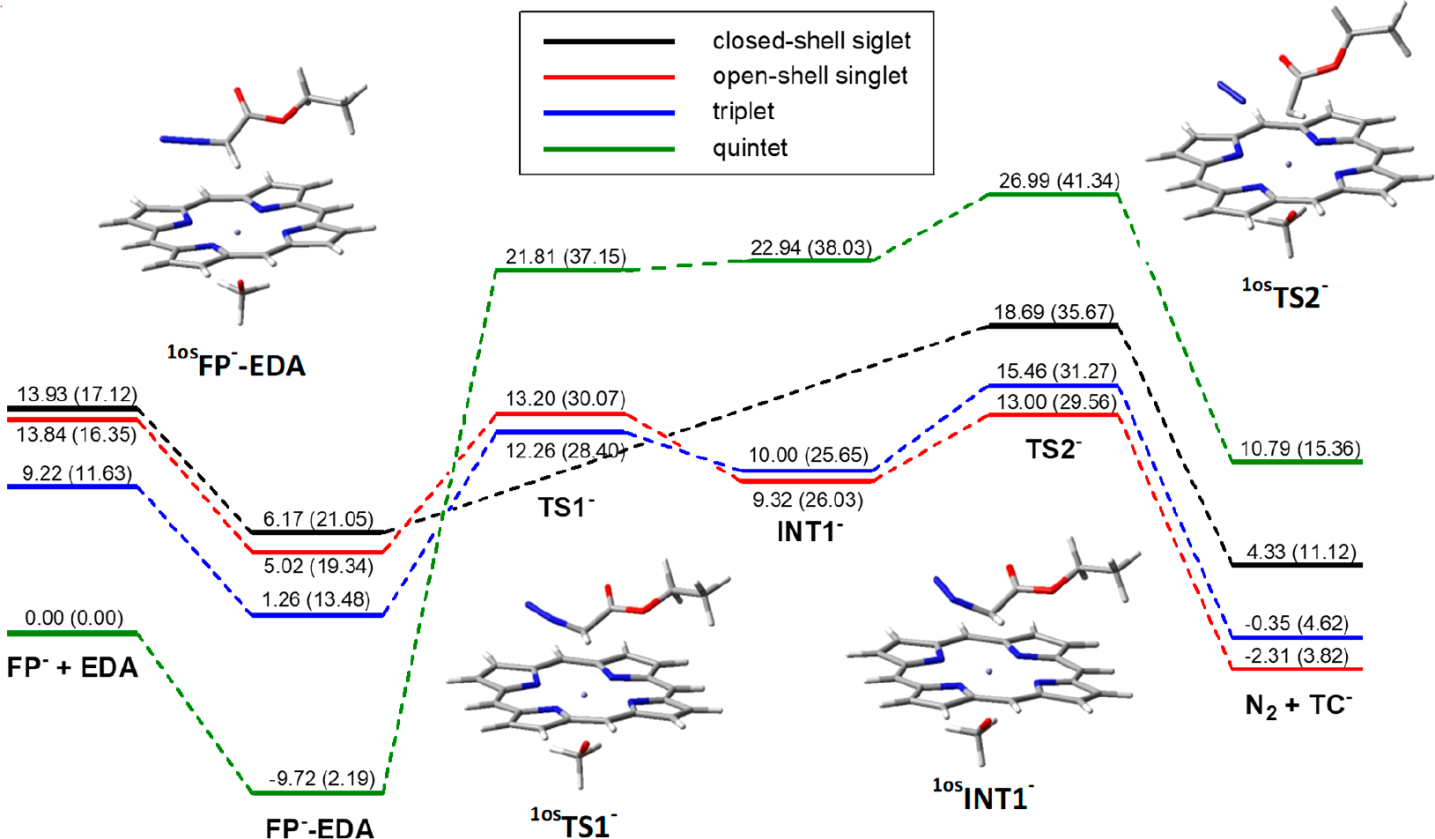
Energy profiles for the reaction of carbene intermediate formation from **EDA** and [Fe^II^(Por)(OCH_3_)]^−^, **FP**^**–**^. Energy values are from single-point Def2-TZVP calculations in toluene on geometries optimized at the UB3LYP/6-31G(d) level (LanL2DZ for iron) in toluene with zero-point correction; the corresponding Gibbs free energy is reported in parentheses. All values are dispersion-corrected.

First, **EDA** and **FP**^**–**^ were separately optimized, and the iron ground state in **FP**^**–**^ was determined to be the high-spin quintet state, ^**5**^**FP**^**–**^, preferred by 9.2 kcal/mol over the triplet state ^**3**^**FP**^**–**^ and by almost 14 kcal/mol over both the closed- and open-shell singlet states ^**1cs**^**FP**^**–**^ and ^**1os**^**FP**^**–**^. When **EDA** approaches **FP**^**–**^, an **FP**^**–**^**EDA** loose complex initially forms with a distance between iron and the **EDA** C2 atom (*d*_C2–Fe_) longer than 3.5 Å. The singlet states remained the less stable ones, and the energy gap with respect to the quintet state ^**5**^**FP**^**–**^**EDA** even increases (Figure 1 and Table S1). This ^**5**^**FP**^**–**^**EDA** local energy minimum geometry is 9.7 kcal/mol more stable than the isolated **EDA** and ^**5**^**FP**^**–**^ reactants in terms of energy, but, due to the entropy penalty, it is slightly less stable than the reactants in terms of Gibbs free energy.

The energy profile that leads to the loss of dinitrogen and the formation of the *terminal* carbene species **TC**^**–**^ was then determined, and, as shown in Figure 1, it resulted more intricate than in Scheme 1. Three of the four **FP**^**–**^**EDA** complexes, in particular, the radical or diradicaloid species, are not directly connected to the transition states corresponding to the dinitrogen loss but give rise, in the first reaction step, to three intermediate structures, ^**1os**^**INT1**^**–**^, ^**3**^**INT1**^**–**^, and ^**5**^**INT1**^**–**^, in which the distance between the **EDA** C2 and Nα (*d*_C2–Nα_) atoms is still a bond distance (∼1.45 Å). In these structures the interaction between **EDA** and iron is already significant (*d*_C2–Fe_ has shortened to a bond distance, 2.22–2.25 Å), and the stability order of the various spin states is reversed, the broken-symmetry solution of the singlet ^**1os**^**INT1**^**–**^ and the triplet ^**3**^**INT1**^**–**^ being the most stable ones and ^**5**^**INT1**^**–**^ being the least stable. A significant charge transfer is simultaneously observed, as the neutral **EDA** moiety of ^**1os**^**FP**^**–**^**EDA**, with an entire charge hosted by **FP**^**–**^, gains an overall charge of −0.490 with only −0.510 left on the iron porphyrin moiety. An inspection of the structures of the **FP**^**–**^**EDA** and **INT1**^**–**^ species showed that they differ mainly in two geometrical features, namely, the already mentioned distance between iron and the C2 carbon atom of **EDA** and the geometry of the first nitrogen atom (Nα) of **EDA**, linear in the complexes **FP**^**–**^**EDA** and trigonal planar in the intermediates **INT1**^**–**^, suggesting a change of its hybridization. We were able to locate the transition state, **TS1**^**–**^, corresponding to their interconversion characterized by a very strong negative frequency and correctly connectable, through intrinsic reaction coordinate (IRC) calculations, to **FP**^**–**^**EDA** and **INT1**^**–**^. Energy barriers of 12–13 kcal/mol with respect to isolated **EDA** and ^**5**^**FP**^**–**^ were found for ^**1os**^**TS1**^**–**^ and ^**3**^**TS1**^**–**^, while ^**5**^**TS1**^**–**^ is less stable by 10–11 kcal/mol.

Then, further shortening of *d*_C2–Fe_ leads to the three open-shell transition states ^**1os**^**TS2**^**–**^, ^**3**^**TS2**^**–**^, and ^**5**^**TS2**^**–**^. The most stable one is ^**1os**^**TS2**^**–**^, followed by the triplet and the quintet transition states. The closed-shell transition state ^**1cs**^**TS2**^**–**^, directly accessible from the reactant complex ^**1cs**^**FP**^**–**^**EDA**, is less stable than ^**3**^**TS2**^**–**^ but more stable than ^**5**^**TS2**^**–**^. The preferred transition state ^**1os**^**TS2**^**–**^ is characterized by *d*_C2–Fe_ = 2.06 Å and *d*_C2–Nα_ = 1.81 Å and by a partial charge return toward the iron porphyrin moiety (the overall charge on **EDA** is −0.351). In ^**1os**^**TS2**^**–**^ the electronic energy barrier is 13 kcal/mol with respect to isolated **EDA** and ^**5**^**FP**^**–**^ and is much higher if the Gibbs free energy is considered, 29.5 kcal/mol, a value that seems too high for a viable reaction pathway. This barrier might be overestimated due to overstabilization of the higher spin-state precursors by the B3LYP hybrid functional. However, it should be also considered that these investigations on the reaction mechanism are referred to a very simplified reaction model, whereas the real reaction is experimentally performed with [Fe(**1**)(OCH_3_)] catalyst, which shows a *C*_2_-symmetrical steric chiral bulk surrounding the tetrapyrrolic core (Chart 1). It is known that, in the enzyme-catalyzed reactions, the barrier from the reactant complex to the transition state is lowered by the enzyme environment;^6b^ the “ligand environment”, due to the large organic moiety that surrounds the reaction site, might act in a similar way. To confirm this hypothesis, transition states including the entire *bis*-strapped porphyrin **1**, instead of the simple porphine present in ^**1os**^**TS2**^**–**^, should be located, but this is beyond our current computational possibilities. In a previous paper^9b^ we showed that the behavior of the [Fe(**1**)(OCH_3_)] complex can be safely reproduced in calculations by the corresponding single-stranded porphyrin model complex [Fe(**2**)(OCH_3_)] (**FP-2**) (Chart 1). So, we tried to locate the corresponding transition state ^**1os**^**TS2–2**^**–**^, and, after a considerable computational effort, the goal was reached. While the geometrical data of **EDA** inside the reaction site found for ^**1os**^**TS2–2**^**–**^ are very similar to those of ^**1os**^**TS2**^**–**^, the electronic energy barrier is significantly lower, 5 kcal/mol for the former (Table S2) and 13 kcal/mol for the latter (Table S1). The decrease is significant also in terms of relative free energy, as the barrier approaches the value of 24 kcal/mol, 5.5 kcal/mol lower than in the simplified model, thus evidencing the large effect of the three-dimensional organic scaffold surrounding the reaction site on the energy barriers. Moreover, it should be remarked that the barrier from the isolated reactants overestimates the entropy involved. In fact, the computed free energy barrier from the reactant complex ^**5**^**FP-2**^**–**^**EDA** shows an even lower value (21.5 kcal/mol), compatible with the fast reaction catalyzed by the [Fe(**1**)(OCH_3_)] complex, able to catalyze cyclopropanation reactions even below room temperature.^9b^ It is presumably to envisage that the energy barrier of the reaction mediated by the *bis*-strapped [Fe(**1**)(OCH_3_)] complex should be approximately the same or even lower than that calculated in the presence of the *mono*-strapped [Fe(**2**)(OCH_3_)] (**FP-2**), the second strap not being directly involved in the carbene formation.

Going back to the simple porphine model, the IRC calculations performed on the four transition states **TS2**^**–**^ allowed to connect them, on the forward side, to the *terminal* carbene intermediate species [Fe(Por)(OCH_3_)(CHCO_2_Et)]^−^ (**TC**^**–**^) and dinitrogen as byproduct. The stability order of these *terminal* carbenes reflects that of the transition state leading to them, the broken-symmetry solution of the singlet ^**1os**^**TC**^**–**^ being the most stable one, with energy comparable, even lower, than that of the starting reactants (Table S1 and Figure 1). In agreement with previous experimental and computational data,^6a^ the singlet state was preferred by this carbene species.

The spin density in ^**1os**^**TC**^**–**^*terminal* carbene resides on iron and the carbon atom linked to it, evidencing an antiferromagnetic coupling between the carbon-centered radical and the unpaired electron on iron as already found in the corresponding terminal carbene bearing a methylthiolate instead of the methoxy group as the other axial ligand on iron.^6d^ The diradicaloid structure of ^**1os**^**TC**^**–**^ terminal carbene resembles that of the cobalt carbene radical species.^13^ A positive natural population analysis (NPA) charge was found on iron (*q*_Fe_ = +0.159), while the two atoms linked to it are negatively charged (*q*_C2_ = −0.119 and *q*_O_ = −0.628). The overall charge on the carbene moiety (−0.195) highlights the further charge shift toward the iron porphyrin moiety.

It is worth mentioning that the distance between iron and the methoxy oxygen atom remains almost unchanged during the reaction (from 1.901 Å in ^**5**^**FP**^**–**^ to 1.853 Å in ^**1os**^**INT1**^**–**^ and 1.905 Å in ^**1os**^**TC**^**–**^; see Table S1). The experimental evidence indicated that, regardless of the nature of the active carbene intermediate, the methoxy ligand of the catalyst is not lost during cyclopropanation.^9b^

**Figure 2:**
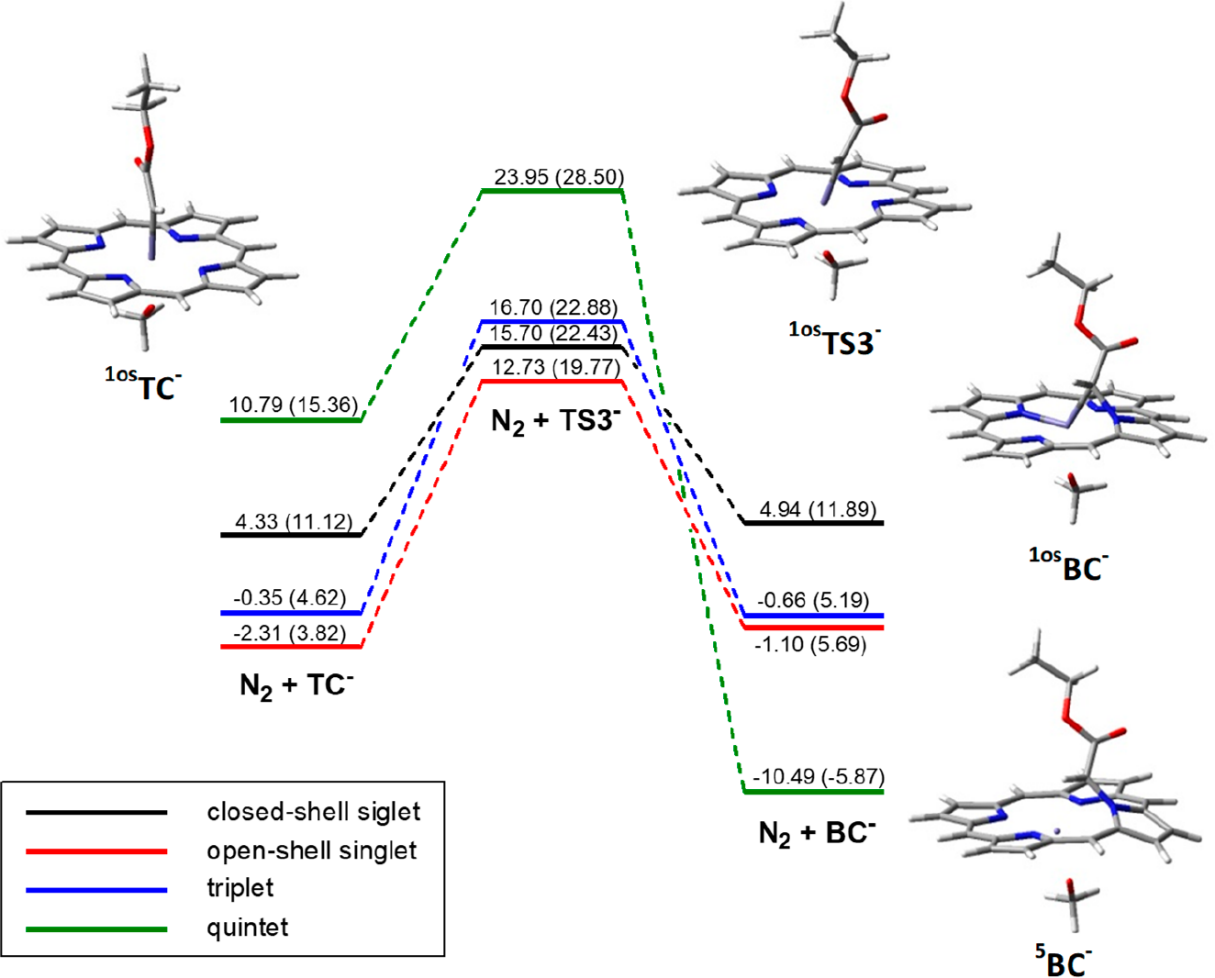
Energy profiles for the *terminal*-carbene **TC**^**–**^ and *bridging*-carbene **BC**^**–**^ interconversion. Energy values are from single-point Def2-TZVP calculations in toluene on geometries optimized at the UB3LYP/6-31G(d) level (LanL2DZ for iron) in toluene with zero-point correction; the corresponding Gibbs free energy is reported in parentheses. All values are dispersion-corrected. The energy values refer to the starting reactants ^**5**^**FP**^**–**^ and **EDA**.

Starting from the *terminal* carbenes [Fe(Por)(OCH_3_)(CHCO_2_Et)]^−^ (**TC**^**–**^) we then looked for the corresponding *bridging* structures **BC**^**–**^ and the transition states connecting them, **TS3**^**–**^. The lowest-energy transition state, ^**1os**^**TS3**^**–**^, occurs on the singlet open-shell surface with a barrier of 15 kcal/mol with respect to ^**1os**^**TC**^**–**^ and gives a *bridging* structure ^**1os**^**BC**^**–**^ almost isoenergetic to ^**1os**^**TC**^**–**^ (Table S1 and Figure 2). However, the most stable *bridging* carbene is the high-spin quintet ^**5**^**BC**^**–**^, 9.4 kcal/mol more stable than ^**1os**^**BC**^**–**^.

In the above results the broken-symmetry solutions of the singlet species could have been corrected for spin contamination using the Yamaguchi corrections of energy. If used, the corrections generally further stabilize this solution (Table S7), for example, making also **^1os^TS1**^**–**^ more stable than ^**3**^**TS1**^**–**^, but do not significantly modify the energy profiles. So, they were not added to the energy data in the figures and tables.

#### Reaction of the Carbene Intermediates with Ethylene

**Figure 3:**
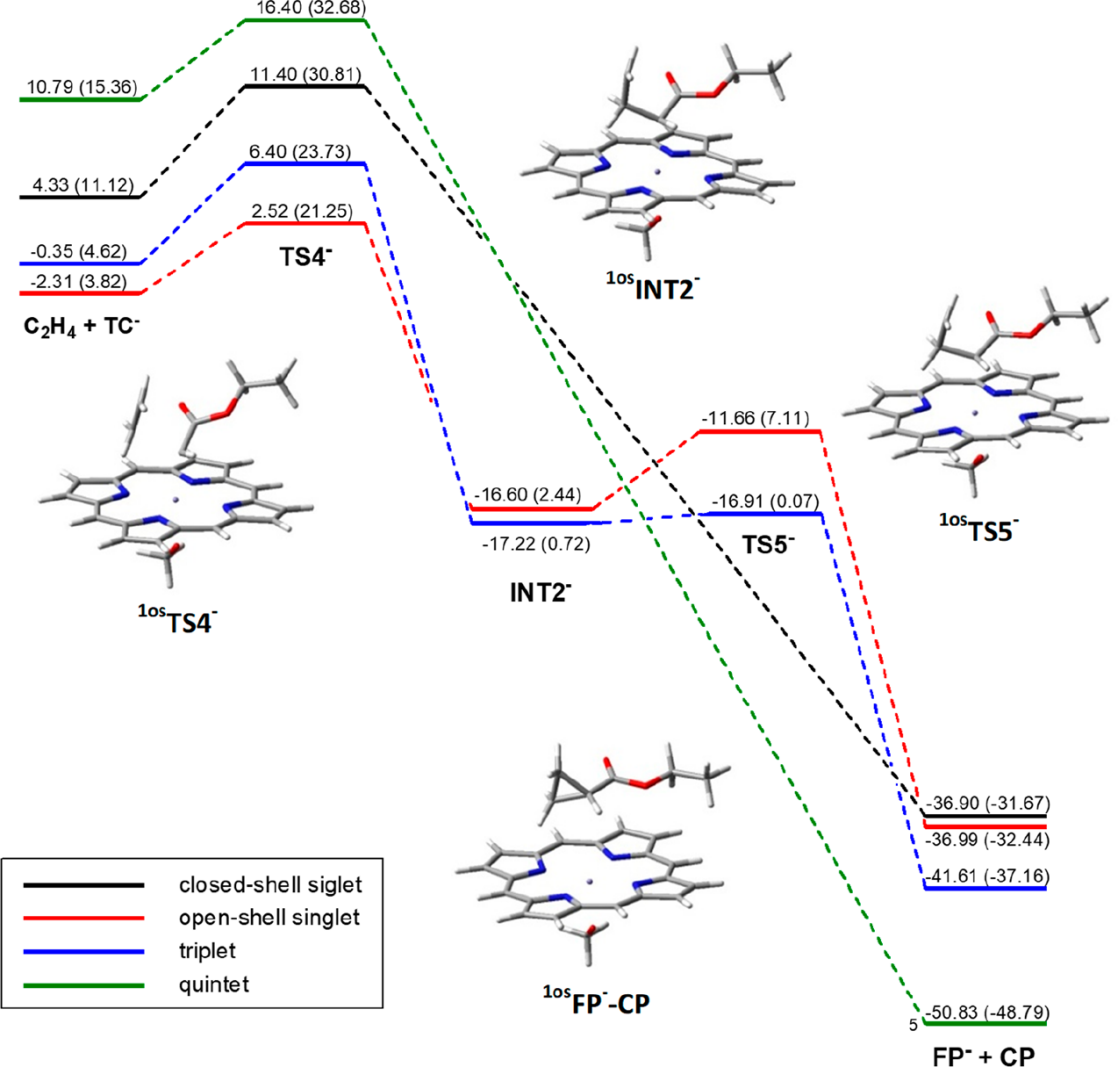
Energy profiles for the reaction of the *terminal*-carbene [Fe(Por)(OCH_3_)(CHCO_2_Et)]^−^ (**TC**^**–**^) with ethylene. The energy values are from single-point Def2-TZVP calculations in toluene on geometries optimized at the UB3LYP/6-31G(d) level (LanL2DZ for iron) in toluene with zero-point correction; the corresponding Gibbs free energy is reported in parentheses. All values are dispersion-corrected. The energy values refer to the starting reactants ^**5**^**FP**^**–**^ and **EDA**.

Then focus was placed on the right side of the cyclopropanation reaction (Scheme 1) by looking for the transition states deriving from the attack of ethylene to the porphyrin carbene intermediates [Fe(Por)(OCH_3_)(CHCO_2_Et)]^−^ (**TC**^**–**^). The most stable TS was found to be ^**1os**^**TS4**^**–**^ (Table S3 and Figure 3), which lies on the open-shell singlet surface and is characterized by a very low energy barrier (4.8 kcal/mol from ^**1os**^**TC**^**–**^), much smaller than that of the corresponding *terminal*-*bridging* interconversion.

Once again, ^**1os**^**TS4**^**–**^ is preferred over **^3^TS4**^**–**^ and largely preferred over ^**1cs**^**TS4**^**–**^ and **^5^TS4**^**–**^. The IRC path from ^**1os**^**TS4**^**–**^ leads in the forward direction to an intermediate with a diradicaloid character, ^**1os**^**INT2**^**–**^. In this intermediate one new C–C bond is already formed (1.55 Å), and the other one is far from being formed (2.52 Å), suggesting that it is a reaction intermediate with a radical nature. However, the IRC path from ^**3**^**TS4**^**–**^ gives access to a structure, ^**3**^**INT2**^**–**^, separated by the final products by a very low energy barrier, which disappears in terms of free energy. Moreover, both the closed-shell ^**1cs**^**TS4**^**–**^ and the highest-spin ^**5**^**TS4**^**–**^ transition states are directly connected to the final products. Thus, it cannot be excluded that the pathways cross after the ^**1os**^**TS4**^**–**^ transition state to generate the cyclopropane product without passing the ^**1os**^**INT2**^**–**^ intermediate. Anyway, the process ends in a deep valley, ∼50 kcal/mol below the starting reactants, both in terms of electronic energy and free energy (Table S3) with formation of cyclopropane **CP** plus the catalyst, which, after crossing to the most stable quintet ground state, is ready for a new reaction cycle.

### Reaction Catalyzed by [Fe^III^(Por)(OCH_3_)] (FP)

#### Carbene Intermediates Formation

**Figure 4:**
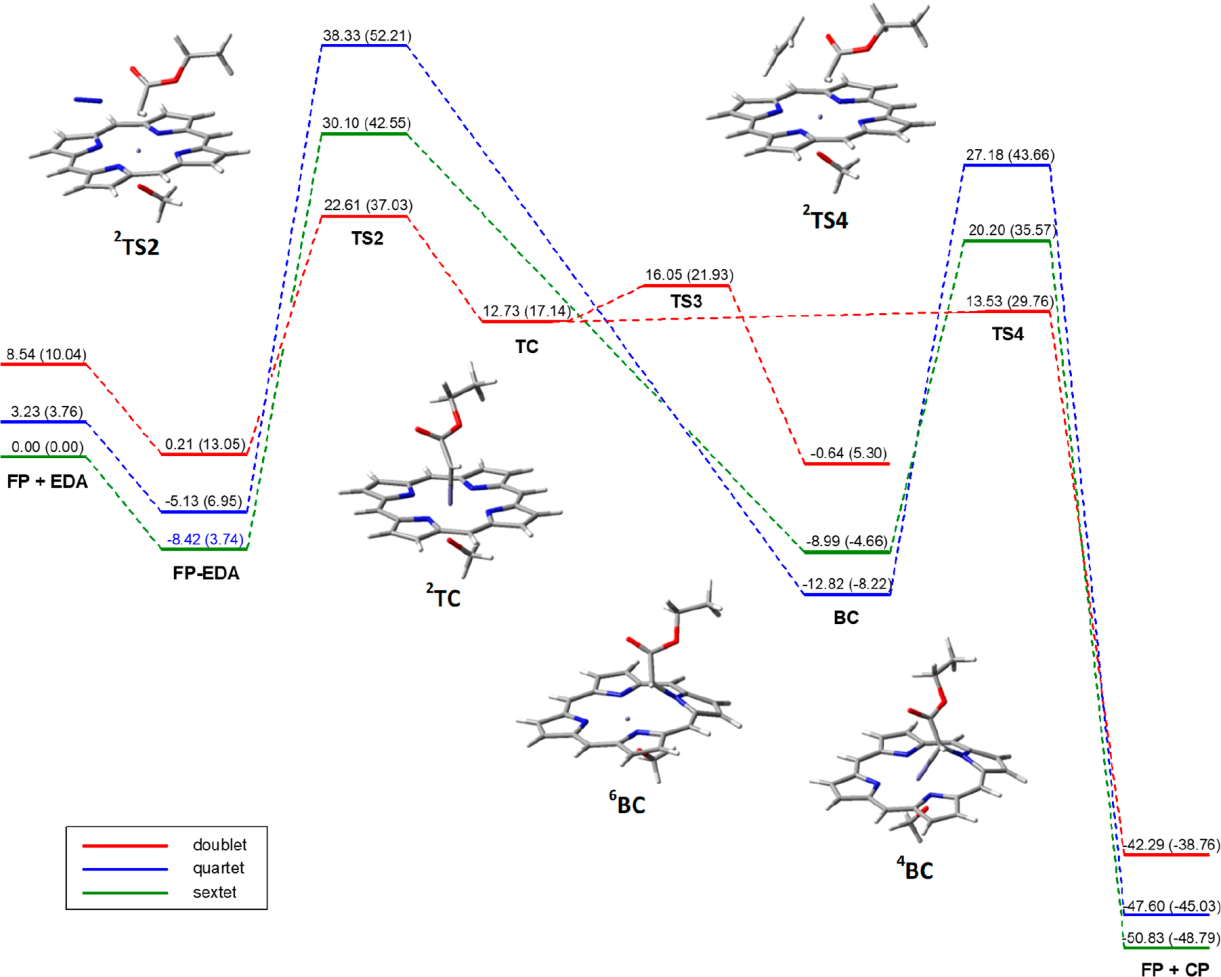
Energy profiles for the reaction of carbene intermediate formation from ethyl diazoacetate **EDA** and [Fe^III^(Por)(OCH_3_)] (**FP**) and the subsequent reaction with ethylene. The energy values are from single-point Def2-TZVP calculations in toluene on geometries optimized at the UB3LYP/6-31G(d) level (LanL2DZ for iron) in toluene with zero-point correction; the corresponding Gibbs free energy is reported in parentheses. All values are dispersion-corrected. The energy values refer to the starting reactants ^**6**^**FP** and **EDA**.

The computational approach was the same as above-described. In this case, the doublet, quartet, and sextet spin states were investigated for all the species containing iron. When the oxidized iron porphyrin [Fe^III^(Por)(OCH_3_)] (**FP**) was optimized, the preferred ground state was found to be the high-spin sextet state, ^**6**^**FP**, preferred by 3.2 and 8.5 kcal/mol over the quartet and doublet states ^**4**^**FP** and ^**2**^**FP**, respectively (Figure 4 and Table S4). The most stable reactant complex ^**6**^**FP-EDA** is 8.4 kcal/mol more stable than the isolated **EDA** and ^**6**^**FP** in terms of energy but less stable in terms of Gibbs free energy, as observed for [Fe^II^(Por)(OCH_3_)]^−^ (**FP**^**–**^). Moving from the reactant complexes at decreasing *d*_C2–Fe_ distances, once more the lowest spin state becomes preferred. In this case, no intermediate structure was observed, and the transition state for dinitrogen loss, ^**2**^**TS2**, is directly reached. The electronic energy barrier with respect to isolated **EDA** and ^**6**^**FP** is higher than with the reduced catalyst (22.6 kcal/mol) and becomes extremely high if the Gibbs free energy is considered (37 kcal/mol). The free energy barrier computed from the reactant complex ^**6**^**FP-EDA** shows a lower but still high value (33.3 kcal/mol). With the model single-stranded porphyrin complex [Fe^III^(**2**)(OCH_3_)] (**FP-2**) a significant decrease of ∼6 kcal/mol of the electronic energy barrier was observed (Table S5). The decrease is significant also in terms of the relative free energy (∼4 kcal/mol), and the barrier approaches the value of 30 kcal/mol as the computed free energy barrier from the reactant complex ^**6**^**FP-2-EDA**. Though this barrier represents a significant improvement with respect to the initial value of 37 kcal/mol, it remains much higher than in the case of the reaction catalyzed by [Fe^II^(Por)(OCH_3_)]^−^ (**FP**^**–**^).

An IRC analysis from ^**2**^**TS2** allowed connecting it, in the forward direction, to the *terminal* carbene ^**2**^**TC** and dinitrogen. The transition states for the N_2_ loss on the quartet and sextet surfaces ^**4**^**TS2** and ^**6**^**TS2** were found to be much less stable than ^**2**^**TS2** (Figure 4 and Table S4).

Contrarily to ^**2**^**TS2**, the IRC calculations from these higher spin transition states gave direct access to the *bridging* carbenes ^**4**^**BC** and ^**6**^**BC**, the former being the most stable carbene species, more than 25 kcal/mol more stable than ^**2**^**TC**. The carbene species ^**2**^**BC**, not directly obtained through the IRC calculations, was also located and optimized as well as the transitions state for the *terminal*-*bridging* interconversion, ^**2**^**TS3**, which is very *terminal*-like in its geometry and shows a very low energy barrier from the *terminal* carbene ^**2**^**TC** (3.3 kcal/mol).

#### Reaction of the Carbene Intermediates with Ethylene

The most stable transition state was found to be ^**2**^**TS4** (Table S6 and Figure 4), characterized by a very small energy barrier (0.8 kcal/mol) from the ^**2**^**TC** carbene intermediate, even lower than that of the *terminal*-*bridging* interconversion. Once again, ^**2**^**TS4** is largely preferred over ^**4**^**TS4** and ^**6**^**TS4**. The ^**2**^**TS4** transition state is concerted, though asynchronous, and the IRC path from it is connected, on the reverse side, to the terminal carbene ^**2**^**TC** and, on the forward side, directly to the cyclopropane product **CP** and the catalyst, which, after a spin crossing, regains the most stable sextet state.

## Conclusion

In this paper the overall mechanism of cyclopropanation reaction catalyzed by an iron porphyrin methoxy complex was investigated with the aid of a computational approach. In the catalyst experimentally used to perform such reactions, [Fe^III^(**1**)(OCH_3_)], iron is in the +3 oxidation state and is recovered as such at the end of the reaction to be used again with virtually unmodified catalytic performances.^9b^ However, a significant amount of literature on comparable systems suggests consideration of its reduced form, [Fe^II^(**1**)(OCH_3_)]^−^, as the catalytically active form, obtained from the resting iron(III) species by action of ethyl diazoacetate, which can promote its in situ reduction.^5,10^ During the recovery, the reduced iron(II) species is oxidized again by atmospheric oxygen to iron(III), as normally occurs during the synthesis of [Fe^III^(**1**)(OCH_3_)], performed by using FeBr_2_ as the iron source.^9a^ Thus, both the profiles for the reactions catalyzed by the oxidized as well the reduced iron species were established and compared; all the transition states and intermediates along the reaction pathways were located using the model catalyst [Fe(Por)(OCH_3_)] containing simple porphine as the iron ligand. Moreover, for the crucial stationary points, the effect on these profiles of the three-dimensional arrangement of the porphyrin skeleton, that is, the organic scaffold surrounding the reaction site, was determined by using the single-stranded methoxy iron porphyrin complex [Fe(**2**)(OCH_3_)].

As can be seen from the data illustrated in the previous section, both the reduced iron(II) and oxidized iron(III) form of the resting catalyst prefer high-spin states, the quintet and sextet states, respectively. Conversely, all the transition states encountered along the reaction coordinate, as well as all the intermediates, show low-spin preferred states, in particular, the broken-symmetry solution of the singlet for iron(II). However, in this solution spin density might be merely a reflection of the triplet spin contamination, and the real singlet species may have an electronic structure between the closed-shell and the broken-symmetry singlet solutions found for this species. Though the rate-determining step is the same in the both cases, that is, the one leading to the *terminal*-carbene intermediate with simultaneous dinitrogen loss, [Fe^II^(Por)(OCH_3_)]^−^ (**FP**^**–**^) performs much better than [Fe^III^(Por)(OCH_3_)] (**FP**) due to a much smaller energy barrier (29.5 with respect to 37 kcal/mol in terms of the Gibbs free energy). Contrarily to the iron(III) profile in which the carbene intermediate is directly obtained from the starting complex, the favored iron(II) process is more intricate, as already found in the case of [Fe^II^(Por)(SCH_3_)]^−^ ^6^^d^ or [Fe^II^(Por)(Cl)]^−^.^14^ The initially formed reactant adduct **FP**^**–**^**EDA** between the starting catalyst [Fe^II^(Por)(OCH_3_)]^−^ (**FP**^**–**^) and ethyl diazoacetate is converted into a closer adduct **INT1**^**–**^, which is in turn converted into the *terminal* iron porphyrin carbene intermediate [Fe(Por)(OCH_3_)(CHCO_2_Et)]^−^ (**TC**^**–**^), passing through the main transition state. We located also the transition state between **FP**^**–**^**EDA** and **INT1**^**–**^ and found that it is almost isoenergetic with the main transition state, thus raising the question whether the rate-determining step corresponds to dinitrogen loss or to an electronic and structural rearrangement in ethyl diazoacetate made evident by the change from the linear to the trigonal planar geometry of its first nitrogen atom and by a significant shortening of the Fe–C2 distance. Actually, the barrier of the two steps has comparable heights so that, whatever the highest barrier, the reaction rate is almost unaffected.

The ethylene addition to the *terminal* carbene is a downhill process, which, on the broken-symmetry solution of the singlet surface, presents a defined but elusive intermediate. This intermediate is badly defined on the triplet surface and does not exist on the closed-shell singlet and quintet surfaces.

Finally, the computational data obtained with the single-stranded porphyrin catalyst [Fe^II^(**2**)(OCH_3_)]^−^ made clear the very significant effects of the three-dimensional scaffold surrounding the reaction site on the reaction profile and underline the strong influence of the entire catalyst on its performance. Though the geometrical features around the reactive core of the system remain unchanged, the energy barrier becomes much lower (a Gibbs free energy value of 21.5 kcal/mol with respect to the reactant complex), making feasible an apparently unfeasible reaction.

This paper further advances the understanding of the mechanism of action of the metal porphyrin complexes in the particular case of the iron porphyrin methoxy complexes. It compares the predictable energy profiles determined for both the oxidized and reduced forms of the catalyst on all the reasonable spin states of the metal center and describes all the mechanistic detail of the carbene intermediate formation. Further studies will determine the extensibility of these results to other iron porphyrin complexes.

## Supporting Information Available

Computational details, electronic energy, and Cartesian coordinates of all computed structures (PDF)
