# Supplementary material for: An In-Depth Computational Study of Alkene Cyclopropanation Catalyzed by Fe(porphyrin)(OCH3) Complexes. The Environmental Effects on the Energy Barriers
Source: Inorg Chem. 2020 Jul 27;59(16):11329–36. doi: 10.1021/acs.inorgchem.0c00912 (PMC8009515; doi:10.1021/acs.inorgchem.0c00912)
Supplement: Supplementary file 1 — ic0c00912_si_001.pdf [file ic0c00912_si_001.pdf]

Supporting information

An In-depth Computational Study of Alkene Cyclopropanation Catalyzed by Fe(porphyrin)(OCH_3) Complexes. The Environmental Effects on the Energy Barriers

Emanuele Casali,† Emma Gallo, ‡ Lucio Toma†*

† *Dipartimento di Chimica, Università di Pavia, Via Taramelli 12, 27100 Pavia, Italy*

e-mail: lucio.toma@unipv.it

‡ *Dipartimento di Chimica, Università di Milano, Via Golgi 19, 20133 Milano, Italy*

Computational details page S2

Table S1 page S3

Table S2 page S4

Table S3 page S5

Table S4 page S6

Table S5 page S6

Table S6 page S7

Table S7 page S8

Cartesian coordinates page S10

Computational details

All structures were optimized with the Gaussian 09 program package,[1] using the UB3LYP functional at the 6-31G(d) level for all the atoms, but for iron, for which the effective core potential LanL2DZ was used. All the optimization were performed in toluene solvent, using the SMD solvation model. With the optimized geometries, single-point energy calculations in toluene were performed using the all-electron Def2-TZVP basis set for all atoms. Dispersion corrections were computed with the Grimme's D3 method. After optimizations, frequency analyses were performed at the UB3LYP/6-31G(d) level (LanL2DZ for iron) to define the optimized structures as minima or transition states. The zero-point energy so determined was added to the Def2-TZVP single-point energy to give the electronic energy as reported throughout the manuscript whereas addition of the free energy correction to the single-point energy gives the free energy values. Intrinsic reaction coordinate (IRC) calculations were performed to connect the transition states with the corresponding reactants and products. For the open-shell structures the stability of the wavefunction was always checked, optimizing it when found unstable. For all the species containing iron, the various spin states were investigated.

1. Frisch, M. J.; Trucks, G. W.; Schlegel, H. B.; Scuseria, G. E.; Robb, M. A.; Cheeseman, J. R.; Scalmani, G.; Barone, V.; Mennucci, B.; Petersson, G. A.; Nakatsuji, H.; Caricato, M.; Li, X.; Hratchian, H. P.; Izmaylov, A. F.; Bloino, J.; Zheng, G.; Sonnenberg, J. L.; Hada, M.; Ehara, M.; Toyota, K.; Fukuda, R.; Hasegawa, J.; Ishida, M.; Nakajima, T.; Honda, Y.; Kitao, O.; Nakai, H.; Vreven, T.; Montgomery, J. A., Jr.; Peralta, J. E.; Ogliaro, F.; Bearpark, M.; Heyd, J. J.; Brothers, E.; Kudin, K. N.; Staroverov, V. N.; Keith, T.; Kobayashi, R.; Normand, J.; Raghavachari, K.; Rendell, A.; Burant, J. C.; Iyengar, S. S.; Tomasi, J.; Cossi, M.; Rega, N.; Millam, J. M.; Klene, M.; Knox, J. E.; Cross, J. B.; Bakken, V.; Adamo, C.; Jaramillo, J.; Gomperts, R.; Stratmann, R. E.; Yazyev, O.; Austin, A. J.; Cammi, R.; Pomelli, C.; Ochterski, J. W.; Martin, R. L.; Morokuma, K.; Zakrzewski, V. G.; Voth, G. A.; Salvador, P.; Dannenberg, J. J.; Dapprich, S.; Daniels, A. D.; Farkas, O.; Foresman, J. B.; Ortiz, J. V.; Cioslowski, J.; Fox, D. J. Gaussian 09, Revision B.01; Gaussian, Inc., Wallingford, CT, 2010.

Table S1. Relative electronic energy, with zero-point correction, and the corresponding Gibbs free energy (kcal/mol) of the transition states and intermediates in the reaction of carbene intermediate formation from ethyl diazoacetate (**EDA**) and $[Fe^{II}(Por)(OCH_3)]^-$ (**FP$^-$**) determined through single-point Def2-TZVP calculations in toluene on geometries optimized at the UB3LYP/6-31G(d) level (LanL2DZ for iron) in toluene. The distances (Å) between **EDA** C2 atom and leaving N_2 ($d_{C2\text{-}N\alpha}$), iron ($d_{C2\text{-}Fe}$), and the closest porphyrin nitrogen atom ($d_{C2\text{-}N}$) are reported for each stationary point.

	E_{rel}	G_{rel}	$d_{C2\text{-}N\alpha}$	$d_{C2\text{-}Fe}$	$d_{C2\text{-}N}$	$d_{Fe\text{-}O}$
**^{1cs}FP$^-$ + EDA**	13.91	17.12				1.898
**^{1os}FP$^-$ + EDA**	13.82	16.35				1.935
**^{3}FP$^-$ + EDA**	9.20	11.63				2.008
**^{5}FP$^-$ + EDA**	0.00	0.00				1.901
**^{1cs}FP$^-$-EDA**	6.15	21.05	1.307	3.468	3.855	1.900
**^{1os}FP$^-$-EDA**	5.00	19.34	1.306	3.575	3.887	1.937
**^{3}FP$^-$-EDA**	1.24	13.48	1.305	3.772	3.805	1.977
**^{5}FP$^-$-EDA**	-9.74	2.19	1.306	3.929	3.592	1.895
**^{1os}TS1$^-$**	13.18	30.07	1.367	2.460	3.048	1.853
**^{3}TS1$^-$**	12.24	28.40	1.345	2.768	3.160	1.798
**^{5}TS1$^-$**	21.79	37.15	1.439	2.283	2.935	1.892
**^{1os}INT1$^-$**	9.30	26.03	1.452	2.231	2.899	1.853
**^{3}INT1$^-$**	9.98	25.65	1.447	2.251	2.912	1.849
**^{5}INT1$^-$**	22.91	38.03	1.455	2.223	2.921	1.868
**^{1cs}TS2$^-$**	18.67	35.67	1.740	1.987	2.687	1.934
**^{1os}TS2$^-$**	12.98	29.56	1.815	2.064	2.794	1.878
**^{3}TS2$^-$**	15.44	31.27	1.857	2.104	2.801	1.863
**^{5}TS2$^-$**	26.97	41.34	1.844	2.081	2.827	1.872
**N2 + ^{1cs}TC$^-$**	4.31	11.12		1.798	2.698	1.947
**N2 + ^{1os}TC$^-$**	-2.33	3.82		1.939	2.734	1.904
**N2 + ^{3}TC$^-$**	-0.37	4.62		1.971	2.752	1.891
**N2 + ^{5}TC$^-$**	10.77	15.36		1.959	2.767	1.884
**N2 + ^{1cs}TS3$^-$**	15.68	22.43		1.874	1.985	1.942
**N2 + ^{1os}TS3$^-$**	12.71	19.77		1.925	1.980	1.902

**N2 + ^{3}TS3^{-}**	16.67	22.88		1.994	2.004	1.875
**N2 + ^{5}TS3^{-}**	23.93	28.50		2.081	2.136	1.876
**N2 + ^{1cs}BC^{-}**	4.94	11.89		2.058	1.452	1.896
**N2 + ^{1os}BC^{-}**	-1.12	5.69		2.005	1.472	1.866
**N2 + ^{3}BC^{-}**	-0.68	5.19		2.011	1.467	1.865
**N2 + ^{5}BC^{-}**	-10.51	-5.87		2.491	1.418	1.898

Table S2. Relative electronic energy, with zero-point correction, and the corresponding Gibbs free energy (kcal/mol) of the transition states in the reaction of *terminal*-carbene intermediate formation from ethyl diazoacetate **EDA** and [Fe^{II}(**2**)(OCH$_3$)]$^-$, **FP-2$^-$**, determined through single-point Def2-TZVP calculations in toluene on geometries optimized at the UB3LYP/6-31G(d) level (LanL2DZ for iron) in toluene. The distances (Å) between **EDA** C2 atom and leaving N$_2$ ($d_{C2\text{-}N\alpha}$), iron ($d_{C2\text{-}Fe}$), and the closest porphyrin nitrogen atom ($d_{C2\text{-}N}$) are reported for each stationary point.

	E_{rel}	G_{rel}	$d_{C2\text{-}N\alpha}$	$d_{C2\text{-}Fe}$	$d_{C2\text{-}N}$	$d_{Fe\text{-}O}$
**^{5}FP-2^{-} + EDA**	0.00	0.00				1.896
**^{5}FP-2^{-}-EDA**	-10.09	2.45	1.304	6.594	4.836	1.896
**^{1os}INT1-2^{-}**	2.20	21.44	1.453	2.224	2.949	1.850
**^{1os}TS2-2^{-}**	4.98	23.96	1.818	2.055	2.809	1.874

Table S3. Relative electronic energy, with zero-point correction, and the corresponding Gibbs free energy (kcal/mol) of the transition states and intermediates in the reaction of ethylene with the *terminal*-carbene $[Fe^{II}(Por)(OCH_3)(CHCO_2Et)]^-$ intermediates $\mathbf{TC^-}$ determined through single-point Def2-TZVP calculations in toluene on geometries optimized at the UB3LYP/6-31G(d) level (LanL2DZ for iron) in toluene. The energy values are referred to the starting reactants $\mathbf{^5FP^-}$ and **EDA**. The distances (Å) between the carbene C2 atom and the incoming ethylene carbon atoms ($d_{C2\text{-}CE1}$ and $d_{C2\text{-}CE2}$), iron ($d_{C2\text{-}Fe}$), and the closest porphyrin nitrogen atom ($d_{C2\text{-}N}$) are reported for each stationary point.

	E_{rel}	G_{rel}	$d_{C2\text{-}CE1}$	$d_{C2\text{-}CE2}$	$d_{C2\text{-}Fe}$	$d_{C2\text{-}N}$	$d_{Fe\text{-}O}$
$\mathbf{^{1cs}TS4^-}$	11.38	30.81	2.129	2.656	1.942	2.627	1.938
$\mathbf{^{1os}TS4^-}$	2.50	21.25	2.221	2.954	2.022	2.710	1.898
$\mathbf{^{3}TS4^-}$	6.38	23.73	2.269	3.033	2.078	2.761	1.881
$\mathbf{^{5}TS4^-}$	16.38	32.68	2.270	2.995	2.043	2.793	1.890
$\mathbf{^{1os}INT2^-}$	-16.62	2.44	1.553	2.520	2.190	2.898	1.887
$\mathbf{^{3}INT2^-}$	-17.24	0.72	1.549	2.499	2.204	2.898	1.890
$\mathbf{^{1os}TS5^-}$	-11.68	7.11	1.524	2.191	2.461	3.023	1.901
$\mathbf{^{3}TS5^-}$	-16.93	0.07	1.512	2.203	2.551	3.070	1.951
$\mathbf{CP + {}^{1cs}FP^-}$	-36.92	-31.67					
$\mathbf{CP + {}^{1os}FP^-}$	-37.01	-32.44					
$\mathbf{CP + {}^{3}FP^-}$	-41.63	-37.16					
$\mathbf{CP + {}^{5}FP^-}$	-50.83	-48.79					

Table S4. Relative electronic energy, with zero-point correction, and the corresponding Gibbs free energy (kcal/mol) of the transition states and intermediates in the reaction of carbene intermediate formation from ethyl diazoacetate **EDA** and [Fe^{III}(Por)(OCH_3)] (**FP**), determined through single-point Def2-TZVP calculations in toluene on geometries optimized at the UB3LYP/6-31G(d) level (LanL2DZ for iron) in toluene. The distances (Å) between **EDA** C2 atom and leaving N_2 ($d_{C2\text{-}N\alpha}$), iron ($d_{C2\text{-}Fe}$), and the closest porphyrin nitrogen atom ($d_{C2\text{-}N}$) are reported for each stationary point.

	E_{rel}	G_{rel}	$d_{C2\text{-}N\alpha}$	$d_{C2\text{-}Fe}$	$d_{C2\text{-}N}$	$d_{Fe\text{-}O}$
**^{2}FP + EDA**	8.54	10.04				1.780
**^{4}FP + EDA**	3.23	3.76				1.856
**^{6}FP + EDA**	0.00	0.00				1.819
**^{2}FP-EDA**	0.21	13.05	1.307	3.644	4.151	1.780
**^{4}FP-EDA**	-5.13	6.95	1.306	3.701	4.101	1.859
**^{6}FP-EDA**	-8.42	3.74	1.306	3.865	4.026	1.819
**^{2}TS2**	22.61	37.03	1.979	1.989	2.613	1.821
**^{4}TS2**	38.33	52.21	1.971	2.008	2.576	1.815
**^{6}TS2**	30.10	42.55	1.990	2.364	2.361	1.823
**N2 + ^{2}TC**	12.73	17.14		1.852	2.672	1.833
**N2 + ^{2}TS3**	16.05	21.93		1.871	2.367	1.828
**N2 + ^{2}BC**	-0.64	5.30		1.994	1.472	1.829
**N2 + ^{4}BC**	-12.82	-8.22		2.034	1.438	1.830
**N2 + ^{6}BC**	-8.99	-4.66		2.181	1.431	1.852

Table S5. Relative electronic energy, with zero-point correction, and the corresponding Gibbs free energy (kcal/mol) of the transition states in the reaction of *terminal*-carbene intermediate formation from ethyl diazoacetate **EDA** and [Fe^{III}(**2**)(OCH_3)] **(FP-2)**, determined through single-point Def2-TZVP calculations in toluene on geometries optimized at the UB3LYP/6-31G(d) level (LanL2DZ for iron) in toluene. The distances (Å) between **EDA** C2 atom and leaving N_2 ($d_{C2\text{-}N\alpha}$), iron ($d_{C2\text{-}Fe}$), and the closest porphyrin nitrogen atom ($d_{C2\text{-}N}$) are reported for each stationary point.

	E_{rel}	G_{rel}	$d_{C2\text{-}N\alpha}$	$d_{C2\text{-}Fe}$	$d_{C2\text{-}N}$	$d_{Fe\text{-}O}$
**^{6}FP-2 + EDA**	0.00	0.00				1.819
**^{6}FP-2-EDA**	-11.99	2.60	1.306	5.656	6.831	1.820
**^{2}TS2-2**	16.88	33.18	1.966	1.978	2.665	1.822

Table S6. Relative electronic energy, with zero-point correction, and the corresponding Gibbs free energy (kcal/mol) of the transition states in the reaction of ethylene with the [Fe^{III}(Por)(OCH_3)($CHCO_2Et$)] carbene intermediates determined through single-point Def2-TZVP calculations in toluene on geometries optimized at the UB3LYP/6-31G(d) level (LanL2DZ for iron) in toluene. The energy values are referred to the starting reactants ^{6}**FP** and **EDA**. The distances (Å) between the carbene C2 atom and the incoming ethylene carbon atoms ($d_{C2\text{-}CE1}$ and $d_{C2\text{-}CE2}$), iron ($d_{C2\text{-}Fe}$), and the closest porphyrin nitrogen atom ($d_{C2\text{-}N}$) are reported for each stationary point.

	E_{rel}	G_{rel}	$d_{C2\text{-}CE1}$	$d_{C2\text{-}CE2}$	$d_{C2\text{-}Fe}$	$d_{C2\text{-}N}$	$d_{Fe\text{-}O}$
^{2}**TS4**	13.53	29.76	2.611	2.874	1.929	2.482	1.827
^{4}**TS4**	27.18	43.66	2.243	2.623	2.145	2.171	1.825
^{6}**TS4**	20.20	35.57	2.328	2.700	2.288	2.117	1.852
**CP** + ^{2}**FP**	-42.29	-38.76					
**CP** + ^{4}**FP$^-$**	-47.60	-45.03					
**CP** + ^{6}**FP**	-50.83	-48.79					

Table S7. Yamaguchi's corrections for the open-shell singlet species of iron-porphyrin species. The correction formulas[2] are reported below.

$$f_{sc} = \frac{\langle S^2\rangle_{OSS}}{\langle S^2\rangle_{triplet\ in\ OOS\ geometry} - \langle S^2\rangle_{OSS}}$$

$$E_{correction} = f_{sc} \cdot (E_{OSS} - E_{triplet\ in\ OSS\ geometry})$$

Energies are at the UB3LYP(GD3BJ)/def2tzvp/SMD(toluene) level of theory. The stability of the wave function was tested for each species.

	E (a.u.)	<S²> before annihilation	<S²> after annihilation	f_{sc}	$E_{correction}$ (a.u.)	$E_{correction}$ (kcal/mol)
**^{1os}FP$^-$**	-2367.955395	0.6281	0.0966			
**triplet in OSS geometry**	-2367.961532	2.4546	2.0219	0.344	0.00211	1.32
**^{1os}FP$^-$-EDA**	-2784.110044	0.6245	0.1011			
**triplet in OSS geometry**	-2784.115668	2.4137	2.0190	0.349	0.00196	1.23
**^{1os}TS1$^-$**	-2784.095122	0.8616	0.2251			
**triplet in OSS geometry**	-2784.091468	2.0439	2.0008	0.729	-0.00266	-1.67
**^{1os}INT1$^-$**	-2784.103969	1.0194	0.2553			
**triplet in OSS geometry**	-2784.103125	2.0265	2.0004	1.012	-0.00085	-0.54
**^{1os}TS2$^-$**	-2784.095544	0.9364	0.2101			
**triplet in OSS geometry**	-2784.090433	2.0267	2.0004	0.859	-0.00439	-2.75
**^{1os}TC$^-$**	-2674.548627	0.8397	0.1443			
**triplet in OSS geometry**	-2674.537042	2.0279	2.0004	0.707	-0.00819	-5.14
**^{1os}TS3$^-$**	-2674.525110	0.6884	0.0970			
**triplet in OSS geometry**	-2674.513662	2.0427	2.0010	0.508	-0.00582	-3.65
**^{1os}BC$^-$**	-2674.549237	0.9415	0.1489			
**triplet in OSS geometry**	-2674.547637	2.0735	2.0020	0.832	-0.00133	-0.84
**^{1os}TS4$^-$**	-2753.172532	0.9016	0.2679			
**triplet in OSS geometry**	-2753.165206	2.0435	2.0010	0.790	-0.00578	-3.63
**^{1os}INT2$^-$**	-2753.205822	1.0202	0.2023			
**triplet in OSS geometry**	-2753.206204	2.0352	2.0006	1.005	0.00038	0.24
**^{1os}TS5$^-$**	-2753.197051	0.9510	0.3135			
**triplet in OSS geometry**	-2753.200643	2.1226	2.0036	0.812	0.00292	1.83

2. Kitagawa, Y.; Saito, T.; Ito, M.; Shoji, M.; Koizumi, K.; Yamanaka, S.; Kawakami, T.; Okumura, M.; Yamaguchi, K. *Chem. Phys. Lett*. **2007**, 442, 445.

EDA b3lyp/def2tzvp, el. energy = -416.139030 a.u.

C	-1.29467600	-0.79862400	-0.00014000
C	-0.22256600	0.19182300	-0.00009600
O	-0.37873000	1.40038100	-0.00012800
O	0.97500100	-0.43261200	-0.00009800
C	2.13708400	0.43239500	0.00010200
H	2.09874000	1.07564600	0.88507900
H	2.09886600	1.07605800	-0.88454500
N	-3.58771700	0.04076400	0.00021400
N	-2.52370100	-0.35971300	0.00002300
C	3.36554300	-0.45434600	0.00008000
H	3.39122100	-1.09423100	-0.88845300
H	4.26693000	0.16904600	-0.00059900
H	3.39197600	-1.09335300	0.88922400
H	-1.15028500	-1.87016400	-0.00023600

^{1cs}FP$^-$ b3lyp/def2tzvp, el. energy = =-2367.955093 a.u.

C	1.72642300	2.48027200	-0.24833200
N	1.63992300	1.10837300	-0.22332700
H	3.44132000	3.92881300	-0.39376500
C	2.93333800	0.65167300	-0.32450600
C	3.85747100	1.76185900	-0.40438900
H	4.93379700	1.66717700	-0.49161500
C	3.10782600	2.89821300	-0.35363700
C	3.30765400	-0.68856600	-0.35520000
N	1.06759400	-1.67938300	-0.21413300
C	0.60594500	-2.97310300	-0.21196200
C	1.71025300	-3.90546400	-0.29997600
H	1.61003800	-4.98465200	-0.31814700
C	2.84729900	-3.15863000	-0.36587900
H	3.87388300	-3.49749100	-0.44493700
C	2.43604700	-1.77231400	-0.31165500
C	0.64294300	3.35288700	-0.21115600
H	0.86237100	4.41762100	-0.23176000
H	-3.97356600	3.49676400	-0.22321200
C	-2.94322900	3.16030800	-0.20701600
C	-2.52558600	1.77454500	-0.17898700
H	-1.71011200	4.98954500	-0.23601900
N	-1.15499800	1.68514100	-0.17076600
C	-0.69721400	2.97919200	-0.18982300
C	-1.80653100	3.91008000	-0.21288600
C	-0.73377100	-3.34664800	-0.17472500
H	-0.95210700	-4.41185300	-0.17558500
H	-5.03486900	-1.66379300	-0.21573800
C	-3.95513500	-1.75916800	-0.20185300
C	-3.20446000	-2.89544900	-0.19391800
H	-3.53987100	-3.92619500	-0.20186800
C	-1.81903800	-2.47539900	-0.17624600
N	-1.73094200	-1.10637600	-0.17617100
C	-3.02528100	-0.64921700	-0.18510800
C	-3.39879700	0.69116200	-0.17817700
H	-4.46367000	0.91112600	-0.18447600
H	4.36998700	-0.90663400	-0.43363900
Fe	-0.04056300	-0.00123100	-0.02272700
O	-0.16310800	-0.05493300	1.87074700
C	0.97364300	0.01429200	2.64210900
H	0.71278000	-0.17092200	3.70670100
H	1.75248200	-0.73846600	2.39110800
H	1.48720400	1.00299500	2.62860100

^{1os}FP$^-$ ub3lyp/def2tzvp, el. energy = -2367.955395 a.u.

C	1.47492000	2.62751500	-0.26877700
N	1.52328000	1.25438800	-0.23749400
H	3.04427200	4.23444200	-0.40924700
C	2.85425100	0.92325400	-0.32926100
C	3.66757200	2.11858400	-0.40632900
H	4.74849800	2.12810400	-0.48609200
C	2.81133000	3.17664900	-0.36720500
C	3.35896500	-0.37358400	-0.35253400
N	1.22865100	-1.58265200	-0.23691400
C	0.89154600	-2.91214100	-0.23147300
C	2.08071100	-3.73318400	-0.30656800
H	2.08609000	-4.81700900	-0.32040000
C	3.14242800	-2.88014800	-0.36159700
H	4.19739200	-3.12005800	-0.42800400
C	2.59831600	-1.54019600	-0.31681400
C	0.31264400	3.39328900	-0.23636900
H	0.43064600	4.47400900	-0.25615800
H	-4.30014800	3.09849500	-0.22202100
C	-3.24252000	2.86108600	-0.21645000
C	-2.69342600	1.52184600	-0.19613200
H	-2.19015200	4.80032500	-0.24370100
N	-1.32293300	1.56725400	-0.19845900
C	-0.98876100	2.89676600	-0.21523300
C	-2.18175400	3.71654900	-0.22719800
C	-0.40905300	-3.40913200	-0.18361800
H	-0.52596700	-4.49014600	-0.18492600
H	-4.85258400	-2.14738400	-0.19118900
C	-3.76880600	-2.13793200	-0.17994800
C	-2.91206200	-3.19573300	-0.17502500
H	-3.14678200	-4.25396600	-0.18218800
C	-1.57210000	-2.64435000	-0.16550500
N	-1.61884200	-1.27289900	-0.16080300
C	-2.95073300	-0.94196200	-0.17084200
C	-3.45481300	0.35535200	-0.17911900
H	-4.53630400	0.46745200	-0.18055200
H	4.43846100	-0.48499800	-0.41863300
Fe	-0.04204200	-0.00917900	-0.01479500
O	-0.12578200	-0.05178200	1.91815200
C	0.98757500	0.20411300	2.68025900
H	0.76849500	0.03303700	3.75863500
H	1.86533400	-0.44430000	2.45396500
H	1.36363800	1.25243000	2.62006600

^{3}FP$^-$ ub3lyp/def2tzvp, el. energy = -2367.961485 a.u.

C	-1.21740500	-2.76937000	-0.27917200
N	-1.39762800	-1.40782400	-0.26768300
H	-2.62624700	-4.51726600	-0.39486800
C	-2.75231700	-1.20323700	-0.35977700
C	-3.44846800	-2.47001000	-0.42144000
H	-4.52349600	-2.58355600	-0.49832300
C	-2.49374200	-3.44166200	-0.37038800
C	-3.37425600	0.04221800	-0.39182800
N	-1.36122000	1.44198400	-0.26438400
C	-1.14741900	2.79874600	-0.27564300
C	-2.40683400	3.50267100	-0.36537900
H	-2.51258300	4.58125600	-0.38915200
C	-3.38534600	2.55525000	-0.41675700
H	-4.45726600	2.69522000	-0.49337500
C	-2.72081400	1.27126000	-0.35673300

C	0.01396900	-3.41857300	-0.23156500
H	0.00090300	-4.50538800	-0.24936200
H	4.57699000	-2.69423600	-0.20449100
C	3.50216500	-2.55565600	-0.19604100
C	2.83360300	-1.27199900	-0.17634900
H	2.63135300	-4.58231700	-0.22927200
N	1.47258200	-1.44331400	-0.17253700
C	1.26048400	-2.79997900	-0.19878800
C	2.52374100	-3.50384200	-0.21052700
C	0.09943000	3.41736400	-0.23064500
H	0.11332400	4.50417100	-0.24734600
H	4.64341900	2.58057600	-0.20853500
C	3.56551800	2.46832500	-0.20018600
C	2.61080700	3.44066800	-0.21378500
H	2.74499800	4.51617100	-0.23183200
C	1.33070200	2.76828400	-0.20141100
N	1.50934300	1.40685100	-0.17676200
C	2.86541200	1.20182700	-0.17997700
C	3.48871900	-0.04331100	-0.17161400
H	4.57535800	-0.05754600	-0.17234500
H	-4.45874300	0.05628800	-0.46147200
Fe	0.04743300	-0.00035500	0.01627800
O	0.04842500	-0.00110900	2.02413100
C	-1.07719000	0.01064600	2.80295500
H	-0.83517400	0.00867700	3.89323100
H	-1.73093400	0.90378900	2.65498900
H	-1.74866700	-0.86935100	2.65574400

[5]FP⁻ ub3lyp/def2tzvp, el. energy = -2367.975132 a.u.

N	-1.52823000	1.33341900	-0.30945700
Fe	0.03590700	-0.00044300	0.39483500
C	-3.36406900	-0.29320500	-0.46693100
C	0.35148200	-3.42084300	-0.32559700
C	-0.95277000	-2.90648300	-0.36306100
C	-2.15981700	-3.70038800	-0.51302600
C	-3.20451900	-2.82243100	-0.55532100
C	-2.63574400	-1.49129900	-0.43067600
N	-1.27801000	-1.58089100	-0.30603700
H	-2.19275200	-4.78157100	-0.58810300
H	-4.25941000	-3.04523000	-0.67037800
C	-3.63905100	2.22471000	-0.56083600
C	-1.43503200	2.69509100	-0.37116000
C	-2.76102000	3.26958100	-0.52188000
H	-4.71655800	2.26219200	-0.67601600
H	-2.98008900	4.32860200	-0.60037200
C	2.88223300	-3.26791400	-0.32155000
C	2.96451900	-1.00969300	-0.23796000
C	3.76083100	-2.22341700	-0.29783500
H	3.10595000	-4.32747400	-0.37574500
H	4.84395800	-2.26123100	-0.32875100
C	-0.23674400	3.42340300	-0.34088100
C	3.47872200	0.29510300	-0.23454200
C	3.32681700	2.82493200	-0.31744500
C	1.06711800	2.90870900	-0.29561300
C	2.28246700	3.70343700	-0.34703700
H	4.38753700	3.04738100	-0.35000900
H	2.32149100	4.78525400	-0.40832700
C	2.74933300	1.49302800	-0.24774800
C	-2.85041300	1.01157000	-0.43385200
C	1.54871800	-2.69194800	-0.27662000
N	1.63755800	-1.33038100	-0.21711100
N	1.38679000	1.58290400	-0.22816200
O	0.03038700	-0.00341700	2.29568000
C	-1.05461400	-0.01451000	3.14621900
H	-1.69885000	-0.91303000	3.03360100
H	-0.73719700	-0.00367700	4.21123000
H	-1.72550100	0.86276400	3.02279400
H	-4.44317900	-0.38573800	-0.56925600
H	0.44539900	-4.50343600	-0.37654600
H	4.56246900	0.38802100	-0.25261800
H	-0.32739000	4.50607900	-0.39716500

[1cs]FP⁻-EDA b3lyp/def2tzvp, e. e. = -2784.108251 a.u.

C	-0.56170400	3.07264600	-0.38664900
N	-0.72982600	1.76904300	-0.78558000
H	-1.83663200	4.92186500	-0.53017600
C	-1.96583000	1.71760900	-1.37892200
C	-2.59435000	3.02209900	-1.35787500
H	-3.57418200	3.24526400	-1.76442100
C	-1.72192200	3.86424100	-0.73790600
C	-2.55444900	0.57034900	-1.90370300
N	-0.74584900	-1.01542500	-1.40882700
C	-0.61328200	-2.37473300	-1.56660500
C	-1.80450000	-2.93316900	-2.16796200
H	-1.94989000	-3.98335000	-2.39317100
C	-2.66201000	-1.89486800	-2.37377500
H	-3.65671900	-1.91654200	-2.80399100
C	-1.99498700	-0.70406800	-1.89174300
C	0.55923300	3.56822200	0.27267700
H	0.55783100	4.62442800	0.53099900
H	4.68782100	2.35308200	1.95175400
C	3.71093100	2.34007800	1.48222700
C	3.09221800	1.17250700	0.89090300
H	2.93911100	4.40198900	1.63414300
N	1.85481000	1.48700300	0.38719500
C	1.68052200	2.82694600	0.63489600
C	2.83169900	3.36869800	1.32466600
C	0.50325900	-3.11964000	-1.19718400
H	0.47073900	-4.18975000	-1.38626500
H	4.65541900	-2.78159600	0.79877600
C	3.68459900	-2.55673600	0.37245300
C	2.78794000	-3.41207700	-0.19123200
H	2.87169200	-4.48418100	-0.32668000
C	1.64702900	-2.61412200	-0.58781500
N	1.85103000	-1.29207100	-0.27176500
C	3.08825200	-1.23794700	0.32361300
C	3.67378700	-0.09188200	0.85239100
H	4.66039300	-0.19848400	1.29636900
C	-0.68282800	-0.36525700	2.48128100
C	-1.97158900	-1.04377500	2.49832100
O	-2.16846400	-2.18803300	2.87481500
O	-2.93255100	-0.20845000	2.04373100
C	-4.25570400	-0.76757700	1.91091600
H	-4.50688200	-1.31821600	2.82267400
H	-4.25177600	-1.47730600	1.07605200
N	1.25436900	-1.63946200	3.24932200
N	0.35988500	-1.04248800	2.88365800
H	-3.54617000	0.67347900	-2.33718200
Fe	0.62578300	0.26782100	-0.66685000
C	-5.21248400	0.38216200	1.66070700

H	-4.93402700	0.93274100	0.75629600
H	-6.23026900	-0.00496800	1.53198300
H	-5.21402500	1.08230900	2.50346100
H	-0.50648900	0.62583400	2.08561100
O	1.48540300	0.78916600	-2.27939100
C	1.45543000	-0.05225700	-3.36807700
H	2.13499300	0.33726500	-4.15667200
H	1.79923200	-1.08871800	-3.16142200
H	0.45786400	-0.15085900	-3.85494200

^{1os}FP^{-}-EDA ub3lyp/def2tzvp, e. e. = -2784.110044 a.u.

C	-0.44550000	3.13791300	-0.19014600
N	-0.66026100	1.86614200	-0.65913600
H	-1.65318100	5.03705400	-0.24130400
C	-1.88995400	1.88972200	-1.26398000
C	-2.47267500	3.21174800	-1.17196700
H	-3.44076100	3.49304900	-1.57046800
C	-1.57355300	3.98861400	-0.50450600
C	-2.50601200	0.79561900	-1.86864000
N	-0.73940900	-0.87139100	-1.49725300
C	-0.64219000	-2.21769200	-1.75800800
C	-1.84511800	-2.69451500	-2.40762500
H	-2.01789200	-3.71950200	-2.71472300
C	-2.67479800	-1.62193400	-2.52874900
H	-3.66851500	-1.58393300	-2.96013800
C	-1.97840100	-0.48979300	-1.95310400
C	0.69051500	3.55089400	0.50239200
H	0.72818500	4.58967400	0.82116900
H	4.73228400	2.07207700	2.18278500
C	3.76754500	2.12665300	1.69164600
C	3.11681200	1.02074800	1.01905100
H	3.07648100	4.20556500	1.94992500
N	1.90810900	1.41863000	0.50407400
C	1.77866700	2.74631300	0.82953400
C	2.93511500	3.19796400	1.57620800
C	0.44402900	-3.02392200	-1.43067000
H	0.38400900	-4.07320300	-1.70821700
H	4.54057500	-2.98067400	0.70943000
C	3.59062800	-2.69128100	0.27513300
C	2.68904300	-3.47079200	-0.38544100
H	2.74788800	-4.53037100	-0.60553900
C	1.58666900	-2.60801500	-0.75138500
N	1.81946500	-1.32511800	-0.32182900
C	3.03642900	-1.35497600	0.31125900
C	3.64660500	-0.26354700	0.92583800
H	4.61520500	-0.43451900	1.38907900
C	-0.77764000	-0.60359800	2.47916000
C	-2.09971200	-1.21246100	2.43261300
O	-2.36587500	-2.35785900	2.76077900
O	-3.00261600	-0.31377800	1.97879200
C	-4.34738000	-0.80321300	1.79498000
H	-4.65618700	-1.35034400	2.69089000
H	-4.35102500	-1.50349300	0.95212300
N	1.06533300	-2.02393300	3.22121800
N	0.21562100	-1.35807800	2.86688900
H	-3.49241200	0.95589600	-2.29706900
Fe	0.66610600	0.31853300	-0.65821700
C	-5.23835700	0.39457000	1.52872500
H	-4.90389200	0.93999500	0.64046800
H	-6.26883100	0.05856900	1.36281100
H	-5.23348800	1.08520800	2.37923800
H	-0.53651400	0.38815700	2.12197700
O	1.58024100	0.94328200	-2.24784800
C	1.59123400	0.17882800	-3.38978700
H	2.26859900	0.62755900	-4.15097800
H	1.96028900	-0.86212800	-3.24705100
H	0.60243000	0.08350700	-3.89749100

^{3}FP^{-}-EDA ub3lyp/def2tzvp, e. e. = -2784.114743 a.u.

C	-1.81283900	-0.93906300	-2.18947300
N	-1.06701400	-1.31366800	-1.10386400
H	-3.69231900	-1.74083000	-3.12652400
C	-1.67846000	-2.42175700	-0.58189400
C	-2.86182600	-2.74429500	-1.34214300
H	-3.52613300	-3.57580100	-1.13729700
C	-2.94490600	-1.81986400	-2.34564500
C	-1.21123900	-3.14540100	0.51680800
N	0.81435800	-1.83009200	0.99348700
C	1.72534000	-1.85221800	2.02605500
C	1.43816100	-2.95067600	2.92755900
H	2.01171200	-3.18386000	3.81713000
C	0.32777900	-3.57115500	2.44838900
H	-0.20154100	-4.42196800	2.86132200
C	-0.06888900	-2.85478000	1.25222800
C	-1.50884600	0.12962900	-3.03534200
H	-2.18851600	0.31717500	-3.86237000
H	1.37483000	3.68463800	-3.61050000
C	0.90244000	2.77674400	-3.25398700
C	1.34639100	2.01181500	-2.10469700
H	-0.81310700	2.42177400	-4.59132700
N	0.53599900	0.91611300	-1.91833300
C	-0.42669300	0.98970800	-2.89784900
C	-0.19468500	2.14335800	-3.74593300
C	2.73877100	-0.92467900	2.23400900
H	3.38579000	-1.08149900	3.09298800
H	4.53515500	2.99206200	0.54948800
C	3.91844300	2.11517900	0.70871000
C	4.01767600	1.17783000	1.69838700
H	4.73148200	1.13317400	2.51261000
C	2.97207500	0.21287900	1.45884100
N	2.24047800	0.56973300	0.35659700
C	2.81424800	1.71572600	-0.12938100
C	2.40316300	2.38551200	-1.28360600
H	2.94846000	3.28549300	-1.55606600
C	-1.12066300	2.02923800	1.38258500
C	-2.46379000	2.11511400	1.93467300
O	-2.80291100	2.79199500	2.89360700
O	-3.30072700	1.32529700	1.22417100
C	-4.67113600	1.29343900	1.67104900
H	-5.08956000	2.30500700	1.61819200
H	-4.69790800	0.97693600	2.71948300
N	0.62956900	3.31166200	2.50360100
N	-0.17856200	2.70970800	1.97678100
H	-1.80348100	-3.99850700	0.83823700
Fe	0.78582600	-0.56734500	-0.57916300
C	-5.41893800	0.32700300	0.77328700
H	-4.98287300	-0.67567500	0.82764500
H	-6.46826300	0.26918800	1.08616300
H	-5.38664800	0.65519300	-0.27101000
H	-0.83332300	1.41497200	0.53794700

O	1.91877000	-1.58082400	-1.84273200
C	2.55136500	-2.76789500	-1.56335500
H	2.90347100	-3.27687200	-2.49064100
H	3.46344700	-2.65636000	-0.93004300
H	1.91536600	-3.51754200	-1.04109500

^{5}**FP^{-}-EDA** ub3lyp/def2tzvp, e. e. = -2784.130894 a.u.

C	1.34963500	0.80352200	2.42175600
N	0.87028800	-0.22757800	1.66056000
H	3.12810800	0.99491200	3.79979700
C	1.71283800	-1.29005700	1.84559700
C	2.76853200	-0.92176700	2.77031900
H	3.57370500	-1.57269200	3.09189000
C	2.54286600	0.37661800	3.12848600
C	1.58692600	-2.53649600	1.21595600
N	-0.41552600	-2.12508100	-0.14635900
C	-1.09969500	-2.82982000	-1.09637700
C	-0.49041900	-4.13701700	-1.26993600
H	-0.82913100	-4.89902400	-1.96283600
C	0.57327000	-4.19035700	-0.41592800
H	1.27524100	-5.00436500	-0.27416400
C	0.61513200	-2.91543000	0.27855500
C	0.78828400	2.08689000	2.48167100
H	1.28512400	2.80205000	3.13349200
H	-2.51726000	4.82623400	0.72654800
C	-1.89124900	3.97275000	0.96114400
C	-2.03395100	2.64517100	0.38967800
H	-0.41721400	4.71968800	2.42120200
N	-1.07621800	1.81604500	0.90295000
C	-0.32329200	2.55895100	1.76917300
C	-0.82989200	3.91907300	1.81769500
C	-2.19807400	-2.34879500	-1.82340500
H	-2.63870300	-3.03424600	-2.54401300
H	-4.82716600	1.38805600	-2.62658000
C	-4.09086400	0.70273500	-2.22229200
C	-3.86637300	-0.59578300	-2.57646700
H	-4.38445900	-1.18235300	-3.32671700
C	-2.76560000	-1.06840800	-1.75414600
N	-2.34811400	-0.06793100	-0.92327200
C	-3.12689300	1.02411900	-1.18404800
C	-2.99015600	2.27547300	-0.56675800
H	-3.68566500	3.05007700	-0.88153700
C	1.53233400	1.00649700	-1.64752300
C	2.86595800	1.05049200	-2.22033400
O	3.16019700	1.44890700	-3.33799000
O	3.76599900	0.58018000	-1.32254500
C	5.13896400	0.56488900	-1.75573100
H	5.45682700	1.58907600	-1.98266300
H	5.21996600	-0.01840400	-2.67931300
N	-0.30315700	1.80405200	-3.04764500
N	0.54568200	1.43014300	-2.39148300
H	2.34368400	-3.27952200	1.45758800
Fe	-1.15642000	-0.35637400	0.87209300
C	5.96094800	-0.04200800	-0.63444500
H	5.63984100	-1.06779600	-0.42445300
H	7.01966000	-0.06166200	-0.91884400
H	5.86040100	0.54257200	0.28626500
H	1.28818800	0.65253700	-0.65223600
O	-2.33670400	-0.89549200	2.25293200
C	-2.30337200	-2.02988600	3.03670800
H	-3.13029100	-2.03637900	3.77834500
H	-2.40761100	-2.97236500	2.45732800
H	-1.36811300	-2.13513000	3.62795200

^{1os}**TS1^{-}** ub3lyp/def2tzvp, e. e. = -2784.095122 a.u.
im. frequency -1120.47

C	-1.91382400	-1.46693500	-1.42243100
N	-0.63006300	-1.44838200	-0.93610600
H	-3.31076000	-3.11734200	-2.04440100
C	-0.23036100	-2.76221500	-0.86657300
C	-1.29509100	-3.63264100	-1.30906100
H	-1.23472600	-4.71422500	-1.34161300
C	-2.33733500	-2.83108300	-1.66359300
C	1.02594600	-3.19609000	-0.46104000
N	2.04018300	-0.99221500	-0.08124400
C	3.29199600	-0.57819200	0.29740900
C	4.15249200	-1.72025300	0.51322300
H	5.18933500	-1.66225400	0.82271600
C	3.39730700	-2.83079600	0.27822900
H	3.68951800	-3.87214200	0.34572200
C	2.08237200	-2.36835900	-0.09394700
C	-2.71590600	-0.34974500	-1.62839300
H	-3.71133700	-0.51948900	-2.03045800
H	-2.87102900	4.22802600	-1.01337600
C	-2.54994600	3.19327800	-1.04585600
C	-1.24832000	2.72027900	-0.63755300
H	-4.25078800	2.05717100	-1.87938800
N	-1.16335500	1.35995300	-0.80038900
C	-2.36437300	0.96436900	-1.32208800
C	-3.24333100	2.10245200	-1.48238700
C	3.67715600	0.74349100	0.50831900
H	4.70703300	0.92040900	0.80632300
H	2.06295700	5.07205200	0.62404500
C	2.15053500	3.99755900	0.51151200
C	3.25054700	3.21243000	0.69073100
H	4.25004400	3.50771700	0.98789900
C	2.83946300	1.85312200	0.41248300
N	1.51504100	1.81735100	0.06821400
C	1.07423600	3.12021900	0.11459900
C	-0.21439600	3.54123400	-0.19166900
H	-0.42283000	4.60516800	-0.11190100
C	-0.24130000	0.07401500	1.84717700
C	-1.45813300	-0.73688800	2.02562200
O	-1.49552600	-1.92449700	2.30341600
O	-2.57201800	0.03115000	1.88717100
C	-3.82605500	-0.66550300	1.99376100
H	-3.84375700	-1.23685900	2.92831600
H	-3.91121100	-1.37844500	1.16667500
N	1.46934300	-1.32180600	2.90394700
N	0.81954700	-0.44077100	2.53933400
H	1.20128000	-4.26824000	-0.43891500
Fe	0.45961700	0.18305400	-0.50805500
C	-4.93264100	0.37218500	1.95351600
H	-4.89705400	0.94567800	1.02158900
H	-5.90924700	-0.12232500	2.02146900
H	-4.84472600	1.07367600	2.79092100
H	-0.33070200	1.14938700	1.92389600
O	0.94102700	0.49161900	-2.27030900
C	1.38930200	-0.52327800	-3.10143100
H	1.66350600	-0.08347900	-4.07923900

H	2.28998200	-1.04381400	-2.72500400
H	0.62968300	-1.30109500	-3.30771000

^{3}**TS1**$^{-}$ ub3lyp/def2tzvp, e. e. = - 2784.096722 a.u.
im. frequency -426.84

C	-2.37490100	0.06921400	-1.60289900
N	-1.25483800	-0.61518600	-1.19870900
H	-4.36459800	-0.57323800	-2.42119500
C	-1.53676700	-1.95752200	-1.38307600
C	-2.86972000	-2.11354900	-1.90733100
H	-3.33376700	-3.06589500	-2.13518500
C	-3.38642500	-0.86047300	-2.05379900
C	-0.65679200	-2.99956600	-1.14879000
N	1.26542800	-1.65279500	-0.40977300
C	2.55094600	-1.96109700	-0.03718800
C	2.75884500	-3.38183900	-0.09939300
H	3.68530900	-3.88107800	0.15767600
C	1.57666200	-3.93790600	-0.51149400
H	1.34030500	-4.98429000	-0.66369300
C	0.65647900	-2.85682200	-0.70221700
C	-2.54889100	1.44730100	-1.56119000
H	-3.49404700	1.84398400	-1.92039000
H	-0.49860200	5.35246100	-0.16680700
C	-0.70925300	4.31490600	-0.39829300
C	0.19362200	3.22426600	-0.16345700
H	-2.74098400	4.28781000	-1.27335900
N	-0.38404600	2.03366600	-0.55533500
C	-1.62065300	2.35826700	-1.04971300
C	-1.83907800	3.77876100	-0.95481400
C	3.51539000	-1.04088100	0.38689700
H	4.49511400	-1.43100600	0.64595500
H	4.16429100	3.43388100	1.31586000
C	3.72937200	2.48619900	1.02052100
C	4.31054200	1.25396800	1.00748600
H	5.31878200	0.97900400	1.29337200
C	3.30985100	0.32395200	0.53166400
N	2.13334100	0.98559200	0.27793500
C	2.37801600	2.31757000	0.54738300
C	1.47494500	3.35321700	0.36728900
H	1.80636500	4.35444300	0.62973600
C	-0.30193300	0.02300000	2.04584300
C	-1.49473000	-0.75947100	2.31802600
O	-1.53448200	-1.92983600	2.67191300
O	-2.61932400	-0.00330600	2.12831200
C	-3.86975900	-0.69621200	2.25562000
H	-3.91985800	-1.18691700	3.23440800
H	-3.93179700	-1.47769500	1.48949900
N	1.53454700	-1.34262400	2.86842800
N	0.87922200	-0.46786500	2.46186500
H	-1.01162400	-4.00838100	-1.34046800
Fe	0.48449200	0.19621900	-0.60196400
C	-4.97686500	0.32902800	2.08849000
H	-4.90885100	0.81637400	1.11011000
H	-5.95610600	-0.15890600	2.16616100
H	-4.91593500	1.10247300	2.86264800
H	-0.35300400	1.08856900	1.87923400
O	1.13754600	0.52340400	-2.24476800
C	1.24536600	-0.48698900	-3.20675600
H	1.41360600	-0.00184600	-4.18323500
H	2.09833900	-1.15907200	-3.01909600
H	0.33915300	-1.10390100	-3.29753100

^{5}**TS1**$^{-}$ ub3lyp/def2tzvp, e. e. = - 2784.079991 a.u.
im. frequency -221.19

C	-0.50937700	3.06111500	-0.30168000
N	-0.83720100	1.76122000	-0.57089800
H	-1.69851300	4.97573000	-0.41159600
C	-2.14465000	1.72729100	-0.96928200
C	-2.67745400	3.07779800	-0.95865000
H	-3.68990700	3.35034500	-1.23356900
C	-1.67016000	3.90008500	-0.54247600
C	-2.85896600	0.57486900	-1.33192300
N	-1.14520300	-1.14717100	-0.97620900
C	-1.04876200	-2.49517100	-1.16210900
C	-2.32177100	-2.99559400	-1.64748300
H	-2.53875900	-4.03223800	-1.87779500
C	-3.15710500	-1.91882300	-1.75993000
H	-4.18551600	-1.90676200	-2.10257400
C	-2.39889700	-0.75272200	-1.34217400
C	0.75048900	3.51876800	0.12014800
H	0.83992600	4.59076300	0.27966600
H	5.12662300	2.19657900	0.97509100
C	4.06804300	2.20011300	0.74206300
C	3.27784700	1.02410200	0.41809600
H	3.46032200	4.31530700	0.86920700
N	1.98981600	1.40637700	0.19002700
C	1.91283800	2.75835900	0.32968900
C	3.22347000	3.27260400	0.69121500
C	0.11237900	-3.25612200	-0.95202400
H	0.02739900	-4.32313000	-1.14296800
H	4.63024500	-3.04940300	0.09853800
C	3.59303500	-2.79296300	-0.08293400
C	2.57085200	-3.62472700	-0.43152800
H	2.60629900	-4.69593700	-0.59196100
C	1.37932900	-2.79981600	-0.55720500
N	1.70184400	-1.50756700	-0.27099100
C	3.03506600	-1.45260000	0.00950300
C	3.75675200	-0.29293900	0.33289700
H	4.81540200	-0.43192000	0.53899200
C	-0.06365400	0.01546700	1.76022100
C	-1.28475600	-0.77016000	2.07422500
O	-1.32900100	-1.93978400	2.41387600
O	-2.40213200	0.00308500	1.96932400
C	-3.65123600	-0.67559700	2.18454000
H	-3.61438400	-1.20421100	3.14334700
H	-3.79351200	-1.42593700	1.39902500
N	1.52072600	-1.47059800	2.78894800
N	1.05084600	-0.41324500	2.56259400
H	-3.88822900	0.72981800	-1.64740200
Fe	0.44452000	0.12231700	-0.46303900
C	-4.75418800	0.36675400	2.16591900
H	-4.77456900	0.89764300	1.20875300
H	-5.72730900	-0.11684100	2.31503300
H	-4.61230000	1.10498700	2.96329500
H	-0.20565900	1.08382200	1.88237600
O	1.05403500	0.24771900	-2.24957300
C	0.32113100	0.81763300	-3.27903500
H	0.87287600	0.68849200	-4.23111700
H	-0.67068800	0.35011300	-3.42899700
H	0.15220200	1.90490800	-3.16012600

^{1os}**INT1**$^-$ ub3lyp/def2tzvp, e. e. = - 2784.103969 a.u.

C	-2.12896400	-1.14494500	-1.41305800
N	-0.85041900	-1.30820400	-0.94642100
H	-3.72336400	-2.58219500	-2.08036000
C	-0.61340700	-2.65792500	-0.93422300
C	-1.78088800	-3.37307100	-1.39534900
H	-1.85353200	-4.45121300	-1.47302600
C	-2.71963000	-2.43379400	-1.69997400
C	0.58929300	-3.25841400	-0.57716500
N	1.86381800	-1.21508500	-0.11802900
C	3.16379800	-0.96528100	0.23098900
C	3.89225500	-2.20867700	0.35792400
H	4.93803800	-2.28557900	0.63037900
C	3.00842400	-3.20906800	0.09240400
H	3.17793600	-4.28000700	0.09667900
C	1.74339800	-2.57669300	-0.20840400
C	-2.78555700	0.07168300	-1.57589600
H	-3.79864900	0.04007100	-1.96755800
H	-2.33843600	4.62013000	-0.90629900
C	-2.15618300	3.55318500	-0.95597500
C	-0.92142200	2.90743800	-0.56551900
H	-3.99983400	2.65756700	-1.77501300
N	-1.01837500	1.55508500	-0.74594500
C	-2.26560600	1.32194200	-1.25484600
C	-2.98968200	2.56799300	-1.39309900
C	3.71409500	0.29155000	0.46407300
H	4.76497900	0.33018200	0.73690400
H	2.68340500	4.78992200	0.68025800
C	2.62814500	3.71520200	0.55364100
C	3.61845000	2.79120800	0.69434500
H	4.65521100	2.94882500	0.96682800
C	3.02595600	1.50075800	0.41358200
N	1.70243900	1.64878600	0.10629400
C	1.43524200	2.98895800	0.17583500
C	0.20875900	3.58152000	-0.11027200
H	0.14043900	4.66145700	-0.01288800
C	-0.17871900	0.11863600	1.69546000
C	-1.38080000	-0.69434900	1.95669100
O	-1.42970400	-1.87283800	2.26980800
O	-2.51905300	0.06803600	1.84669100
C	-3.75532800	-0.62952700	2.04763200
H	-3.72397200	-1.16174300	3.00517600
H	-3.88380700	-1.38176700	1.26161000
N	1.26690400	-1.29299100	2.97387500
N	0.95264600	-0.23437700	2.53439100
H	0.63611300	-4.34309000	-0.60649500
Fe	0.43507700	0.16443500	-0.44908300
C	-4.87596700	0.39499500	2.02194100
H	-4.89731900	0.92946100	1.06636300
H	-5.84368200	-0.10258600	2.16166500
H	-4.75175400	1.13365400	2.82203600
H	-0.36653600	1.18186700	1.80751100
O	0.95619800	0.43978500	-2.20545100
C	1.35768600	-0.57898400	-3.06061000
H	1.53058000	-0.15192000	-4.06794800
H	2.30292100	-1.06484000	-2.75104900
H	0.60455800	-1.38090200	-3.18089700

^{3}**INT1**$^-$ ub3lyp/def2tzvp, e. e. = -2784.102967 a.u.

C	-0.94760800	2.89208800	-0.58008700
N	-1.02931300	1.53816100	-0.75630300
H	-2.38067300	4.58926200	-0.93169700
C	-2.27233500	1.29082500	-1.26849200
C	-3.00832500	2.52908100	-1.41377100
H	-4.01787200	2.60698800	-1.79977400
C	-2.18745600	3.52409000	-0.97722900
C	-2.77851700	0.03453000	-1.58850400
N	-0.83199300	-1.32425000	-0.94692800
C	-0.58062800	-2.67168800	-0.93322300
C	-1.73768200	-3.39910200	-1.40073600
H	-1.79833800	-4.47793700	-1.47881600
C	-2.68484000	-2.46982500	-1.71025500
H	-3.68476700	-2.62904000	-2.09621300
C	-2.10987700	-1.17489500	-1.42037200
C	0.17418700	3.57890600	-0.12374500
H	0.09500200	4.65843300	-0.03043400
H	4.62317500	2.99435000	0.97020800
C	3.58887100	2.82563800	0.69511200
C	3.01003100	1.52853400	0.41696900
H	2.63430600	4.81502800	0.67034500
N	1.68586500	1.66164000	0.10472400
C	1.40555600	2.99917700	0.16814500
C	2.59007400	3.73941300	0.54714500
C	0.62713700	-3.25938900	-0.57034900
H	0.68568900	-4.34350100	-0.59957700
H	4.96017500	-2.23677600	0.65046700
C	3.91441600	-2.17169400	0.37490900
C	3.04308200	-3.18279000	0.10782300
H	3.22385800	-4.25084700	0.11513100
C	1.77219000	-2.56431900	-0.19779200
N	1.87662800	-1.20171900	-0.10965700
C	3.17307800	-0.93649400	0.24325400
C	3.71003700	0.32654700	0.47348200
H	4.75985800	0.37557600	0.74856200
C	-0.19098400	0.11898900	1.70926800
C	-1.39283700	-0.69160200	1.97479000
O	-1.44269100	-1.86410200	2.30986900
O	-2.53297600	0.06460000	1.83603300
C	-3.76928200	-0.63340000	2.03276600
H	-3.74181400	-1.16482600	2.99067700
H	-3.89384400	-1.38656600	1.24572700
N	1.25749600	-1.26969600	3.00437100
N	0.94588800	-0.21949700	2.53743300
H	-3.78952500	-0.00849700	-1.98437600
Fe	0.43664900	0.16330600	-0.45214200
C	-4.89097900	0.38987800	2.00104300
H	-4.90804000	0.92455100	1.04547000
H	-5.85896700	-0.10860000	2.13576800
H	-4.77151300	1.12855700	2.80185800
H	-0.37992900	1.18390400	1.80152000
O	0.96137400	0.43832400	-2.20327500
C	1.39315300	-0.57745200	-3.04805500
H	1.52114200	-0.16498700	-4.06600400
H	2.36771400	-1.00670500	-2.75115200
H	0.68104000	-1.41765200	-3.13104000

^{5}**INT1**$^-$ ub3lyp/def2tzvp, e. e. = -2784.079520 a.u.

C	-0.55048500	3.03750200	-0.35180900
N	-0.85349100	1.72782200	-0.60565400

H	-1.77352900	4.92751600	-0.48899300
C	-2.15938400	1.66518000	-1.00644200
C	-2.71642800	3.00511600	-1.01380600
H	-3.73337500	3.25477500	-1.29368800
C	-1.72537800	3.85107000	-0.60636000
C	-2.85326300	0.49748300	-1.35829100
N	-1.10679700	-1.18633200	-0.98801500
C	-0.98652500	-2.53426400	-1.15744800
C	-2.24835200	-3.06305000	-1.64188100
H	-2.44529900	-4.10614100	-1.86045700
C	-3.10342100	-2.00379200	-1.76576700
H	-4.13179800	-2.01408100	-2.10842600
C	-2.36790300	-0.82007100	-1.35778100
C	0.69776500	3.52589600	0.06856700
H	0.76590100	4.60161100	0.21194200
H	5.08829200	2.28923200	0.97278100
C	4.03137900	2.27286100	0.73305600
C	3.26109200	1.07923700	0.42574200
H	3.39029400	4.38035700	0.81823300
N	1.96973900	1.43741900	0.18284600
C	1.87095600	2.78969300	0.29802100
C	3.17120200	3.33101500	0.65780900
C	0.18352800	-3.27320700	-0.92549000
H	0.11947300	-4.34385800	-1.10301100
H	4.67860400	-2.97379600	0.19364400
C	3.64028600	-2.73777200	-0.00772500
C	2.63750000	-3.59129600	-0.36025300
H	2.69281600	-4.66385700	-0.50483100
C	1.43509900	-2.78902500	-0.51681600
N	1.73169000	-1.48718400	-0.24307300
C	3.05986100	-1.40627300	0.05699700
C	3.75993700	-0.23129600	0.37048900
H	4.81797400	-0.35109100	0.59073100
C	-0.07935100	0.02218000	1.70749300
C	-1.30185500	-0.75914000	2.01819400
O	-1.36543100	-1.93436600	2.33373200
O	-2.41524700	0.03377300	1.95771200
C	-3.66291400	-0.62947100	2.21398200
H	-3.60542700	-1.14884400	3.17722800
H	-3.83809900	-1.38792300	1.44275100
N	1.39959000	-1.44323500	2.88421100
N	1.06200500	-0.36445000	2.52307800
H	-3.88553200	0.63088200	-1.67344200
Fe	0.45132200	0.11705000	-0.44903400
C	-4.75623700	0.42352800	2.21613200
H	-4.80584200	0.93906700	1.25147700
H	-5.72855700	-0.04659100	2.40795600
H	-4.57809100	1.17358600	2.99499500
H	-0.23599300	1.08931800	1.83071500
O	1.07179200	0.24109500	-2.20645000
C	0.32343700	0.75434200	-3.25862000
H	0.89027900	0.61034100	-4.19779500
H	-0.64873600	0.24778200	-3.39611500
H	0.12093700	1.83743600	-3.17245700

^{1cs}**TS2**$^{-}$ b3lyp/def2tzvp, e. e. = -2784.086946 a.u.
im. frequency -434.45

C	-1.07963800	2.77827200	-0.63611500
N	-1.09472500	1.41663000	-0.75412500
H	-2.58643500	4.38998200	-1.09380600
C	-2.31474800	1.08731000	-1.27418700
C	-3.10712300	2.28283000	-1.48954900
H	-4.10930000	2.29726100	-1.90242400
C	-2.34258600	3.33390300	-1.08231700
C	-2.75462400	-0.20947900	-1.53565700
N	-0.74623200	-1.43884800	-0.83813600
C	-0.41758400	-2.76440200	-0.78594400
C	-1.53590300	-3.57817900	-1.21641900
H	-1.53351200	-4.66094300	-1.26363200
C	-2.53627100	-2.71695700	-1.55279600
H	-3.52528600	-2.94755900	-1.93185800
C	-2.03097600	-1.37970200	-1.31083200
C	0.00614100	3.53426500	-0.19473600
H	-0.12897800	4.61189700	-0.14380100
H	4.50377500	3.26593100	0.83472100
C	3.47483000	3.02185000	0.59697100
C	2.97140500	1.68232700	0.36281700
H	2.39602400	4.94537800	0.53733000
N	1.63310600	1.71841100	0.08986800
C	1.27180700	3.03836000	0.11513000
C	2.41554800	3.86497800	0.45098700
C	0.83748000	-3.26157900	-0.43687700
H	0.96711900	-4.34071700	-0.45087300
H	5.17055000	-1.95611200	0.49336900
C	4.10566200	-1.95794800	0.29194800
C	3.28192700	-3.02442800	0.09836600
H	3.53288600	-4.07875500	0.09736700
C	1.95594800	-2.49048500	-0.13243500
N	1.97740600	-1.11827400	-0.06027900
C	3.28118700	-0.77162600	0.20040900
C	3.74787300	0.52523900	0.39900900
H	4.80669000	0.64252900	0.61552400
C	-0.27282500	0.18558600	1.48830200
C	-1.46327600	-0.59743400	1.96002400
O	-1.45182200	-1.72383300	2.42381600
O	-2.60151600	0.11895000	1.79341800
C	-3.83528500	-0.57699800	2.06184600
H	-3.77814400	-1.03963700	3.05248800
H	-3.95806200	-1.37794400	1.32506400
N	1.80534100	-1.03508200	2.74061400
N	0.89061200	-0.39058400	2.64659600
H	-3.75787600	-0.31946800	-1.94025800
Fe	0.44918700	0.13225600	-0.36218900
C	-4.96110100	0.43662700	1.98013000
H	-4.98876100	0.91158900	0.99425400
H	-5.92304100	-0.06090900	2.15289400
H	-4.83970000	1.22140600	2.73520900
H	-0.32803600	1.21678900	1.84416800
O	0.97202500	0.43650400	-2.19954100
C	0.85359700	-0.51861700	-3.17985100
H	1.36440300	-0.17691800	-4.10701100
H	1.31349200	-1.50045400	-2.92744000
H	-0.19350500	-0.74302600	-3.48703100

^{1os}**TS2**$^{-}$ ub3lyp/def2tzvp, e. e. = -2784.095544 a.u.
im. frequency -457.50

C	-0.14495500	3.17257800	-0.36781200
N	-0.58876800	1.91266600	-0.65531000
H	-1.07023700	5.21650100	-0.54439400
C	-1.85763000	2.04990400	-1.14502600

C	-2.23380300	3.44855000	-1.16805000
H	-3.18811900	3.82731000	-1.51470200
C	-1.17127300	4.14598300	-0.67917100
C	-2.68311800	1.00565200	-1.55420300
N	-1.17448700	-0.87966900	-1.09112400
C	-1.28873000	-2.23846000	-1.21373500
C	-2.59450300	-2.58400300	-1.72989100
H	-2.93743700	-3.59442000	-1.91825900
C	-3.26003400	-1.41080100	-1.92471600
H	-4.26382700	-1.25936200	-2.30407800
C	-2.36306000	-0.34982300	-1.51818900
C	1.11675300	3.48558900	0.13277700
H	1.33417100	4.53389300	0.31832400
H	5.21885500	1.62755100	1.16149900
C	4.18289100	1.76849900	0.87667900
C	3.28080600	0.70425500	0.48745700
H	3.80185600	3.93644500	1.02539100
N	2.04815000	1.21697200	0.19256900
C	2.13805900	2.56994500	0.37141500
C	3.47118500	2.92711600	0.81044800
C	-0.28554100	-3.15697800	-0.91755800
H	-0.51486400	-4.20964400	-1.05698200
H	4.14510100	-3.45555900	0.34939500
C	3.15754500	-3.08655500	0.09913700
C	2.05230900	-3.79670400	-0.25833100
H	1.94398000	-4.86933400	-0.36614800
C	0.99592900	-2.83400700	-0.48288600
N	1.45823500	-1.56471900	-0.25457100
C	2.77415100	-1.69086300	0.10201500
C	3.62750400	-0.64344100	0.44029500
H	4.64770000	-0.90301300	0.70921500
C	-0.25646700	0.15276100	1.48939200
C	-1.40425000	-0.67133200	1.90707800
O	-1.42672300	-1.87334200	2.11959000
O	-2.53686500	0.09923100	2.05262000
C	-3.74084400	-0.61621100	2.36504700
H	-3.63480400	-1.09939900	3.34450200
H	-3.90023200	-1.40987200	1.62702800
N	1.26956800	-1.36928400	3.00971500
N	1.04704700	-0.30169100	2.66752400
H	-3.67074000	1.27004600	-1.92286300
Fe	0.44135800	0.16243600	-0.45308100
C	-4.88414000	0.38293300	2.36152500
H	-4.98573300	0.85305400	1.37724800
H	-5.82775600	-0.12184800	2.60268000
H	-4.71937100	1.17398400	3.10199600
H	-0.36964900	1.18749500	1.80926100
O	1.05370600	0.43045300	-2.20812100
C	1.06317300	-0.53832400	-3.19672000
H	1.56742300	-0.14166600	-4.09959700
H	1.60656700	-1.46135000	-2.91580800
H	0.05088200	-0.85239900	-3.51982100

^{3}**TS2**$^{-}$ ub3lyp/def2tzvp, e. e. = -2784.091443 a.u.
im. frequency -437.04

C	-1.14994600	2.77963500	-0.73620300
N	-1.11362300	1.42128200	-0.88800500
H	-2.70944500	4.34365900	-1.16801900
C	-2.31612700	1.05925500	-1.42593600
C	-3.14951300	2.22886800	-1.61735000
H	-4.15055500	2.21472600	-2.03205600
C	-2.42604300	3.29787000	-1.18416700
C	-2.69954600	-0.24172400	-1.74069500
N	-0.66051600	-1.41995900	-1.03287600
C	-0.29624000	-2.73893800	-0.98858900
C	-1.36714100	-3.56856900	-1.49205400
H	-1.33163100	-4.64921000	-1.56100800
C	-2.37958100	-2.72810000	-1.84397600
H	-3.34813700	-2.97601100	-2.26206900
C	-1.92887400	-1.38574700	-1.54770300
C	-0.10589500	3.56150100	-0.24950400
H	-0.27302200	4.63313700	-0.18362300
H	4.29301300	3.35537400	1.14674700
C	3.29701800	3.09826600	0.80601100
C	2.84335500	1.75400800	0.52074600
H	2.19344900	5.00414800	0.66479100
N	1.53858800	1.77532400	0.11277800
C	1.14832900	3.08695000	0.12646000
C	2.24210100	3.92617200	0.56573500
C	0.93270200	-3.22104200	-0.54714400
H	1.07962600	-4.29726800	-0.55511900
H	5.06596700	-1.83928900	0.98131000
C	4.04340500	-1.86209900	0.62369200
C	3.28022700	-2.94313600	0.30415900
H	3.54557300	-3.99285300	0.34306100
C	1.99253600	-2.43188100	-0.11397600
N	1.98150800	-1.06453100	-0.04347600
C	3.21814700	-0.69232100	0.40879800
C	3.63082800	0.61369200	0.65741100
H	4.64967200	0.75430400	1.00778200
C	-0.32904900	0.13407400	1.47228200
C	-1.47806500	-0.70463400	1.83099500
O	-1.49793900	-1.91823200	1.97268300
O	-2.61843700	0.05121800	2.02123400
C	-3.81774900	-0.69113400	2.27790300
H	-3.72882800	-1.21182000	3.24006600
H	-3.94804400	-1.45847800	1.50676300
N	1.10956200	-1.34518200	3.16699100
N	0.97362700	-0.30078300	2.72261100
H	-3.69208500	-0.37989100	-2.16092400
Fe	0.44287900	0.17606400	-0.48402700
C	-4.97725200	0.28931600	2.28439700
H	-5.05739800	0.80111800	1.31899000
H	-5.91885100	-0.24087900	2.47424000
H	-4.84873700	1.04954600	3.06333100
H	-0.46301100	1.16706300	1.79145600
O	1.00742300	0.47143600	-2.23441300
C	1.70288800	-0.45352400	-2.99795800
H	1.76015000	-0.09727700	-4.04444500
H	2.74602500	-0.60580700	-2.66013800
H	1.22594100	-1.45093300	-3.02863400

^{5}**TS2**$^{-}$ ub3lyp/def2tzvp, e. e. = -2784.070792 a.u.
im. frequency -442.21

C	-0.89551500	2.90478400	-0.53783500
N	-1.02070300	1.56194200	-0.75960400
H	-2.34557200	4.61691300	-0.76999000
C	-2.29547600	1.31755700	-1.18871300
C	-3.01969200	2.57408300	-1.25272600
H	-4.05173700	2.68239100	-1.56578700

C	-2.15647200	3.55266200	-0.85053000
C	-2.82144100	0.05744100	-1.51431700
N	-0.87567400	-1.36873500	-1.06306500
C	-0.58418400	-2.69648600	-1.15369300
C	-1.75800500	-3.40442500	-1.63265400
H	-1.81723300	-4.47408000	-1.79731900
C	-2.73739900	-2.47069900	-1.82146200
H	-3.74994700	-2.62937000	-2.17428100
C	-2.16698400	-1.18437500	-1.46159500
C	0.26291400	3.55668000	-0.08450100
H	0.19186600	4.63654100	0.02210600
H	4.71207200	2.91371300	1.10331800
C	3.68154100	2.75528500	0.80693000
C	3.08357000	1.46323400	0.51311300
H	2.78211000	4.76793800	0.75681600
N	1.77581300	1.64684300	0.18032500
C	1.50409200	2.97978500	0.22961900
C	2.70410700	3.69368600	0.63424200
C	0.65493900	-3.27555200	-0.83969800
H	0.72705100	-4.35297700	-0.96634800
H	4.99924800	-2.39006200	0.54153200
C	3.95669800	-2.29017200	0.26265300
C	3.09002800	-3.27229500	-0.11606600
H	3.28384600	-4.33435700	-0.21117600
C	1.81368500	-2.62800700	-0.38483000
N	1.93373200	-1.29166300	-0.15176100
C	3.21625400	-1.03833400	0.23214800
C	3.74335300	0.22440700	0.54368100
H	4.79101600	0.24576200	0.83425700
C	-0.21511600	0.05609800	1.49312600
C	-1.38291100	-0.75267800	1.89808700
O	-1.42429500	-1.95791500	2.08340100
O	-2.49014400	0.03960600	2.09387500
C	-3.70310300	-0.65141400	2.42929300
H	-3.58795400	-1.13558700	3.40730400
H	-3.89027200	-1.44254200	1.69525400
N	1.34057500	-1.48963300	2.98768000
N	1.08453100	-0.40917600	2.71625700
H	-3.85576700	0.04296100	-1.84982700
Fe	0.46005500	0.13054500	-0.47437700
C	-4.82509000	0.37136500	2.44419200
H	-4.93284800	0.84282300	1.46131700
H	-5.77472700	-0.11298100	2.70267600
H	-4.63032100	1.15965000	3.18015300
H	-0.32854800	1.09509000	1.79836800
O	1.15786800	0.27817000	-2.20516600
C	0.40252300	0.63239700	-3.31481700
H	1.01710900	0.50452000	-4.22615700
H	-0.49813400	0.00667200	-3.45239300
H	0.06805700	1.68670400	-3.30360800

N2 b3lyp/def2tzvp, el. energy = -109.567864 a.u.

N	0.00000000	0.00000000	0.55256300
N	0.00000000	0.00000000	-0.55256300

^{1cs}**TC**$^-$ b3lyp/def2tzvp, e. e. = -2674.539272 a.u.

C	-1.03555300	2.65805200	-0.73897200
N	-1.04441900	1.29212000	-0.80590200
H	-2.55227100	4.24009200	-1.25565900
C	-2.27199500	0.93412800	-1.28837300
C	-3.07066700	2.11567300	-1.54665200
H	-4.07927000	2.10699000	-1.94345800
C	-2.30342700	3.18654700	-1.20224200
C	-2.70283900	-0.37424100	-1.49775800
N	-0.66665600	-1.53010500	-0.77336100
C	-0.29097800	-2.84363200	-0.70185100
C	-1.37398900	-3.69681000	-1.14638100
H	-1.33252900	-4.77900000	-1.18745300
C	-2.40353900	-2.87438700	-1.49066600
H	-3.38143400	-3.14148400	-1.87433600
C	-1.94770700	-1.51962500	-1.25456500
C	0.02885700	3.44520200	-0.30414000
H	-0.11952300	4.52211100	-0.31490600
H	4.40778500	3.28194500	1.16728200
C	3.41145600	3.01504200	0.83423700
C	2.94024100	1.65867500	0.63011900
H	2.34248300	4.92160000	0.53584800
N	1.64349800	1.67017200	0.20274400
C	1.27290100	2.98248600	0.12005600
C	2.37418500	3.83835600	0.51728300
C	0.95417600	-3.30880100	-0.28216200
H	1.10232700	-4.38558700	-0.27997000
H	5.12697500	-1.95988800	1.17617000
C	4.09727700	-1.97338600	0.83804800
C	3.32200900	-3.04682700	0.52309400
H	3.58148000	-4.09868000	0.55183900
C	2.03135300	-2.52214600	0.11923100
N	2.03718000	-1.15729800	0.18948200
C	3.28006200	-0.79458600	0.62468800
C	3.70700500	0.51371900	0.84067100
H	4.72779400	0.65437500	1.18664600
C	-0.04662900	-0.00712700	1.47118700
C	-1.24383900	-0.60802500	2.08732000
O	-1.23252200	-1.69927100	2.63927100
O	-2.35916100	0.15283800	1.99189200
C	-3.56324900	-0.41553700	2.54482200
H	-3.37511400	-0.72994500	3.57710400
H	-3.83060600	-1.30870000	1.96919300
H	-3.70815900	-0.51293300	-1.88715100
Fe	0.47372200	0.06857500	-0.24826200
C	-4.64788600	0.64273300	2.46953600
H	-4.81314100	0.95971000	1.43460300
H	-5.58897600	0.24081900	2.86418200
H	-4.37715800	1.52576400	3.05902200
H	0.67072700	0.29855800	2.24663800
O	1.24004000	0.21620700	-2.03247500
C	0.59658700	-0.07496600	-3.21015200
H	1.26132500	0.12464500	-4.07983000
H	0.28365600	-1.13807600	-3.31492200
H	-0.32010800	0.52920700	-3.39453100

^{1os}**TC**$^-$ ub3lyp/def2tzvp, e. e. = -2674.548627 a.u.

N	-0.75760000	1.62041600	-0.91872000
Fe	0.41759800	0.04102300	-0.46106300
C	-0.33136000	-0.14164900	1.31794700
C	0.65851000	3.41192900	-0.01025100
C	3.50715700	-0.36594800	1.01602200
C	3.02357000	0.93400900	0.89352600
C	3.73096100	2.11997900	1.33135400
C	2.92655000	3.18139400	1.04818500

C	1.72971900	2.64175700	0.43503500
N	1.81646100	1.28001000	0.35182400
H	4.71265500	2.11889800	1.79173700
H	3.11102500	4.23435000	1.22547900
C	-1.56044000	3.77403200	-1.13012700
C	-1.96147900	1.60071200	-1.56824200
C	-2.47302700	2.94707100	-1.71047700
H	-1.59054200	4.85322500	-1.03749400
H	-3.41007300	3.20501000	-2.18967400
C	3.35729600	-2.86152400	0.73881600
C	1.32514000	-2.85756800	-0.23752400
C	2.41431500	-3.69746700	0.22197200
H	4.31578600	-3.11544300	1.17688900
H	2.43796300	-4.77809600	0.14631400
C	-2.60781400	0.45682400	-2.02657200
C	0.16482700	-3.33395500	-0.84290500
C	-2.05821800	-3.09223000	-1.98385900
C	-2.13914200	-0.84355800	-1.87152000
C	-2.83460200	-2.02779600	-2.32809800
H	-2.24253700	-4.14616600	-2.15548700
H	-3.79241600	-2.02545600	-2.83580200
C	-0.88432900	-2.55561300	-1.32638800
C	-0.48692000	2.93202500	-0.64193100
C	2.84232700	-1.51523300	0.59770900
N	1.61190500	-1.54373400	0.00264500
N	-0.95960400	-1.19131600	-1.27279000
C	-1.68895700	-0.12072400	1.83642700
O	-2.73919200	0.06128300	1.22689100
O	-1.71601400	-0.34535400	3.20792800
C	-3.01379500	-0.35512000	3.80880500
H	-3.64204500	-1.11233400	3.32275500
H	-3.50249700	0.61446200	3.65231700
C	-2.83875700	-0.65087400	5.28942400
H	-3.81478000	-0.66661000	5.78928500
H	-2.36040500	-1.62580000	5.44052300
H	-2.21743200	0.11234200	5.77183100
O	1.37476400	0.17581500	-2.10149300
C	0.81346300	0.37421600	-3.34558700
H	0.22250600	1.30996900	-3.42860700
H	1.60609300	0.44151000	-4.11874100
H	0.13986000	-0.44543700	-3.67100400
H	0.39535100	-0.30648600	2.12064800
H	0.72978100	4.48700700	0.13160900
H	4.48478600	-0.49302000	1.47534100
H	0.07628200	-4.41041200	-0.96242500
H	-3.56246000	0.58819300	-2.52861600

[3]TC⁻ ub3lyp/def2tzvp, e. e. = -2674.545836 a.u.

C	-1.01488000	2.51461400	-1.46208900
N	-0.97010300	1.15129300	-1.37814100
H	-2.41964500	3.95768200	-2.46718300
C	-2.03543800	0.68370300	-2.09334300
C	-2.78737200	1.79003500	-2.64888000
H	-3.67849400	1.68766100	-3.25690600
C	-2.15709200	2.92875400	-2.25137700
C	-2.36690100	-0.65550200	-2.27059800
N	-0.55106700	-1.66285400	-0.96293700
C	-0.22816200	-2.94904400	-0.62041600
C	-1.18946800	-3.87111700	-1.18631500
H	-1.16010500	-4.94745400	-1.06403300
C	-2.08879200	-3.11875100	-1.87868800
H	-2.95449500	-3.44925400	-2.44046800
C	-1.68363700	-1.73758700	-1.72499700
C	-0.08421900	3.39033500	-0.91031500
H	-0.25039700	4.45372500	-1.05961300
H	3.90799600	3.50792900	1.41391400
C	3.00647000	3.17940500	0.91009500
C	2.60647600	1.79606500	0.74761900
H	1.98734100	5.01545100	0.23206100
N	1.42461600	1.72662700	0.06463700
C	1.05814300	3.01345800	-0.20968100
C	2.04243000	3.93647000	0.31706600
C	0.87543100	-3.32899500	0.13823700
H	1.00280000	-4.38918200	0.33910300
H	4.61179600	-1.63280500	2.26501200
C	3.68932800	-1.73355500	1.70516700
C	2.99683000	-2.86925900	1.40914500
H	3.23359200	-3.89256600	1.67554300
C	1.83932900	-2.46056200	0.64404500
N	1.83897700	-1.10113700	0.48098000
C	2.95245600	-0.63169500	1.12468800
C	3.32130300	0.70722300	1.24135600
H	4.24015600	0.92109900	1.78086800
C	-0.48477300	0.14149100	1.27398800
C	-1.86157500	0.01378600	1.66394600
O	-2.84686800	-0.23198600	0.96580200
O	-2.02817600	0.20585700	3.04077100
C	-3.36802700	0.10299000	3.51923700
H	-3.77530900	-0.88973800	3.28622800
H	-4.00691200	0.83548000	3.00838800
H	-3.25221000	-0.87439900	-2.86142700
Fe	0.43554300	0.02234000	-0.46478700
C	-3.34586100	0.34788000	5.01937500
H	-2.95198200	1.34497400	5.24760200
H	-4.35963300	0.27583800	5.43252500
H	-2.71498000	-0.39080300	5.52697300
H	0.16941800	0.34091700	2.12698600
O	1.41839500	0.04782600	-2.07972100
C	1.97985200	-1.06405200	-2.67793600
H	2.56039200	-0.76350200	-3.57324000
H	2.68151300	-1.61723400	-2.02229000
H	1.23016500	-1.80226800	-3.02815100

[5]TC⁻ ub3lyp/def2tzvp, e. e. = -2674.526215 a.u.

C	2.70949600	1.78041900	0.56859800
N	1.48907700	1.73866700	-0.03425700
H	4.06535700	3.50143000	1.12277000
C	1.10207200	3.01540200	-0.31012500
C	2.13795400	3.93053300	0.14278500
H	2.10046800	5.01007400	0.05161700
C	3.13126400	3.16746700	0.68564700
C	-0.09684700	3.38345300	-0.94139700
N	-1.12191700	1.18154000	-1.32729600
C	-2.24262800	0.71727900	-1.94405700
C	-3.00398500	1.84611600	-2.45420500
H	-3.94372900	1.77374000	-2.98952700
C	-2.30772600	2.97342300	-2.12789800
H	-2.56615100	4.00354900	-2.34485400
C	-1.11260800	2.54068400	-1.41986300
C	3.43012200	0.66122100	1.01530800

H	4.38510400	0.86452100	1.49436600
H	3.31140300	-3.96339800	1.46766000
C	3.06888100	-2.93815100	1.21262100
C	1.86548300	-2.50999100	0.52073900
H	4.75907200	-1.74300700	1.97132600
N	1.90269700	-1.15407500	0.36708600
C	3.05183700	-0.68920000	0.93579400
C	3.80155100	-1.81379400	1.46801900
C	-2.59684700	-0.63375400	-2.06690200
H	-3.53381800	-0.84165500	-2.57824200
H	-1.35552900	-4.96819200	-0.94600900
C	-1.37302300	-3.89137600	-1.06911200
C	-2.32647900	-3.13066900	-1.68242900
H	-3.24318100	-3.46459900	-2.15473200
C	-1.89551200	-1.74707500	-1.58163000
N	-0.70080700	-1.70322900	-0.92711600
C	-0.34614100	-2.97883300	-0.59572400
C	0.82985400	-3.34792900	0.07627200
H	0.95646000	-4.41040900	0.27055400
C	-0.36782100	0.16852400	1.28646200
C	-1.70705500	0.05958700	1.79280100
O	-2.75089800	-0.18360800	1.18808300
O	-1.73923800	0.26804000	3.17377300
C	-3.02857700	0.18024200	3.78023700
H	-3.45767900	-0.81535300	3.60606000
H	-3.71040500	0.90588000	3.31804000
H	-0.24811000	4.44951000	-1.09481500
Fe	0.39600700	0.01072900	-0.51092600
C	-2.86258600	0.45351600	5.26592900
H	-2.44593700	1.45280600	5.43673400
H	-3.83373600	0.39428000	5.77309700
H	-2.18953400	-0.27866100	5.72658400
H	0.37369200	0.38614400	2.05634100
O	1.24786200	0.01627800	-2.19159100
C	1.73442300	-1.10854700	-2.83653500
H	2.34312700	-0.80491100	-3.71093300
H	2.38632300	-1.73951100	-2.20343100
H	0.93487300	-1.76846300	-3.22760100

^{1cs}**TS3**$^{-}$ b3lyp/def2tzvp, e. e. = -2674.520740 a.u.
im. frequency -310.61

N	0.80332100	-1.52916500	-0.87241400
Fe	-0.44301800	0.05213000	-0.41726900
C	0.61402400	-0.71469200	0.92737300
C	-0.76129500	-3.38160200	-0.44358300
C	-3.57957800	0.32888500	0.86932300
C	-3.14254300	-0.96192600	0.58414200
C	-3.91649900	-2.16625000	0.79820300
C	-3.11569900	-3.20769300	0.43414200
C	-1.86001700	-2.63064300	-0.00076900
N	-1.90513400	-1.27046500	0.09282100
H	-4.93183000	-2.19470000	1.17602000
H	-3.33873300	-4.26828700	0.45074000
C	1.52035700	-3.64305500	-1.46728800
C	2.01525000	-1.43508800	-1.54944000
C	2.47103700	-2.75900600	-1.88865800
H	1.51415400	-4.72097000	-1.57907500
H	3.39596500	-2.97199800	-2.41157900
C	-3.30476800	2.81316700	1.07690400
C	-1.21252100	2.89291400	0.24327400
C	-2.29740900	3.68133500	0.79093300
H	-4.28205400	3.02607800	1.49437800
H	-2.27498300	4.75707600	0.92036900
C	2.64222000	-0.25412400	-1.90035800
C	-0.01604600	3.42469300	-0.22532500
C	2.21564000	3.27879400	-1.38025700
C	2.19273600	1.03629100	-1.60337200
C	2.94093400	2.24247700	-1.88664500
H	2.45021000	4.33678700	-1.38987500
H	3.90161800	2.27243800	-2.38732900
C	1.01743300	2.69938300	-0.81188800
C	0.45452700	-2.87915800	-0.87215800
C	-2.83302000	1.49226800	0.71381600
N	-1.55392000	1.56257100	0.21801100
N	1.02609000	1.33841500	-0.96327900
C	1.90126100	-0.40309200	1.55110700
O	2.96524900	-0.03237800	1.08423600
O	1.72644400	-0.53048500	2.91731100
C	2.81849200	-0.07887300	3.72873500
H	3.07702000	0.95197100	3.45968200
H	3.70222700	-0.69880500	3.53245200
C	2.38498400	-0.18015100	5.18028400
H	3.20088100	0.14048200	5.83962300
H	1.51484600	0.45680300	5.37375900
H	2.11876500	-1.21127100	5.43922700
O	-1.25318700	0.01717700	-2.18178400
C	-0.90831900	0.87717800	-3.19671100
H	0.13792000	0.77563800	-3.56346300
H	-1.55075700	0.69264400	-4.08615500
H	-1.04067600	1.95596600	-2.95177000
H	0.15215300	-1.57179700	1.41662600
H	-0.88811700	-4.46050600	-0.48527500
H	-4.58514300	0.43724600	1.26813800
H	0.10863600	4.50232100	-0.15450700
H	3.58873300	-0.33867900	-2.42778100

^{1os}**TS3**$^{-}$ ub3lyp/def2tzvp, e. e. = -2674.525110 a.u.
im. frequency -453.25

N	0.85653800	-1.34801000	-1.01710800
Fe	-0.48338900	0.06279000	-0.43420400
C	0.71152200	-0.72199500	0.85552700
C	-0.53818100	-3.36268800	-0.73040000
C	-3.55914200	-0.02275600	1.04649200
C	-3.05082300	-1.24422600	0.59983500
C	-3.72587100	-2.51574900	0.71785500
C	-2.86766800	-3.45682300	0.22219300
C	-1.68026600	-2.75070400	-0.19368100
N	-1.81975400	-1.40988000	0.03275600
H	-4.71767600	-2.65775500	1.13110000
H	-3.01235900	-4.52829100	0.14520100
C	1.74378900	-3.34970300	-1.77505600
C	2.04138100	-1.10501400	-1.71717600
C	2.60506900	-2.36187300	-2.14680600
H	1.82899000	-4.41473000	-1.95617100
H	3.53813600	-2.45771900	-2.68947800
C	-3.43371800	2.43298200	1.51871200
C	-1.41436900	2.76549300	0.57324700
C	-2.51270200	3.40217700	1.26865200
H	-4.39193500	2.52448500	2.01669000
H	-2.55797700	4.45668900	1.51465600

C	2.55046100	0.14254100	-2.01039800
C	-0.30463100	3.44021100	0.08552200
C	1.85956700	3.57724600	-1.19590400
C	2.00858100	1.37011400	-1.61152300
C	2.64260100	2.64820900	-1.82062000
H	2.01053400	4.64779000	-1.12130600
H	3.57437400	2.79952100	-2.35296400
C	0.74376800	2.85792600	-0.62997000
C	0.62188500	-2.72481000	-1.11997700
C	-2.89840500	1.19677200	0.98717800
N	-1.66261400	1.41913800	0.42594300
N	0.84951100	1.52200800	-0.90458000
C	1.99303000	-0.36855000	1.45324200
O	2.96780600	0.19730600	0.98140600
O	1.97091300	-0.75171500	2.78463800
C	3.09185300	-0.32912400	3.57012800
H	3.25489600	0.74621500	3.43308900
H	3.99838100	-0.84115600	3.22239800
C	2.79212500	-0.66489800	5.02052200
H	3.63471800	-0.36967400	5.65780600
H	1.89669600	-0.13663900	5.36635600
H	2.62485000	-1.74041300	5.14877600
O	-1.35018000	0.11616300	-2.12600200
C	-1.15995900	1.10601800	-3.06874200
H	-0.13613600	1.14476900	-3.49563700
H	-1.84102600	0.93850900	-3.92947100
H	-1.38135600	2.12953300	-2.69898000
H	0.30841000	-1.62980400	1.30131200
H	-0.57493500	-4.44060100	-0.86692000
H	-4.54459700	-0.03028700	1.50499600
H	-0.25848800	4.51261200	0.25617500
H	3.48114000	0.17291200	-2.57084800

^{3}**TS3**$^-$ ub3lyp/def2tzvp, e. e. = - 2674.518246 a.u.
im. frequency -551.57

N	-1.07487600	0.95112400	-1.19614800
Fe	0.53949600	-0.00538700	-0.46524100
C	-0.92576400	0.56051500	0.76331500
C	-0.25367700	3.27750600	-1.12258000
C	3.34241700	1.03806600	1.23421400
C	2.58804400	2.02617800	0.59000800
C	2.90816000	3.43287000	0.59182000
C	1.89395400	4.06668900	-0.07368700
C	0.96487700	3.04314700	-0.47234100
N	1.41630000	1.81048100	-0.07897700
H	3.78859800	3.86833200	1.04988200
H	1.77348800	5.12613400	-0.26752000
C	-2.38977800	2.55942300	-2.22635400
C	-2.11207600	0.33461800	-1.90194300
C	-2.95026900	1.34875800	-2.49731600
H	-2.72782300	3.53958600	-2.54120400
H	-3.84045200	1.13851400	-3.07823400
C	3.78746400	-1.29634300	2.03544000
C	2.02836300	-2.25121100	0.99720800
C	3.17183600	-2.49602700	1.85059800
H	4.67872400	-1.07839900	2.61245200
H	3.45448200	-3.46686500	2.24069800
C	-2.26704600	-1.02552000	-2.06667200
C	1.17522600	-3.23828000	0.52598800
C	-0.78324300	-4.05887400	-0.82781000
C	-1.45128800	-2.02534200	-1.52293800
C	-1.73258000	-3.43443100	-1.59006600
H	-0.67386000	-5.11657300	-0.61902200
H	-2.56640000	-3.87612300	-2.12302000
C	0.08138600	-3.02406900	-0.31960500
C	-1.18916800	2.32193200	-1.46351200
C	3.01949600	-0.31151300	1.30037500
N	1.95140400	-0.91462900	0.68554000
N	-0.33224000	-1.79998600	-0.77126600
C	-2.13947600	-0.01166400	1.31059300
O	-2.82453600	-0.94731000	0.91242300
O	-2.48021200	0.65806300	2.48272400
C	-3.58233500	0.11014000	3.21087600
H	-3.43281600	-0.96643300	3.35640800
H	-4.50772500	0.23080900	2.63178900
C	-3.66753000	0.84153200	4.53986000
H	-4.50840200	0.45749500	5.13051300
H	-2.74803200	0.70385900	5.11961600
H	-3.81732800	1.91662400	4.38822000
O	1.50153300	-0.08336400	-2.07305700
C	2.09720100	-1.22452100	-2.58220500
H	1.37881800	-2.02879600	-2.83378700
H	2.63727800	-0.98190600	-3.51885300
H	2.84524200	-1.67210100	-1.89793600
H	-0.75066600	1.56428600	1.14510100
H	-0.48070200	4.30557500	-1.39319700
H	4.23901600	1.36070800	1.75658400
H	1.37420100	-4.26127400	0.83396500
H	-3.12609100	-1.35291500	-2.64653200

^{5}**TS3**$^-$ ub3lyp/def2tzvp, e. e. = -2674.504108 a.u.
im. frequency -319.89

N	1.08342200	-0.90920200	-1.29719900
Fe	-0.54339100	0.08322400	-0.55537900
C	0.84323200	-0.65278400	0.81009200
C	0.10793400	-3.19967500	-1.34002400
C	-3.36799000	-0.97189000	1.18434500
C	-2.67596000	-1.99257200	0.51052700
C	-3.03524100	-3.40380800	0.46362500
C	-2.05438300	-4.03455800	-0.24980700
C	-1.09579800	-3.00842400	-0.63455000
N	-1.51948000	-1.80488700	-0.17353700
H	-3.91386800	-3.84635100	0.91947300
H	-1.97833100	-5.08983500	-0.48697900
C	2.21348100	-2.47177100	-2.53315200
C	2.13101600	-0.27951900	-1.94371500
C	2.85371400	-1.27463400	-2.70211700
H	2.46303100	-3.42599500	-2.98250200
H	3.72455500	-1.06993900	-3.31394500
C	-3.75966300	1.37007500	2.05791300
C	-1.94421200	2.30850600	1.08439400
C	-3.09934800	2.55818400	1.92833900
H	-4.66547300	1.16908400	2.61881500
H	-3.36047900	3.51720400	2.36135300
C	2.40952500	1.09167900	-1.93634000
C	-1.00916200	3.28369700	0.69503200
C	1.08629600	4.14115000	-0.49027700
C	1.68377300	2.11789500	-1.30738900
C	2.04315000	3.52609600	-1.24774500
H	1.02640100	5.18821300	-0.21522600

H	2.91796800	3.97348500	-1.70573700
C	0.13631800	3.10916300	-0.09911700
C	1.07794500	-2.24361300	-1.67075100
C	-3.01247800	0.38452000	1.29528000
N	-1.92948200	0.99019500	0.72205300
N	0.53116800	1.91919000	-0.62210400
C	2.10151100	-0.23929100	1.38152800
O	2.87416600	0.66033900	1.07012100
O	2.35139400	-1.01977200	2.51107700
C	3.45928900	-0.60570400	3.31374200
H	3.38107900	0.46542100	3.53593200
H	4.39574000	-0.75074600	2.75863200
C	3.44221400	-1.43502900	4.58636500
H	4.28610000	-1.15838900	5.23059600
H	2.51364100	-1.27222900	5.14506500
H	3.51975700	-2.50445900	4.35939000
O	-1.47097800	0.33965600	-2.16624900
C	-2.08375000	1.49029100	-2.62236700
H	-1.38688300	2.34306200	-2.75061600
H	-2.54649400	1.31803400	-3.61554800
H	-2.89889800	1.84604000	-1.95952300
H	0.61001000	-1.69126500	1.02544000
H	0.30632400	-4.20870800	-1.69466500
H	-4.27647400	-1.26666300	1.70526400
H	-1.19413200	4.29130400	1.06128900
H	3.30004500	1.39213700	-2.48370800

^{1cs}BC$^-$ b3lyp/def2tzvp, e. e. = -2674.539610 a.u.

N	-0.91795600	1.96205300	0.15218300
Fe	0.38356400	0.02391600	-0.52514800
C	-0.54831000	0.91032800	1.08194300
C	1.09998600	3.37589200	0.36076600
C	3.54957200	-0.83155200	0.14900000
C	3.26021500	0.52637700	0.28452000
C	4.20054300	1.54631300	0.68030400
C	3.49297600	2.71326500	0.74982100
C	2.12459900	2.39906000	0.40345900
N	2.01124200	1.06646500	0.10722100
H	5.25183300	1.38201700	0.88558400
H	3.85050900	3.70034700	1.02014600
C	-1.18291500	4.17398200	-0.37201200
C	-2.13507800	2.10778800	-0.53358600
C	-2.31308300	3.51782700	-0.76862000
H	-0.94386000	5.22066800	-0.51435100
H	-3.16681800	3.93392900	-1.28925800
C	2.96450900	-3.24839900	-0.23029800
C	0.77971500	-2.89890200	-0.67813300
C	1.81713200	-3.90359000	-0.55244600
H	3.95146100	-3.66218300	-0.05910500
H	1.66736300	-4.96623900	-0.70368400
C	-2.90399400	1.09491800	-1.06840200
C	-0.53290500	-3.15293300	-1.05826100
C	-2.89649900	-2.47207600	-1.61655300
C	-2.58596300	-0.27082500	-1.20840400
C	-3.54136300	-1.26836600	-1.63379700
H	-3.29167600	-3.45278300	-1.85397500
H	-4.57712000	-1.06500100	-1.88004000
C	-1.54039700	-2.19998400	-1.20685300
C	-0.24877600	3.20090300	0.12719100
C	2.63841400	-1.83811600	-0.14775600
N	1.29758800	-1.65120400	-0.40441600
N	-1.36865900	-0.85448600	-0.98027700
C	-1.57749900	0.23894800	1.86375800
O	-2.79594400	0.27157000	1.75655600
O	-0.96575500	-0.52533400	2.83435100
C	-1.84148800	-1.29028200	3.66759400
H	-2.43790000	-1.97197000	3.04974700
H	-2.54187600	-0.62234100	4.18485200
C	-0.97891000	-2.05655000	4.65546600
H	-1.61061300	-2.66063900	5.31833100
H	-0.28822500	-2.72657200	4.13152300
H	-0.38723300	-1.37164700	5.27353200
O	0.84685900	0.49205900	-2.30277700
C	0.32549100	-0.14719400	-3.41070100
H	-0.77128800	-0.02777000	-3.54035300
H	0.78647900	0.26386100	-4.33334900
H	0.52485100	-1.23922900	-3.43381200
H	0.27148700	1.23420900	1.71064300
H	1.43378200	4.40840000	0.43782100
H	4.57857900	-1.13769100	0.32205600
H	-0.79638100	-4.18826400	-1.26100300
H	-3.86863500	1.39993300	-1.46749100

^{1os}BC$^-$ ub3lyp/def2tzvp, e. e. = -2674.549237 a.u.

N	-0.37688600	1.96174900	-0.02586700
Fe	0.39809100	-0.00805600	-0.53714900
C	-0.35465700	0.95787100	1.05083000
C	1.82850100	3.04883800	0.34755700
C	3.29655800	-1.59312100	0.23804900
C	3.32644500	-0.19080700	0.32195000
C	4.46367000	0.59489300	0.69917800
C	4.03476200	1.90282200	0.74592500
C	2.64789200	1.89175300	0.39664300
N	2.23437900	0.61220200	0.12155100
H	5.45019100	0.20209400	0.91600100
H	4.60490000	2.78810200	1.00370500
C	-0.27631000	4.26751200	-0.26754800
C	-1.57654100	2.43277000	-0.66554400
C	-1.49587300	3.86558800	-0.72408300
H	0.12554700	5.27378800	-0.26678500
H	-2.27059700	4.48631500	-1.15827300
C	2.17022700	-3.80967500	-0.02712200
C	0.12617400	-3.00738000	-0.55402500
C	0.90958500	-4.20641600	-0.34269000
H	3.03577400	-4.42503600	0.18937500
H	0.52597300	-5.21491600	-0.44465200
C	-2.53636500	1.65980900	-1.24994400
C	-1.18729400	-3.00183200	-0.97903900
C	-3.30575500	-1.84212400	-1.70913100
C	-2.54668100	0.25022300	-1.37676700
C	-3.67266900	-0.51831800	-1.80838900
H	-3.89746800	-2.72310700	-1.92905400
H	-4.62812900	-0.10669300	-2.11190200
C	-1.95450600	-1.85330500	-1.23868400
C	0.48827200	3.10856000	0.09164700
C	2.17609600	-2.36055600	-0.02491200
N	0.91349000	-1.89046200	-0.32843300
N	-1.49943600	-0.57098300	-1.05642600
C	-1.55558000	0.62105900	1.81868000
O	-2.71597600	0.96481000	1.66377600

O	-1.18474800	-0.21602800	2.84206100
C	-2.25351000	-0.69456900	3.66968500
H	-2.98760400	-1.22162500	3.04963200
H	-2.76796900	0.15584200	4.13386000
C	-1.64936800	-1.61462300	4.71490200
H	-2.43670700	-2.00217900	5.37294900
H	-1.14511100	-2.46436700	4.24189700
H	-0.91660000	-1.08209500	5.33171000
O	0.95938300	0.29292600	-2.29088800
C	0.38322500	-0.30450500	-3.40195900
H	-0.64124800	0.05151500	-3.62384800
H	0.99475700	-0.08210000	-4.29883000
H	0.33108300	-1.40812400	-3.32766100
H	0.48880000	1.13146900	1.70882900
H	2.33561300	4.00246900	0.47525400
H	4.22327700	-2.12312200	0.44168600
H	-1.66412200	-3.96581800	-1.13637700
H	-3.39863800	2.18903000	-1.64825400

^{3}**BC**$^{-}$ ub3lyp/def2tzvp, e. e. = -2674.548077 a.u.

N	0.34687600	-1.99290100	-0.00059400
Fe	-0.41910700	-0.01337900	-0.54087100
C	0.37938100	-0.96199800	1.04291500
C	-1.86926300	-3.02520000	0.44824600
C	-3.26980400	1.64591500	0.30826700
C	-3.31488900	0.24144000	0.39869400
C	-4.45643200	-0.52203600	0.80508500
C	-4.05024100	-1.83765200	0.86280900
C	-2.66952100	-1.85511500	0.49391400
N	-2.24104200	-0.58518100	0.19146400
H	-5.43357500	-0.11059700	1.03017300
H	-4.63075400	-2.70789300	1.14674700
C	0.18276100	-4.29128000	-0.25321600
C	1.50622800	-2.48230500	-0.69322100
C	1.38811300	-3.91179400	-0.76317200
H	-0.24601000	-5.28622400	-0.25014800
H	2.12421200	-4.54464600	-1.24448900
C	-2.10660000	3.85221400	0.05819200
C	-0.09522300	3.01119400	-0.53345400
C	-0.84233700	4.22638500	-0.27479700
H	-2.94960500	4.48426300	0.31283000
H	-0.43483800	5.22762300	-0.35389700
C	2.45815700	-1.72149600	-1.31153500
C	1.21245600	2.97331700	-0.98318000
C	3.28398400	1.76608500	-1.77092400
C	2.48974400	-0.31425500	-1.44034800
C	3.61946100	0.43578500	-1.89204300
H	3.88996500	2.63597600	-1.99582500
H	4.55962000	0.00791500	-2.22023900
C	1.94270700	1.80414700	-1.26929800
C	-0.53749400	-3.11565100	0.14951700
C	-2.14677900	2.40319500	0.02409000
N	-0.90731700	1.91644100	-0.32812700
N	1.46676400	0.52972000	-1.08871700
C	1.62164900	-0.63992500	1.75381700
O	2.75371600	-1.06342400	1.58766300
O	1.33429000	0.27946400	2.72934900
C	2.44242300	0.70596900	3.53343200
H	3.17356400	1.22508600	2.90254400
H	2.94323200	-0.16981800	3.96290600
C	1.89710500	1.61989700	4.61598000
H	2.71401800	1.96813800	5.25968400
H	1.40537500	2.49552900	4.17837000
H	1.16640700	1.09405600	5.24096700
O	-1.00745100	-0.29741300	-2.28800900
C	-0.59995500	0.45794700	-3.37759200
H	0.42986700	0.22921400	-3.71390700
H	-1.26388200	0.26021600	-4.24227500
H	-0.64190800	1.54817900	-3.19290400
H	-0.44130100	-1.09796700	1.73828400
H	-2.39123700	-3.96763100	0.59697100
H	-4.18560700	2.18404600	0.53849900
H	1.71751100	3.92363600	-1.13474100
H	3.29652700	-2.26552100	-1.74017400

^{5}**BC**$^{-}$ ub3lyp/def2tzvp, e. e. = -2674.561571 a.u.

N	0.82390300	-1.98562000	0.12127200
Fe	-0.54477400	-0.05528800	-0.79749100
C	0.70810800	-1.00245000	1.13596900
C	-1.29554900	-3.26255700	0.49488400
C	-3.48312400	1.09890300	0.59723800
C	-3.25785100	-0.28476000	0.73156600
C	-4.20859000	-1.24166100	1.26479000
C	-3.59132400	-2.46300000	1.24754400
C	-2.26015100	-2.25093500	0.71589000
N	-2.10036700	-0.93139200	0.40236100
H	-5.20700800	-1.00545000	1.61506900
H	-3.99178200	-3.41593500	1.57458400
C	0.77606600	-4.12863300	-0.67332300
C	1.91974700	-2.15421800	-0.73427200
C	1.91090400	-3.53639500	-1.14799900
H	0.40107600	-5.11602500	-0.91190200
H	2.63670200	-3.96039700	-1.83069700
C	-2.83474100	3.51003200	0.10933600
C	-0.74749300	3.02686200	-0.62740100
C	-1.69713000	4.09694400	-0.36746200
H	-3.75222600	3.99547100	0.42243200
H	-1.50707900	5.15370900	-0.51754400
C	2.75181100	-1.15995200	-1.25362800
C	0.56545100	3.18812300	-1.09946600
C	2.91040700	2.42844400	-1.71883800
C	2.54044400	0.22879400	-1.33953700
C	3.52296300	1.20807000	-1.76411500
H	3.34081500	3.39874000	-1.93896000
H	4.55007400	0.98837000	-2.03247100
C	1.54212600	2.19348500	-1.28784800
C	0.02466700	-3.14605200	0.06736200
C	-2.58796200	2.07776200	0.14314000
N	-1.32615400	1.83073200	-0.31894000
N	1.35660400	0.85602000	-1.07868200
C	1.84415700	-0.46318000	1.80083400
O	3.04523100	-0.59142500	1.54935600
O	1.42816800	0.31248100	2.88012700
C	2.47482300	0.93480700	3.62075200
H	3.06852600	1.58056700	2.96172000
H	3.15737600	0.17442700	4.02309200
C	1.83387000	1.73865800	4.73989100
H	2.60651400	2.23679000	5.33880600
H	1.16351500	2.50508400	4.33514100
H	1.24877000	1.09112300	5.40332400

O	-1.03549500	-0.64842700	-2.53271500
C	-0.77011600	-0.03718200	-3.74280800
H	0.30068900	-0.07689600	-4.03496800
H	-1.32893400	-0.52097200	-4.57144900
H	-1.05615200	1.03575800	-3.76922200
H	-0.20979000	-1.04582400	1.69413200
H	-1.66678500	-4.28376100	0.55630900
H	-4.46022400	1.45440600	0.91707600
H	0.87624900	4.20876700	-1.31179900
H	3.66358700	-1.52911000	-1.71913800

C2H2 b3lyp/def2tzvp, el. energy = 78.628947 a.u.

C	0.66542400	-0.00001500	-0.00004500
H	1.23937600	0.92451900	0.00008100
H	1.23940700	-0.92444100	0.00034400
C	-0.66546200	-0.00003300	-0.00015500
H	-1.23934000	-0.92450900	0.00025700
H	-1.23921200	0.92471700	0.00052000

^{1cs}**TS4**$^{-}$ b3lyp/def2tzvp, e. e. = -2753.159784 a.u.
im. frequency -310.46

C	-0.73763000	2.86304900	-0.76695600
N	-0.89557400	1.50930800	-0.86876400
H	-2.05499000	4.61772100	-1.27813900
C	-2.13339100	1.30141400	-1.40872400
C	-2.78882100	2.57193800	-1.65768600
H	-3.77621500	2.68611400	-2.09009100
C	-1.92601200	3.54188800	-1.24737000
C	-2.70509100	0.05596500	-1.66119300
N	-0.86517900	-1.37323400	-0.88532400
C	-0.68994600	-2.72540700	-0.78978000
C	-1.88165900	-3.41911400	-1.23212800
H	-1.99974900	-4.49628800	-1.25403000
C	-2.76904000	-2.46035500	-1.61857000
H	-3.76702600	-2.58794500	-2.02200900
C	-2.12209000	-1.18238700	-1.39358800
C	0.41447000	3.50460200	-0.31564300
H	0.39593600	4.59106100	-0.28129200
H	4.85924600	2.76117200	0.71395000
C	3.80802200	2.62767500	0.48583900
C	3.15833300	1.34722600	0.28098200
H	2.94773200	4.65720800	0.39266100
N	1.82978900	1.52812000	0.01344200
C	1.61705300	2.87839800	0.01279300
C	2.84695000	3.57980400	0.32810200
C	0.49051700	-3.35087300	-0.39296100
H	0.49528000	-4.43752200	-0.36956500
H	4.92806200	-2.51493800	0.59138700
C	3.87296700	-2.40292900	0.36981800
C	2.93588000	-3.37589800	0.19565400
H	3.06399600	-4.45140900	0.23691700
C	1.68523200	-2.70183100	-0.08878300
N	1.86440100	-1.34288300	-0.06273600
C	3.19104000	-1.13442400	0.20821500
C	3.79910000	0.11132600	0.36015100
H	4.86340700	0.11759500	0.58180600
C	-0.31442600	0.25688600	1.36671600
C	-1.52567500	-0.49163500	1.82458700
O	-1.57853000	-1.63872200	2.23696500
O	-2.64573200	0.28041800	1.71089500
C	-3.89779900	-0.37625500	1.97748300
H	-3.85607600	-0.84534600	2.96673900
H	-4.04979400	-1.17251100	1.24034700
H	-3.70504600	0.04881700	-2.08806000
Fe	0.48037300	0.07436300	-0.39590500
C	-4.99491800	0.67026000	1.90375900
H	-5.02471900	1.14005600	0.91526500
H	-5.96946100	0.20424400	2.09406200
H	-4.83908600	1.45694100	2.65067100
H	-0.37803800	1.30056000	1.68233800
O	1.06368700	0.26498000	-2.23463000
C	0.90903600	-0.70044700	-3.20013200
H	1.50218600	-0.43461700	-4.10289800
H	1.25457700	-1.71495100	-2.90185000
H	-0.13574100	-0.82929200	-3.56436000
C	0.51133500	0.21234300	3.89099400
H	-0.29889800	-0.26493900	4.43543200
H	0.76826100	1.22754900	4.18551700
C	1.10189000	-0.38737300	2.81941800
H	1.97329200	0.05894300	2.35831500
H	0.93175400	-1.43733600	2.61227200

^{1os}**TS4**$^{-}$ ub3lyp/def2tzvp, e. e. = -2753.172532 a.u.
im. frequency -432.83

C	-0.79358900	2.81968100	-0.99711000
N	-0.91208500	1.46018400	-1.05925800
H	-2.15197100	4.51886100	-1.57433500
C	-2.14063600	1.20070300	-1.59841400
C	-2.82866600	2.44259200	-1.89019200
H	-3.81762700	2.51435600	-2.32748600
C	-1.99309100	3.44870700	-1.51163000
C	-2.67112300	-0.06614000	-1.82767300
N	-0.79670600	-1.42331000	-1.00042000
C	-0.58375700	-2.76784100	-0.87566900
C	-1.73537900	-3.50333800	-1.35390200
H	-1.81904600	-4.58364400	-1.36313900
C	-2.64254000	-2.57777500	-1.77078000
H	-3.62648900	-2.74011000	-2.19497700
C	-2.04510500	-1.27780500	-1.54096300
C	0.32284500	3.50912200	-0.53013300
H	0.27574200	4.59478900	-0.53647200
H	4.67967900	2.90772300	0.88736900
C	3.65676500	2.74183600	0.57016400
C	3.05207600	1.43965200	0.36995600
H	2.77090100	4.74567400	0.30924800
N	1.75271600	1.58075400	-0.03016800
C	1.51301400	2.92445700	-0.10295400
C	2.69795300	3.66472800	0.28165400
C	0.58310300	-3.35397700	-0.39238400
H	0.61629500	-4.43910100	-0.34760900
H	4.87108600	-2.35338100	1.00716100
C	3.84152400	-2.28104800	0.67670000
C	2.95838900	-3.28806300	0.42753600
H	3.11350000	-4.35748400	0.50768100
C	1.72383800	-2.66276300	0.00510500
N	1.86072500	-1.29911500	0.01385300
C	3.14344900	-1.03989700	0.41627300
C	3.70535100	0.22583600	0.57622800
H	4.74139900	0.26887900	0.90149800
C	-0.39063100	0.27170900	1.31936400

C	-1.55410600	-0.49489100	1.78382400
O	-1.61803600	-1.68410700	2.06905600
O	-2.67642200	0.30431400	1.88955300
C	-3.89162400	-0.36550800	2.24571200
H	-3.78881300	-0.81197200	3.24291700
H	-4.08111300	-1.18504200	1.54286400
H	-3.66688000	-0.11464600	-2.26042800
Fe	0.47278900	0.07330000	-0.49862500
C	-5.01030600	0.66154400	2.21271900
H	-5.11288700	1.09217200	1.21043500
H	-5.96386000	0.19369600	2.48743900
H	-4.81409500	1.47939800	2.91547300
H	-0.50535700	1.32900800	1.56434400
O	1.14997600	0.18706400	-2.26811400
C	1.41163200	-0.89785000	-3.08277200
H	1.79630100	-0.54855900	-4.06233800
H	2.18098900	-1.58662300	-2.67681200
H	0.52215600	-1.52090800	-3.30617800
C	0.43075400	0.36205100	4.15531800
H	-0.31683700	-0.16479600	4.74470600
H	0.64366300	1.38875200	4.44716200
C	0.96373700	-0.18348900	3.01921500
H	1.79276400	0.30904000	2.52515600
H	0.84803900	-1.24302500	2.81742400

^{3}TS4^{-} ub3lyp/def2tzvp, e. e. = -2753.166514 a.u.
im. frequency -397.00

C	-1.14387100	2.64555400	-1.09186700
N	-1.07957400	1.28066200	-1.12874000
H	-2.70872300	4.13874500	-1.71466400
C	-2.25547300	0.85216500	-1.67620400
C	-3.09884700	1.98608100	-1.99842100
H	-4.08306900	1.91828700	-2.44674600
C	-2.40912900	3.10066200	-1.63158000
C	-2.60593500	-0.47816600	-1.89044300
N	-0.57119100	-1.55219800	-1.02474700
C	-0.18341100	-2.85450300	-0.86391300
C	-1.21938100	-3.74536900	-1.33749900
H	-1.15958000	-4.82712100	-1.32139300
C	-2.23574900	-2.95782300	-1.78511400
H	-3.18508400	-3.25844800	-2.21254900
C	-1.82136800	-1.58658100	-1.57961000
C	-0.13797900	3.48674200	-0.62464700
H	-0.32749300	4.55628000	-0.65577700
H	4.20014900	3.49007200	0.96647000
C	3.22285900	3.18512400	0.61114800
C	2.80565900	1.81238000	0.41590600
H	2.09232600	5.04917800	0.27048900
N	1.51761600	1.77313100	-0.04149100
C	1.10488200	3.07225400	-0.15170700
C	2.16374900	3.96788500	0.26274500
C	1.03725100	-3.27333000	-0.34227000
H	1.20927500	-4.34378000	-0.27245900
H	5.09181800	-1.68822100	1.20293000
C	4.07856900	-1.76078900	0.82572500
C	3.35024500	-2.88028500	0.56085200
H	3.64106300	-3.91802400	0.67346800
C	2.06236300	-2.43039300	0.07612600
N	2.01441900	-1.06247300	0.05877400
C	3.23237600	-0.62911100	0.50783000
C	3.60905900	0.70230600	0.67033100
H	4.61391200	0.89221700	1.03797800
C	-0.41864100	0.20765200	1.32736200
C	-1.58954800	-0.57821200	1.70776900
O	-1.66668900	-1.79447100	1.84970900
O	-2.70221800	0.21529400	1.93935700
C	-3.91048700	-0.48222900	2.25766400
H	-3.80139800	-0.99235300	3.22412000
H	-4.09903000	-1.25656500	1.50503300
H	-3.58120000	-0.67096700	-2.32934000
Fe	0.47078600	0.10307300	-0.54777000
C	-5.03597900	0.53750800	2.29559700
H	-5.14484200	1.03041500	1.32294000
H	-5.98610900	0.04807200	2.54380300
H	-4.84161000	1.31050400	3.04799600
H	-0.52815800	1.25036200	1.63123400
O	1.13566800	0.27411200	-2.29858800
C	1.84484600	-0.70705600	-2.96962800
H	2.01172100	-0.39612700	-4.01968300
H	2.84733500	-0.90460700	-2.54031900
H	1.32059700	-1.68173000	-3.01094200
C	0.35307800	0.08503500	4.25796200
H	-0.38067500	-0.50783300	4.80047400
H	0.53839400	1.08954600	4.63433600
C	0.90138200	-0.35258400	3.08620300
H	1.71251200	0.19949800	2.62573600
H	0.80135500	-1.39013800	2.78659800

^{5}TS4^{-} ub3lyp/def2tzvp, e. e. = -2753.148309 a.u.
im. frequency -381.76

C	-0.44505100	3.01264900	-0.70872300
N	-0.74780000	1.69901100	-0.92599400
H	-1.62791400	4.90936600	-1.02690200
C	-2.02187600	1.62912800	-1.41432500
C	-2.55845900	2.97466600	-1.52526500
H	-3.54897200	3.22263400	-1.88906700
C	-1.58620200	3.82794600	-1.08904600
C	-2.70086700	0.44817300	-1.75294800
N	-0.99111700	-1.23093000	-1.21286300
C	-0.88632200	-2.58735200	-1.27932800
C	-2.12839500	-3.12898500	-1.80275800
H	-2.33114600	-4.18160300	-1.96315700
C	-2.95867800	-2.06999400	-2.03688700
H	-3.96803400	-2.08817200	-2.43162600
C	-2.22731000	-0.87174000	-1.66206000
C	0.77102400	3.49564000	-0.19852300
H	0.85621400	4.57599700	-0.10656400
H	5.02363100	2.24349500	1.20400400
C	3.99334300	2.22774400	0.86723000
C	3.22996200	1.02806300	0.56310900
H	3.39535700	4.34603800	0.74632200
N	1.97458000	1.38963000	0.17358800
C	1.89644500	2.74854800	0.18839100
C	3.16828500	3.29161300	0.63813500
C	0.24244000	-3.33465000	-0.90765000
H	0.16855200	-4.41125200	-1.04293300
H	4.60584200	-3.05657100	0.66662900
C	3.59685500	-2.81527800	0.35228000
C	2.62482500	-3.66953100	-0.07976500
H	2.68456000	-4.74616600	-0.19064300

C	1.45669600	-2.85809900	-0.38895100
N	1.74057500	-1.55321200	-0.12144400
C	3.02933300	-1.47561200	0.31207200
C	3.70945800	-0.29039500	0.63834600
H	4.74006200	-0.40578100	0.96631900
C	-0.34242800	0.10725600	1.33347700
C	-1.54318800	-0.65062000	1.70971300
O	-1.66621100	-1.86572600	1.79390200
O	-2.59436000	0.18011100	2.04132500
C	-3.81823000	-0.47064700	2.40500300
H	-3.65595800	-1.09264500	3.29415100
H	-4.13325600	-1.13837800	1.59450700
H	-3.71386500	0.56964400	-2.12951500
Fe	0.49265200	0.06430400	-0.53066700
C	-4.85332700	0.60965000	2.66718900
H	-5.01444000	1.22017100	1.77168200
H	-5.81067200	0.15637500	2.95273200
H	-4.53205600	1.27326200	3.47823200
H	-0.42116700	1.15055000	1.64276600
O	1.32269700	0.09638700	-2.22872100
C	0.70080300	0.49376200	-3.40038500
H	1.35906000	0.27259000	-4.26398000
H	-0.25569800	-0.02895300	-3.59407100
H	0.48531500	1.57970300	-3.44262400
C	0.35028300	-0.12203300	4.23808600
H	-0.42271100	-0.70416100	4.73558300
H	0.54794300	0.86885100	4.64272500
C	0.93910400	-0.55446600	3.08588700
H	1.78093400	-0.01610000	2.66590000
H	0.81765000	-1.58107900	2.75799100

[1os]INT2⁻ ub3lyp/def2tzvp, e. e. = -2753.205822 a.u.

C	-1.00710000	2.74195900	-0.84272300
N	-1.04185800	1.37839700	-0.91250400
H	-2.47996800	4.35848600	-1.37710500
C	-2.25834200	1.04840600	-1.43820800
C	-3.02635200	2.24693900	-1.71072600
H	-4.02255500	2.26026700	-2.13723700
C	-2.25282100	3.30009500	-1.32851300
C	-2.71976900	-0.24677900	-1.65819300
N	-0.75320200	-1.48422400	-0.85982200
C	-0.46338800	-2.81392300	-0.71499300
C	-1.59321200	-3.61826000	-1.12491400
H	-1.62292600	-4.70104500	-1.10157200
C	-2.55931200	-2.75143200	-1.53703300
H	-3.54733300	-2.97608800	-1.92142600
C	-2.02392700	-1.41739900	-1.36328600
C	0.08388200	3.49951600	-0.42224700
H	-0.03240900	4.57988700	-0.41389800
H	4.56894000	3.16567400	0.63146400
C	3.53552900	2.93725200	0.39857200
C	3.00177800	1.60155100	0.22735200
H	2.50354600	4.88206300	0.25448100
N	1.66498100	1.66203400	-0.05523900
C	1.33554800	2.98880200	-0.08777300
C	2.49786400	3.79924700	0.21236100
C	0.75981000	-3.32763900	-0.29316200
H	0.85263400	-4.40812200	-0.22777200
H	5.08723100	-2.06879400	0.71795600
C	4.03124600	-2.05967600	0.47468400
C	3.18617900	-3.11781400	0.32695100
H	3.40565800	-4.17493100	0.41793200
C	1.88855300	-2.56829000	0.00042300
N	1.94823300	-1.19967900	-0.02807600
C	3.24621500	-0.86439200	0.25334900
C	3.74720900	0.43179400	0.35712900
H	4.80324300	0.53811100	0.59018300
C	-0.20015100	0.21189200	1.62350500
C	-1.44553500	-0.52742500	1.88353400
O	-1.55339200	-1.68937700	2.26247900
O	-2.57078600	0.24232800	1.67809800
C	-3.82579700	-0.41263600	1.87993400
H	-3.82474300	-0.92285800	2.85003500
H	-3.97063800	-1.18111700	1.11067000
H	-3.71902700	-0.35505600	-2.07143000
Fe	0.45097700	0.08514800	-0.46338700
C	-4.91758800	0.64233800	1.81370600
H	-4.90734600	1.15750900	0.84717800
H	-5.90220500	0.17681800	1.94774800
H	-4.78432500	1.39468600	2.59971800
H	-0.39434200	1.28465300	1.67051900
O	0.98442700	0.23056400	-2.26754600
C	1.35069800	-0.84397600	-3.06002000
H	1.65254500	-0.48082200	-4.06204400
H	2.21100900	-1.41797600	-2.66324400
H	0.53335600	-1.57309500	-3.22715200
C	0.53768600	0.23334200	4.03344700
H	-0.19878400	-0.33120700	4.60175900
H	0.72884300	1.25373800	4.36383900
C	0.89877800	-0.18749800	2.64596600
H	1.84276700	0.27868100	2.35286300
H	1.03627400	-1.27308700	2.60513000

[3]INT2⁻ ub3lyp/def2tzvp, e. e. = -2753.206292 a.u.

C	-0.88313400	2.81381300	-0.79036300
N	-0.98167200	1.45447200	-0.89114400
H	-2.28099800	4.50849200	-1.27933400
C	-2.21363700	1.19405600	-1.42060200
C	-2.92604800	2.43305600	-1.66089200
H	-3.92218700	2.50264200	-2.08198500
C	-2.10354700	3.43979000	-1.25669300
C	-2.73448400	-0.07208300	-1.67305800
N	-0.83554800	-1.41755300	-0.88764400
C	-0.61377500	-2.76278900	-0.75863400
C	-1.77851700	-3.50348300	-1.18902700
H	-1.86172600	-4.58373600	-1.18145100
C	-2.69633700	-2.58371500	-1.59845600
H	-3.69056500	-2.75366600	-1.99477100
C	-2.09761900	-1.28065700	-1.39933600
C	0.24463500	3.51079000	-0.36357700
H	0.17903700	4.59502900	-0.33431300
H	4.72081800	2.93929100	0.62779400
C	3.67441400	2.76647200	0.40533600
C	3.07138600	1.46149200	0.22449400
H	2.74010800	4.76263300	0.30030400
N	1.73629300	1.59377300	-0.04059700
C	1.47407300	2.93477700	-0.05165200
C	2.67931100	3.68212200	0.24415000
C	0.57766600	-3.34249200	-0.33269200
H	0.61455600	-4.42679700	-0.27692600

H	4.96599200	-2.31768500	0.68361600
C	3.91101500	-2.25147000	0.44484300
C	3.01128300	-3.26322500	0.29271200
H	3.17557400	-4.33102800	0.37517400
C	1.74327300	-2.64479100	-0.02736200
N	1.87454800	-1.28149200	-0.04515800
C	3.18844500	-1.01543000	0.23356400
C	3.75557700	0.25330600	0.33886200
H	4.81747600	0.30369700	0.56399200
C	-0.17912900	0.21590000	1.64142000
C	-1.42755900	-0.52016800	1.90319600
O	-1.53467200	-1.67640300	2.29915100
O	-2.55181500	0.24324500	1.67547800
C	-3.80703200	-0.40819100	1.89014900
H	-3.80035300	-0.90927300	2.86483100
H	-3.95763900	-1.18363500	1.12905100
H	-3.73586100	-0.12281000	-2.09214000
Fe	0.44613800	0.08341000	-0.46811300
C	-4.89803200	0.64727300	1.82137300
H	-4.89587300	1.15222800	0.84951900
H	-5.88183800	0.18401600	1.96842500
H	-4.75745200	1.40756900	2.59838800
H	-0.36730900	1.28979100	1.68015100
O	1.01064500	0.23117800	-2.26543000
C	1.33368300	-0.84852900	-3.06929400
H	1.61748500	-0.49046200	-4.07858700
H	2.19292300	-1.44028000	-2.69610500
H	0.49837200	-1.56179300	-3.21550400
C	0.54555000	0.24874100	4.03332900
H	-0.19648200	-0.31218700	4.59769700
H	0.71536800	1.27882500	4.34516200
C	0.92184800	-0.18650000	2.65416600
H	1.86733100	0.27605500	2.36060800
H	1.05519000	-1.27252100	2.61980800

^{1os}TS5$^-$ ub3lyp/def2tzvp, e. e. = -2753.197051 a.u.
im. frequency -906.74

C	-0.95582400	2.72527100	-0.88741300
N	-0.98722900	1.36066700	-0.93829300
H	-2.42738600	4.33546300	-1.44770900
C	-2.20008300	1.02563200	-1.47340800
C	-2.96893600	2.21978900	-1.76336500
H	-3.96173000	2.22777900	-2.19815100
C	-2.19899500	3.27781100	-1.38641400
C	-2.65588300	-0.27258500	-1.68687900
N	-0.70351900	-1.49979200	-0.84296800
C	-0.41506400	-2.82792600	-0.68891900
C	-1.53652900	-3.63895900	-1.11190400
H	-1.56421700	-4.72183500	-1.08264000
C	-2.49791800	-2.77734700	-1.54606000
H	-3.47864500	-3.00672700	-1.94620900
C	-1.96623400	-1.44102400	-1.37140800
C	0.13393800	3.48605000	-0.46373700
H	0.01708200	4.56675800	-0.46472900
H	4.58094200	3.17146500	0.74327000
C	3.55875600	2.93736000	0.46937400
C	3.03808000	1.59919100	0.28091400
H	2.52615800	4.87817700	0.28166300
N	1.71236000	1.65036200	-0.05604000
C	1.37803900	2.98101400	-0.09991600
C	2.52520800	3.79512800	0.24070000
C	0.80545800	-3.33307800	-0.24215300
H	0.89862400	-4.41279900	-0.16059000
H	5.09872500	-2.07056100	0.89912400
C	4.05338500	-2.06094400	0.61287000
C	3.20906900	-3.11799200	0.45266500
H	3.42087900	-4.17393300	0.57296600
C	1.92695400	-2.57118500	0.06583300
N	1.99586700	-1.19874300	0.00654500
C	3.28383900	-0.86612000	0.33813500
C	3.78065800	0.43138000	0.44978200
H	4.82654300	0.54150700	0.72403200
C	-0.34072800	0.24766700	1.79690500
C	-1.59391700	-0.48914300	1.96988200
O	-1.70832800	-1.64010900	2.37823000
O	-2.69021600	0.26484400	1.64191100
C	-3.95921400	-0.38987400	1.75375800
H	-4.02257900	-0.90281900	2.71989600
H	-4.04594900	-1.15295500	0.97127900
H	-3.64876600	-0.38487000	-2.11515800
Fe	0.51815200	0.07575000	-0.50319000
C	-5.04063500	0.66791800	1.61701900
H	-4.96225500	1.18825400	0.65655900
H	-6.03198700	0.20227300	1.68018300
H	-4.96115000	1.41465200	2.41551100
H	-0.44977500	1.31575400	1.65096000
O	1.00287800	0.19802700	-2.33681000
C	1.36746000	-0.89965000	-3.09262000
H	1.70080100	-0.56516200	-4.09622200
H	2.20974100	-1.48268600	-2.66667300
H	0.54349300	-1.62104200	-3.26990100
C	0.25038900	0.41917000	3.89992200
H	-0.52493600	-0.09211700	4.46081600
H	0.39495300	1.47805800	4.09572400
C	0.81526300	-0.19705100	2.68466300
H	1.76585800	0.22968700	2.37052600
H	0.88739400	-1.28440700	2.72769800

^{3}TS5$^-$ ub3lyp/def2tzvp, e. e. = -2753.204224 a.u.
im. frequency -534.58

C	-0.70044900	2.88038200	-0.80564200
N	-0.88212700	1.52858900	-0.91535700
H	-1.99611000	4.66156900	-1.26896800
C	-2.13485500	1.34994700	-1.43374600
C	-2.77104900	2.63236400	-1.65807000
H	-3.76521800	2.76757800	-2.06795000
C	-1.88419600	3.58377600	-1.25532300
C	-2.73405000	0.11941400	-1.68995200
N	-0.92319400	-1.34430000	-0.91044700
C	-0.78138600	-2.70270400	-0.79811400
C	-1.98646200	-3.36900100	-1.24107200
H	-2.13272000	-4.44257200	-1.24765700
C	-2.84837200	-2.39170000	-1.63952800
H	-3.84907000	-2.49857100	-2.04178700
C	-2.17422900	-1.12857800	-1.42441100
C	0.47036000	3.50254000	-0.37789000
H	0.47243700	4.58867200	-0.34141000
H	4.88024800	2.65020400	0.71008400
C	3.83199700	2.54293900	0.45656600
C	3.15456700	1.27878300	0.25318000

H	3.02753400	4.59426000	0.33548100
N	1.83745100	1.49401200	-0.05258500
C	1.65971500	2.85043000	-0.05860900
C	2.90081300	3.51990800	0.27118300
C	0.37021000	-3.35612000	-0.36769100
H	0.34013200	-4.44125900	-0.32188500
H	4.78416500	-2.61724800	0.77747500
C	3.74363700	-2.48253000	0.50603100
C	2.78655500	-3.43451900	0.32120700
H	2.87939600	-4.51085100	0.40561300
C	1.57088700	-2.73495700	-0.03483700
N	1.78925800	-1.38176600	-0.04632900
C	3.10928700	-1.20202600	0.27497300
C	3.75374300	0.02837800	0.39514000
H	4.80924800	0.01054500	0.65364000
C	-0.20237400	0.29176800	1.90186300
C	-1.44759400	-0.46061800	2.06735800
O	-1.54537400	-1.61410600	2.47299200
O	-2.55167700	0.27794500	1.73183100
C	-3.81406500	-0.38867500	1.85368000
H	-3.86368500	-0.90217500	2.82026000
H	-3.90077800	-1.15169100	1.07142100
H	-3.73711200	0.13306800	-2.10837800
Fe	0.47245600	0.06949000	-0.54805000
C	-4.90690600	0.65861600	1.72737800
H	-4.84771000	1.17462300	0.76335400
H	-5.89281300	0.18386800	1.80609300
H	-4.82357900	1.40968900	2.52138300
H	-0.31681400	1.35450300	1.72632100
O	1.06019400	0.16448300	-2.40575900
C	1.34090600	-0.95037000	-3.16547800
H	1.61206200	-0.65982800	-4.20365900
H	2.19685200	-1.55192600	-2.78902500
H	0.48955600	-1.65801500	-3.26478700
C	0.38274100	0.46643800	4.01828200
H	-0.37086300	-0.06990700	4.58546800
H	0.49061900	1.53066700	4.20984000
C	0.94961400	-0.13048700	2.78580500
H	1.89531600	0.31178400	2.47440800
H	1.04591600	-1.21642200	2.83079200

^{5}FP-2$^-$ ub3lyp/def2tzvp, e. e. = -5168.932869 a.u.

N	3.68769300	-2.12893400	0.57359500
Fe	4.51672000	-0.12563500	0.51200500
C	4.62183100	-2.97795100	-1.55812300
C	5.07782500	1.83003500	-2.38381600
C	5.13004000	0.42402100	-2.54130300
C	5.67778800	-0.25500200	-3.70224700
C	5.55533000	-1.59209400	-3.47207300
C	4.92721400	-1.73869100	-2.17084700
N	4.69684100	-0.50126300	-1.62979500
H	6.11198500	0.22333500	-4.56903700
H	5.87025700	-2.39968200	-4.11804100
C	3.59170200	-4.41312400	0.26589800
C	3.07146600	-2.70430900	1.65180600
C	3.01160400	-4.14464500	1.46849700
H	3.70560600	-5.37947300	-0.20511700
H	2.57707300	-4.85235200	2.16149200
C	4.49934900	3.95720600	-1.12173500
C	3.79806500	2.93126900	0.76445200
C	4.01527600	4.21705300	0.12418700
H	4.75360400	4.67543100	-1.88857800
H	3.80762200	5.18395300	0.56257200
C	2.53357900	-2.01671500	2.76767600
C	3.24975500	2.76763700	2.05853700
C	2.20480900	1.39959900	3.92116300
C	2.55316900	-0.61667600	2.95805500
C	1.97995100	0.07013000	4.10578900
H	1.91803700	2.21391100	4.57274200
H	1.47476600	-0.40278100	4.93654900
C	2.89561600	1.53493700	2.64887000
C	4.01563700	-3.13985900	-0.29174700
C	4.60366600	2.51147700	-1.24075900
N	4.16127400	1.92417200	-0.08547000
N	3.09533900	0.29881200	2.09784700
O	6.24274800	-0.15314400	1.29552200
C	7.27021400	-1.06238000	1.15437400
H	7.67975800	-1.11262200	0.12265300
H	8.13044400	-0.80704700	1.80868300
H	6.98706600	-2.10286500	1.42330000
C	1.87772700	-2.84725100	3.83823200
C	2.54454300	-3.08288300	5.04643900
C	0.58368200	-3.40417400	3.66105500
C	1.97162100	-3.85021400	6.06205900
H	3.53557300	-2.65757800	5.17825600
C	0.00583800	-4.17630300	4.68194900
C	0.70247000	-4.39528700	5.86997900
H	2.51448100	-4.02075100	6.98795000
H	-0.97929200	-4.59313900	4.52733000
H	0.24030000	-4.99641700	6.64931500
C	4.93862600	-4.22509900	-2.32790100
C	4.25624100	-4.53671600	-3.51611300
C	5.92610300	-5.11700400	-1.87575800
C	4.55128200	-5.69985800	-4.22972100
H	3.48361700	-3.86216200	-3.87492400
C	6.22275100	-6.28014500	-2.58901900
H	6.46730100	-4.88753900	-0.96197800
C	5.53632600	-6.57624900	-3.76883700
H	4.00615700	-5.92299600	-5.14381700
H	6.99451800	-6.95300900	-2.22307500
H	5.76606400	-7.48239400	-4.32399900
C	5.56164000	2.66887200	-3.52827900
C	6.71038900	3.46845100	-3.39996700
C	4.87839700	2.68124200	-4.75636100
C	7.16010300	4.25412800	-4.46279700
H	7.25445500	3.46349700	-2.45961700
C	5.32731600	3.46626700	-5.82014200
H	3.98379800	2.07510600	-4.87011900
C	6.47049500	4.25602600	-5.67750400
H	8.05406700	4.86108400	-4.34148900
H	4.77973300	3.46358600	-6.75959200
H	6.82037100	4.86793900	-6.50524500
C	3.05704900	4.01209700	2.88550000
C	1.87421000	4.79002900	2.82626900
C	4.08419100	4.42083100	3.74465600
C	1.75456800	5.94477800	3.61870500
C	3.96776800	5.56692900	4.53209000
H	4.98836400	3.81991000	3.78623400
C	2.79850700	6.32365800	4.46144800
H	0.84336000	6.52234600	3.56577300

H	4.78112900	5.86138200	5.18981400
H	2.68767300	7.21989300	5.06713000
N	0.85751100	4.35662400	1.95071500
N	-0.06750500	-3.15099600	2.43696300
C	-0.39695500	4.88140400	1.75641700
O	-0.83287700	5.86185600	2.36179900
C	-1.22209600	-3.69659700	1.93131700
O	-1.96568000	-4.43991100	2.57326800
C	-1.53691400	-3.32985000	0.50382500
C	-2.84034300	-3.58535100	0.05508000
C	-0.59256600	-2.81414800	-0.39807500
C	-3.22070000	-3.32654700	-1.26458600
H	-3.55049500	-4.00230700	0.76248900
C	-0.96366800	-2.54630500	-1.71428900
H	0.43985800	-2.64464200	-0.10909300
C	-2.26665300	-2.79738800	-2.14273700
H	-0.22764500	-2.14918400	-2.40773400
H	-2.54550000	-2.58815000	-3.17258000
C	-1.24727200	4.15661100	0.74955300
C	-2.63345100	4.28518100	0.89147500
C	-0.73601000	3.39380000	-0.31149300
C	-3.52491200	3.65451600	0.01851700
H	-3.00747500	4.89095900	1.71201400
C	-1.61642900	2.77081500	-1.19493300
H	0.33450700	3.30513900	-0.47509200
C	-2.99841600	2.89430900	-1.03174900
H	-1.22245700	2.17992700	-2.01717800
H	-3.66547800	2.38461000	-1.72012300
H	0.42564800	-2.49855500	1.84156200
H	1.06668500	3.48576500	1.47689600
C	-5.01851500	3.86117700	0.25257500
H	-5.25993600	4.90791500	0.02430900
H	-5.20123700	3.75075200	1.32952300
C	-5.99510200	2.98856500	-0.50837900
C	-6.04079400	1.57611000	-0.29320600
C	-6.89049600	3.54435300	-1.39651600
C	-6.94078800	0.75630000	-0.96099000
C	-7.85253600	2.76132100	-2.08533300
H	-6.87170800	4.61765900	-1.57484300
C	-7.88224700	1.34423400	-1.87039900
C	-8.84957700	0.57726000	-2.58047600
C	-9.73837700	1.17776600	-3.44644500
H	-8.88466300	-0.49648300	-2.42869200
H	-10.46997600	0.57151600	-3.97437100
C	-9.70765700	2.57769800	-3.65342900
C	-8.78214100	3.34852700	-2.98617200
H	-10.41322900	3.03948500	-4.33904000
H	-8.74699700	4.42483600	-3.13947900
C	-6.89020800	-0.72460200	-0.73387200
C	-7.82625700	-1.37598500	0.13761700
C	-5.89646400	-1.47706800	-1.34341900
C	-7.70464700	-2.78612600	0.36299400
C	-5.75081100	-2.87834400	-1.10611100
C	-6.65141800	-3.49822500	-0.26741200
H	-6.55997500	-4.56554400	-0.07657800
C	-4.62374600	-3.65994700	-1.75774100
H	-4.81564400	-4.72650900	-1.58681500
H	-4.64946600	-3.51489200	-2.84369200
C	-8.87499300	-0.67671600	0.79970000
C	-9.75715100	-1.33596900	1.62890900
H	-8.97483300	0.39244600	0.64385500
H	-10.55023700	-0.78082200	2.12312100
C	-8.63088700	-3.43523000	1.22372200
C	-9.63778500	-2.72981900	1.84388800
H	-8.52468600	-4.50514200	1.38806700
H	-10.33787500	-3.23835200	2.50144500
O	-4.97398000	-0.86675000	-2.16637700
O	-5.10534800	1.03148400	0.55442100
C	-5.35062400	-0.76422600	-3.54053100
H	-5.52053700	-1.75117900	-3.99238400
H	-6.25505000	-0.15856700	-3.66761800
H	-4.51698700	-0.27606000	-4.05192100
C	-5.50951300	0.86148400	1.91511000
H	-5.83097600	1.81002400	2.36593400
H	-6.32208700	0.13208000	2.00588800
H	-4.63080500	0.49183100	2.44849100

^{5}FP-2$^-$-EDA ub3lyp/def2tzvp, e. e. = -5585.089127 a.u.

N	-3.08264100	2.40901000	1.18137400
Fe	-4.31956000	0.63712500	1.10608600
C	-2.98301600	-3.49416600	-3.45902000
C	-4.38290800	3.83780400	-0.37172700
C	-6.17142100	-0.53822500	-1.66347700
C	-5.93330000	0.85402800	-1.59601900
C	-6.62537600	1.84492700	-2.39912700
C	-6.13210600	3.06192000	-2.03820000
C	-5.12882400	2.82434900	-1.01688200
N	-5.04379700	1.47963900	-0.76247000
H	-7.40049000	1.64291200	-3.12485000
H	-6.43386600	4.02767300	-2.41799900
C	-2.61267600	4.66486400	1.24801700
C	-2.09774400	2.63706100	2.10537800
C	-1.80395900	4.05790300	2.16165500
H	-2.65164500	5.71842700	1.01023200
H	-1.07774700	4.53003000	2.80908400
C	-5.74687000	-2.94773300	-0.98943700
C	-4.29918100	-2.48045800	0.68234300
C	-4.97714300	-3.54377000	-0.03753300
H	-6.37190000	-3.43588600	-1.72384300
H	-4.85383700	-4.60302000	0.13917600
C	-1.43858100	1.65340200	2.87954400
C	-3.35320100	-2.69524300	1.71183500
C	-1.54883700	-1.93580600	3.32692600
C	-1.69890700	0.26668200	2.83290800
C	-1.00105400	-0.72911000	3.63069100
H	-1.27003000	-2.90017700	3.72796600
H	-0.19843100	-0.53115100	4.32694800
C	-2.56936100	-1.69215300	2.32106300
C	-3.42054300	3.62262700	0.63962300
C	-5.55706300	-1.51377300	-0.84928700
N	-4.67007600	-1.26933700	0.16569200
N	-2.63560500	-0.35022400	2.04674700
C	-2.23377900	-2.94458900	-2.33358500
O	-1.75631900	-3.62920600	-1.43476900
O	-2.13904700	-1.61758100	-2.45294000
C	-1.47568000	-0.86580700	-1.39453600
H	-2.10371100	0.01441400	-1.24626300
O	-5.65850300	0.82145800	2.43642200
C	-6.42446800	1.91207800	2.77707000

H	-7.02288200	2.32259600	1.93353800
H	-7.16049300	1.66477500	3.57273700
H	-5.83244300	2.76807200	3.17289600
H	-3.44953100	-2.90384900	-4.23543500
C	-0.41011600	2.11844200	3.87468600
C	-0.72236400	2.08190900	5.24003900
C	0.87896600	2.57345600	3.49033000
C	0.19418700	2.47349700	6.21586100
H	-1.71097700	1.73633500	5.52931300
C	1.80386300	2.96385200	4.47336900
C	1.45754200	2.91242200	5.82270900
H	-0.07862200	2.43394900	7.26691700
H	2.78536800	3.29346600	4.16463300
H	2.18839600	3.21913600	6.56694800
C	-4.62070300	5.25375000	-0.80330200
C	-4.28451500	5.68654800	-2.09760700
C	-5.18595000	6.18834700	0.08166000
C	-4.50568200	7.00679900	-2.49371500
H	-3.83971700	4.98043600	-2.79342500
C	-5.40693000	7.50890300	-0.31294500
H	-5.46066300	5.86846700	1.08292800
C	-5.06741300	7.92390600	-1.60263900
H	-4.23280800	7.31933200	-3.49878200
H	-5.85019400	8.21187400	0.38803900
H	-5.23947600	8.95228200	-1.91038500
C	-7.14034300	-1.02107200	-2.69912600
C	-8.35135000	-1.63183700	-2.33009400
C	-6.85889300	-0.88206400	-4.06961700
C	-9.24934500	-2.08751900	-3.29704200
H	-8.58908400	-1.73802900	-1.27529900
C	-7.75638900	-1.33683300	-5.03730500
H	-5.92367600	-0.41894600	-4.37285000
C	-8.95569400	-1.94245900	-4.65500500
H	-10.18247900	-2.55147500	-2.98709100
H	-7.51491100	-1.22162700	-6.09129800
H	-9.65527200	-2.29663700	-5.40793900
C	-3.23231000	-4.09862600	2.23344400
C	-2.08727500	-4.90690900	2.07176700
C	-4.32520600	-4.63420200	2.94065900
C	-2.05553900	-6.19510900	2.62955600
C	-4.28923300	-5.91129100	3.49352600
H	-5.20753700	-4.01381200	3.06899800
C	-3.14230700	-6.69349100	3.33977300
H	-1.16619700	-6.79921700	2.49629500
H	-5.14655200	-6.28834100	4.04489100
H	-3.09505400	-7.69310700	3.76406400
H	-1.47980800	-1.45851500	-0.48089600
N	-0.96406500	-4.45053700	1.32874800
N	1.17355900	2.64375000	2.11116500
C	0.32538600	-4.55041500	1.79549900
O	0.59364800	-4.94888100	2.93012900
C	2.34567300	3.00253200	1.49232600
O	3.40598500	3.19672800	2.09140100
C	2.27002200	3.15431900	-0.00387200
C	3.47752600	3.06424300	-0.71396400
C	1.08574000	3.44611100	-0.69345800
C	3.51979700	3.22637200	-2.09870500
H	4.38345000	2.86532400	-0.15352200
C	1.11808900	3.61675300	-2.07919800
H	0.14609800	3.58411300	-0.16704700
C	2.31966100	3.49952500	-2.77442900
H	0.20018300	3.84825900	-2.61280000
H	2.33119500	3.62924200	-3.85494200
C	1.42588800	-4.15529100	0.84847900
C	2.65637000	-3.79883200	1.41271300
C	1.30163800	-4.21462400	-0.54715700
C	3.76662700	-3.48911000	0.62009400
H	2.73667800	-3.78191200	2.49577000
C	2.40202200	-3.90590700	-1.34630600
H	0.36332800	-4.50604700	-1.00516800
C	3.62361200	-3.54570700	-0.77176000
H	2.31172400	-3.95625500	-2.42867900
H	4.46866200	-3.31172400	-1.41187400
H	0.40597100	2.37682100	1.50794900
H	-1.15211500	-3.94561700	0.46305000
C	-0.06710700	-0.49945500	-1.82313600
H	-0.07191400	0.07882500	-2.75369700
H	0.40199300	0.11222100	-1.04510600
H	0.55048300	-1.39305300	-1.96397300
C	5.09074700	-3.16494400	1.31337500
H	5.45820900	-4.08345400	1.79082600
H	4.88282200	-2.46288700	2.12674400
C	6.19233600	-2.62281700	0.42839100
C	6.24931000	-1.23115800	0.10881100
C	7.14625200	-3.46134600	-0.10761400
C	7.21668400	-0.71169100	-0.74554100
C	8.16387100	-2.98627000	-0.97361700
H	7.12220200	-4.52267800	0.13204100
C	8.19578000	-1.59386000	-1.31464400
C	9.20013300	-1.14976400	-2.22196200
C	10.12723800	-2.02624500	-2.74373700
H	9.23199400	-0.10235900	-2.50212500
H	10.88473200	-1.66174000	-3.43285500
C	10.10245000	-3.39683200	-2.39043300
C	9.13792600	-3.86250700	-1.52551900
H	10.84111600	-4.07708400	-2.80612400
H	9.10195600	-4.91485700	-1.25260600
C	7.18982300	0.74775100	-1.08129800
C	8.17584900	1.65500700	-0.56354900
C	6.15926900	1.24938500	-1.86552400
C	8.07762000	3.05028800	-0.88025400
C	6.02524500	2.64344700	-2.14327400
C	6.98309100	3.50663300	-1.65895500
H	6.89764400	4.57241500	-1.86123100
C	4.81919200	3.15352000	-2.90064700
H	5.04908300	4.15775000	-3.28122200
H	4.63768300	2.52936700	-3.78340100
C	9.24915900	1.23211500	0.27130400
C	10.18001600	2.13123000	0.74562300
H	9.32877000	0.18361100	0.53712100
H	10.98909100	1.78288800	1.38241700
C	9.05677700	3.95015400	-0.37857400
C	10.08994000	3.50440000	0.41453900
H	8.96724600	5.00505400	-0.62797100
H	10.83054200	4.20314900	0.79439100
O	5.17951600	0.41617300	-2.35663100
O	5.27301900	-0.41890100	0.63314000
C	5.50267100	-0.25923900	-3.57202100
H	5.70478800	0.44963800	-4.38764900
H	6.37109800	-0.91706500	-3.45236000

H	4.62742300	-0.86108800	-3.82965500
C	5.63404800	0.29606200	1.82083300
H	5.83435100	-0.39094300	2.65486000
H	6.51679500	0.92435400	1.65803400
H	4.78374800	0.93359600	2.07089800
N	-3.19242700	-4.78214300	-3.47682900
N	-3.36106100	-5.90703800	-3.48182900

[1os]**INT1-2⁻** ub3lyp/def2tzvp, e. e. = - 5585.073879 a.u.

N	-3.62704700	2.16702200	0.50402400
Fe	-3.96791300	0.17294600	0.34833200
C	-2.09577000	-0.00051300	-0.83989700
C	-4.68643700	2.95962500	-1.58784300
C	-5.64985100	-1.81831500	-1.87244400
C	-5.60795400	-0.44042300	-2.12917400
C	-6.26404500	0.18578700	-3.25420900
C	-5.97695900	1.51291900	-3.19114900
C	-5.15183700	1.70714700	-2.01980300
N	-4.94933900	0.50671700	-1.38495700
H	-6.86518200	-0.32940300	-3.98967400
H	-6.30439900	2.29456800	-3.86139600
C	-3.55014100	4.44065900	0.10111500
C	-2.99080200	2.79926000	1.54346000
C	-2.95375000	4.22506000	1.30439400
H	-3.69214000	5.38354600	-0.40647700
H	-2.52321600	4.95892000	1.97110000
C	-4.98187700	-3.87109800	-0.58968600
C	-3.78948600	-2.80229800	0.99285500
C	-4.23773800	-4.09676800	0.52207100
H	-5.47648900	-4.59764000	-1.21817700
H	-4.00711000	-5.04382700	0.98866500
C	-2.42333000	2.18884500	2.67475200
C	-3.04015300	-2.61808900	2.16253200
C	-1.95292600	-1.16430300	3.89645100
C	-2.40795400	0.80337400	2.89350400
C	-1.78880300	0.17579600	4.04405100
H	-1.62444900	-1.95344400	4.55776600
H	-1.29513800	0.69950800	4.84999200
C	-2.67063000	-1.36207900	2.65590400
C	-3.98755200	3.15189900	-0.38674800
C	-4.99050100	-2.43854800	-0.80451100
N	-4.24682900	-1.81446400	0.16338300
N	-2.92979100	-0.15360200	2.06259100
C	-0.99050100	-0.53473200	-0.03309700
O	-0.72656000	-1.71887500	0.14028600
O	-0.22882700	0.46058700	0.51377100
C	0.94606000	0.04305300	1.24831800
H	1.13217300	0.85184500	1.95969400
O	-5.51421700	0.24346800	1.36207100
C	-6.49538700	1.21514200	1.20641300
H	-6.91236800	1.26018300	0.18451800
H	-7.33636000	0.98613700	1.88616900
H	-6.14913300	2.23397700	1.46067200
H	-1.95699600	1.04197700	-1.10863900
C	-1.82117500	3.07153900	3.73261000
C	-2.53069900	3.27846300	4.92297200
C	-0.55601800	3.69705300	3.58064900
C	-2.02713900	4.07569600	5.95076500
H	-3.49968600	2.79864700	5.02978700
C	-0.04685500	4.49658000	4.61901000
C	-0.78151400	4.68102200	5.78898800
H	-2.60148500	4.21973100	6.86192700
H	0.92174100	4.95851200	4.49452600
H	-0.36735900	5.30431800	6.57779900
C	-4.97744600	4.15429900	-2.44479900
C	-4.36478400	4.30092700	-3.70087400
C	-5.86794100	5.15435700	-2.01855600
C	-4.63077200	5.41281200	-4.50252300
H	-3.67202200	3.53761300	-4.04452800
C	-6.13335600	6.26709900	-2.81886400
H	-6.35924700	5.05165700	-1.05488500
C	-5.51470500	6.40114700	-4.06392100
H	-4.14175600	5.50776900	-5.46898200
H	-6.82852900	7.02677500	-2.46980200
H	-5.72088700	7.26775700	-4.68708600
C	-6.39527100	-2.68971400	-2.84011200
C	-7.65836500	-3.21225600	-2.52218900
C	-5.83279100	-3.00163900	-4.08849800
C	-8.34495000	-4.02091700	-3.43111300
H	-8.10285000	-2.97903100	-1.55831800
C	-6.51919800	-3.80948100	-4.99719100
H	-4.84653100	-2.61631300	-4.33235700
C	-7.77834700	-4.32003200	-4.67214300
H	-9.32409900	-4.41417800	-3.16867800
H	-6.06628400	-4.04384200	-5.95745500
H	-8.31270500	-4.94923800	-5.37979800
C	-2.72599100	-3.82856800	2.99539700
C	-1.51299200	-4.55612400	2.89854000
C	-3.70207500	-4.25369900	3.90900300
C	-1.33439000	-5.68558200	3.72340200
C	-3.51717800	-5.36864400	4.72331400
H	-4.62479600	-3.68305200	3.96930600
C	-2.32342000	-6.08144500	4.61955000
H	-0.40894000	-6.23672400	3.66038100
H	-4.29110400	-5.67125000	5.42363000
H	-2.15043300	-6.95843200	5.23892800
H	0.71526000	-0.86622100	1.80689800
N	-0.53596400	-4.16817100	1.95147100
N	0.13790000	3.50585000	2.36610500
C	0.76473500	-4.62698600	1.89400400
O	1.28056500	-5.32316000	2.77111600
C	1.33656900	4.05283300	1.96060000
O	2.07809500	4.70539000	2.69515900
C	1.69966900	3.80552300	0.52216400
C	3.05915500	3.84594500	0.17908000
C	0.74022300	3.60699600	-0.48128600
C	3.47714600	3.67017200	-1.14318100
H	3.78378300	4.02963200	0.96675500
C	1.14715500	3.42717100	-1.80177600
H	-0.31989200	3.62871000	-0.25178200
C	2.50267000	3.45231000	-2.12721600
H	0.40309600	3.27326300	-2.57828800
H	2.80927700	3.31577100	-3.16168300
C	1.56643200	-4.26393000	0.67457800
C	2.96146600	-4.27160400	0.81982100
C	1.01150800	-4.01783700	-0.58767200
C	3.81426100	-4.02871400	-0.25787300
H	3.37203800	-4.48642900	1.80187500
C	1.85611700	-3.78238500	-1.67455900
H	-0.06215900	-4.00201800	-0.73307300

C	3.24268100	-3.78505800	-1.51507900
H	1.42334100	-3.59080000	-2.65269400
H	3.88385700	-3.59360500	-2.37182700
H	-0.32073000	2.88246600	1.71047800
H	-0.78423900	-3.41004900	1.31337700
C	2.12860600	-0.15868800	0.31626900
H	2.34168200	0.75123100	-0.25289400
H	3.02610400	-0.42314900	0.88491500
H	1.93031400	-0.97219800	-0.38689600
C	5.32260500	-4.12242300	-0.05131700
H	5.65176200	-5.12753700	-0.34886300
H	5.52062400	-4.05071200	1.02379200
C	6.18348100	-3.12413800	-0.80183600
C	6.21297900	-1.74337700	-0.42520900
C	6.96753900	-3.53226500	-1.85883600
C	6.98590200	-0.81194900	-1.10430800
C	7.80002200	-2.63170400	-2.57225700
H	6.96133600	-4.57906400	-2.15626400
C	7.81508000	-1.25008100	-2.19339300
C	8.66102500	-0.36491400	-2.92058000
C	9.44358600	-0.82010700	-3.96027000
H	8.68606200	0.68433700	-2.64611100
H	10.08287000	-0.12461800	-4.49806700
C	9.42203100	-2.18504100	-4.33356700
C	8.61516800	-3.06829000	-3.65167400
H	10.04166300	-2.53143400	-5.15648700
H	8.58887900	-4.11950300	-3.92985300
C	6.96318300	0.63692700	-0.71260300
C	7.97909900	1.17374400	0.14860600
C	5.97894400	1.48258400	-1.20901800
C	7.94524400	2.56474100	0.48937300
C	5.95531200	2.87994000	-0.89907400
C	6.92270600	3.38245700	-0.05641700
H	6.92133500	4.44263400	0.18873800
C	4.94054800	3.81238400	-1.54026400
H	5.25854600	4.83835300	-1.31441200
H	5.00429900	3.71468400	-2.63019200
C	9.03080000	0.37727700	0.68486200
C	9.98717000	0.92439000	1.51348800
H	9.07217800	-0.67766600	0.43448900
H	10.78022900	0.29577000	1.91032600
C	8.94394900	3.09734400	1.34889500
C	9.94569000	2.29791100	1.85233000
H	8.90080300	4.15427900	1.60182600
H	10.70384200	2.71683000	2.50869800
O	4.95042300	0.98572700	-1.97789500
O	5.40119500	-1.34825400	0.61421000
C	5.17610200	0.89257100	-3.38533700
H	5.50378500	1.84974700	-3.81271200
H	5.92046400	0.12655300	-3.62593300
H	4.21743700	0.61100900	-3.82771300
C	5.99613800	-1.35466000	1.91456100
H	6.46470600	-2.32076800	2.14370400
H	6.74554800	-0.56260900	2.01817700
H	5.18333200	-1.17579300	2.62250200
N	-2.35114700	-0.75153900	-2.05761500
N	-2.20711300	-1.90952700	-2.28272000

^{1os}**TS2-2**$^{-}$ ub3lyp/def2tzvp, e. e. = -5585.066729 a.u.
im. frequency -457.81

N	-3.66709800	2.16814300	0.49015900
Fe	-3.94287700	0.15898200	0.32490400
C	-2.12223100	0.10565300	-0.62674800
C	-4.69306900	2.92318700	-1.62986300
C	-5.50914400	-1.88426900	-1.93923300
C	-5.48733700	-0.50537400	-2.20297400
C	-6.13602700	0.10441800	-3.34254300
C	-5.89128600	1.43923800	-3.27016200
C	-5.10123100	1.65630700	-2.07842800
N	-4.87454300	0.45964600	-1.44270100
H	-6.70950800	-0.42410000	-4.09027000
H	-6.23049600	2.21206800	-3.94473300
C	-3.61101300	4.43841900	0.06794400
C	-3.04116400	2.81451200	1.52590100
C	-3.01581800	4.23948900	1.27449100
H	-3.76146800	5.37540500	-0.44822200
H	-2.59591900	4.98332500	1.93696300
C	-4.85275700	-3.91517800	-0.60958700
C	-3.75629800	-2.80665700	1.01496100
C	-4.15623400	-4.11475800	0.53776400
H	-5.30256600	-4.65869300	-1.25172400
H	-3.93113700	-5.05251400	1.02553900
C	-2.47378600	2.21903100	2.66614500
C	-3.06410400	-2.59678400	2.21529400
C	-2.04092500	-1.11132000	3.96483800
C	-2.46665200	0.83783000	2.91341500
C	-1.87128600	0.23026900	4.08841400
H	-1.72740900	-1.88866300	4.64724200
H	-1.38971700	0.76734200	4.89287900
C	-2.73241800	-1.33054700	2.71172400
C	-4.02755200	3.13873900	-0.41270600
C	-4.88189900	-2.48383400	-0.83817900
N	-4.19343300	-1.83684700	0.15402700
N	-2.98081000	-0.13356900	2.09487300
C	-0.96705000	-0.43488400	0.09993000
O	-0.73422800	-1.61698000	0.32543500
O	-0.13221000	0.55741100	0.54207400
C	1.06769300	0.13507400	1.23459600
H	1.30563500	0.96073400	1.91014200
O	-5.54639200	0.20873300	1.29332000
C	-6.53512700	1.16261500	1.11509100
H	-6.91767600	1.21745700	0.07778800
H	-7.40253900	0.92002800	1.75845900
H	-6.21896200	2.18753600	1.39169700
H	-1.90919600	1.06908200	-1.08648100
C	-1.86887600	3.11768100	3.70900500
C	-2.58043900	3.34567700	4.89447200
C	-0.60141700	3.73748600	3.54979100
C	-2.07765100	4.15692000	5.91143800
H	-3.55106600	2.87033700	5.00593400
C	-0.09325300	4.55101700	4.57819600
C	-0.82984400	4.75578300	5.74347300
H	-2.65419500	4.31651200	6.81863900
H	0.87685500	5.00841300	4.44967100
H	-0.41531500	5.38980900	6.52351700
C	-5.00950700	4.11036500	-2.48782700
C	-4.39495000	4.27768000	-3.74049000
C	-5.93014300	5.08417100	-2.06423700
C	-4.68841200	5.38328800	-4.54125700
H	-3.67861700	3.53549800	-4.08227200

C	-6.22340200	6.19042200	-2.86371300
H	-6.42286700	4.96495700	-1.10325500
C	-5.60276500	6.34482300	-4.10546700
H	-4.19698500	5.49472600	-5.50479400
H	-6.94207600	6.92889500	-2.51673100
H	-5.83044800	7.20644900	-4.72807500
C	-6.22813200	-2.77438100	-2.90829000
C	-7.43728700	-3.39430600	-2.55333300
C	-5.70696600	-3.01221600	-4.19086500
C	-8.10730100	-4.22465400	-3.45425600
H	-7.85245400	-3.21583500	-1.56520400
C	-6.37642400	-3.84221200	-5.09227000
H	-4.76556800	-2.54947900	-4.47326400
C	-7.57948200	-4.45097600	-4.72748500
H	-9.04425000	-4.69181800	-3.16084700
H	-5.95299600	-4.01736100	-6.07832300
H	-8.10051200	-5.09784100	-5.42906200
C	-2.75290500	-3.79372900	3.06859300
C	-1.53347500	-4.51316900	2.99006300
C	-3.73153100	-4.21405000	3.98094100
C	-1.34943600	-5.62856500	3.83210000
C	-3.54235800	-5.31623300	4.81207100
H	-4.65969600	-3.65095600	4.02667700
C	-2.34175700	-6.01980800	4.72714900
H	-0.41835900	-6.17170600	3.78265800
H	-4.31866600	-5.61606000	5.51096800
H	-2.16559700	-6.88584800	5.36089300
H	0.84299700	-0.75194000	1.83013400
N	-0.55663300	-4.12734000	2.04192900
N	0.09724300	3.52960900	2.33976300
C	0.74018600	-4.59344400	1.97247500
O	1.25937200	-5.29999000	2.83931300
C	1.29782400	4.07353100	1.93458700
O	2.03669100	4.73251400	2.66636800
C	1.66868000	3.82056600	0.49860100
C	3.03074500	3.85187900	0.16358400
C	0.71341100	3.63370600	-0.51080400
C	3.45558400	3.67827100	-1.15694300
H	3.75195400	4.02784300	0.95617200
C	1.12666900	3.45939400	-1.83025300
H	-0.34742000	3.65996200	-0.28495100
C	2.48437800	3.47500100	-2.14772000
H	0.38597200	3.31738600	-2.61231000
H	2.79569400	3.34323700	-3.18150100
C	1.53310500	-4.22781200	0.74741100
C	2.92964900	-4.24695200	0.87621500
C	0.96560500	-3.97218800	-0.50728300
C	3.77213200	-4.00676900	-0.21068200
H	3.34980500	-4.46942600	1.85252400
C	1.79915200	-3.73977200	-1.60330100
H	-0.11003100	-3.95264900	-0.63916400
C	3.18762200	-3.75413700	-1.46007000
H	1.35687600	-3.54289100	-2.57617000
H	3.82005500	-3.56559100	-2.32391700
H	-0.35182700	2.88857300	1.69365900
H	-0.80202900	-3.35894000	1.41554000
C	2.19170700	-0.12231600	0.24835700
H	2.39513900	0.76613800	-0.35670100
H	3.11309300	-0.39558400	0.77204300
H	1.93346800	-0.94841500	-0.42045000
C	5.28238600	-4.11457200	-0.02295100
H	5.59800400	-5.12229100	-0.32625900
H	5.49409300	-4.04772900	1.04984500
C	6.14523000	-3.12357000	-0.78174500
C	6.18884600	-1.74259400	-0.40747300
C	6.91763500	-3.53852700	-1.84467100
C	6.95824900	-0.81596600	-1.09734200
C	7.75079500	-2.64465100	-2.56587500
H	6.90152100	-4.58555100	-2.14090700
C	7.77662800	-1.26195400	-2.19114200
C	8.62320800	-0.38388300	-2.92610800
C	9.39648400	-0.84666500	-3.96937100
H	8.65641600	0.66585500	-2.65422400
H	10.03696200	-0.15669700	-4.51280400
C	9.36395100	-2.21242500	-4.33894600
C	8.55607100	-3.08891400	-3.64955900
H	9.97628400	-2.56485400	-5.16475700
H	8.52167200	-4.14069000	-3.92472200
C	6.94051400	0.63492200	-0.71131300
C	7.95411000	1.17178700	0.15234700
C	5.96027800	1.48196700	-1.21373000
C	7.91886000	2.56280300	0.49315000
C	5.93367000	2.87896000	-0.90220300
C	6.89752400	3.38059400	-0.05487700
H	6.89471900	4.44040200	0.19183700
C	4.92204100	3.81261000	-1.54750000
H	5.24288000	4.83799300	-1.32292500
H	4.98911200	3.71262500	-2.63700300
C	9.00573900	0.37599400	0.68976800
C	9.96041900	0.92359600	1.52005000
H	9.04902500	-0.67866700	0.43833900
H	10.75375600	0.29564000	1.91737300
C	8.91549200	3.09569200	1.35492600
C	9.91697100	2.29684000	1.85976800
H	8.87117300	4.15250100	1.60814100
H	10.67361500	2.71607600	2.51765600
O	4.93699200	0.98588900	-1.99029700
O	5.39101100	-1.34029600	0.63985800
C	5.16957400	0.89957300	-3.39700100
H	5.50877500	1.85608300	-3.81667900
H	5.90753800	0.12764500	-3.63837900
H	4.21053100	0.63049800	-3.84648700
C	5.99310500	-1.36144700	1.93688400
H	6.44622700	-2.33610900	2.16037600
H	6.75656500	-0.58272400	2.03850600
H	5.18695800	-1.17098200	2.64940100
N	-2.18740600	-0.86526500	-2.16275500
N	-1.98584900	-1.98782600	-2.23338000

[2]**FP** ub3lyp/def2tzvp, e. e. = -2367.868447 a.u.

C	1.69729900	2.48477000	-0.22847800
N	1.61862300	1.10979700	-0.24627600
H	3.39589200	3.94414300	-0.32787300
C	2.92015300	0.66640900	-0.34823500
C	3.83011000	1.78278000	-0.39563700
H	4.90596400	1.69657100	-0.48307400
C	3.07180400	2.91097400	-0.31670200
C	3.30932900	-0.66447100	-0.39032800
N	1.06692900	-1.65578600	-0.24606100
C	0.61371000	-2.95635300	-0.20665200

C	1.72072700	-3.87584300	-0.28400900
H	1.62493700	-4.95442500	-0.27967100
C	2.85328700	-3.12463400	-0.37240300
H	3.88032300	-3.45791200	-0.45349800
C	2.43972600	-1.74518500	-0.34245900
C	0.61421400	3.35088100	-0.17108300
H	0.82543300	4.41569800	-0.15483900
H	-3.99311100	3.45640100	-0.21216900
C	-2.96177400	3.12676500	-0.20058000
C	-2.54164500	1.74732500	-0.21134200
H	-1.73605700	4.95976500	-0.16028300
N	-1.16785300	1.66187100	-0.20694400
C	-0.71742000	2.96197500	-0.17459500
C	-1.82932800	3.88101900	-0.17393600
C	-0.71674500	-3.34583900	-0.14109300
H	-0.92443800	-4.41120100	-0.11592900
H	-5.01785800	-1.68921900	-0.19628100
C	-3.93885300	-1.77863100	-0.18043400
C	-3.18025800	-2.90765400	-0.14641700
H	-3.50577900	-3.94028100	-0.12972000
C	-1.80153800	-2.48115000	-0.14646800
N	-1.72184800	-1.10887000	-0.18199700
C	-3.02289000	-0.66368900	-0.19538500
C	-3.41246200	0.66741900	-0.21106400
H	-4.47703000	0.88030800	-0.21419900
H	4.37143400	-0.87637100	-0.46587100
Fe	-0.04041700	0.00006000	0.00841000
O	-0.11504000	0.01167100	1.78684000
C	1.04890700	-0.03769700	2.57148400
H	0.73735500	-0.26727700	3.60204300
H	1.75256100	-0.81994700	2.25410100
H	1.58246800	0.92471000	2.58772800

[4]FP ub3lyp/def2tzvp, e. e. = -2367.875729 a.u.

C	-1.15493400	-2.78919400	-0.29752400
N	-1.35320500	-1.42632300	-0.28688700
H	-2.53212700	-4.55322700	-0.39948900
C	-2.71670900	-1.24553700	-0.35658000
C	-3.38709700	-2.51962500	-0.41045300
H	-4.46041200	-2.64796700	-0.47377700
C	-2.41841700	-3.47676900	-0.37244400
C	-3.36619400	-0.01913500	-0.37705000
N	-1.36932700	1.41057600	-0.28563100
C	-1.18639100	2.77572400	-0.29741700
C	-2.45760700	3.44891100	-0.37307500
H	-2.58366900	4.52396800	-0.40088200
C	-3.41526200	2.48071700	-0.41034000
H	-4.48995000	2.59679100	-0.47411500
C	-2.73065100	1.21433200	-0.35577900
C	0.07800100	-3.42368500	-0.25561800
H	0.08538300	-4.50893700	-0.27081800
H	4.61068100	-2.59447100	-0.17988500
C	3.53405800	-2.47950700	-0.18597300
C	2.84582500	-1.21332000	-0.17193000
H	2.70372100	-4.52252600	-0.23803000
N	1.48472200	-1.40945500	-0.19264600
C	1.30307300	-2.77307600	-0.21827000
C	2.57669900	-3.44748400	-0.21539800
C	0.03930400	3.42427900	-0.25618600
H	0.03457500	4.50953700	-0.27223100
H	4.58109400	2.64592800	-0.18269400
C	3.50582700	2.51884300	-0.18786700
C	2.53765300	3.47598700	-0.21708900
H	2.65253800	4.55237400	-0.24053400
C	1.27157300	2.78757300	-0.21875600
N	1.46830100	1.42606800	-0.19222100
C	2.83170600	1.24507000	-0.17231800
C	3.48180800	0.01955400	-0.15336800
H	4.56702400	0.02570400	-0.13767200
H	-4.45021000	-0.02509900	-0.43122900
Fe	0.04671000	0.00023300	0.07161000
O	-0.00178400	-0.00444400	1.92729400
C	-1.07297900	-0.00041500	2.81545400
H	-0.70098600	-0.06133600	3.85196100
H	-1.67633600	0.92089000	2.73874100
H	-1.75066600	-0.85765000	2.66002300

[6]FP ub3lyp/def2tzvp, e. e. = -2367.880187 a.u.

N	-1.16353200	1.65371200	-0.27958900
Fe	0.03993000	0.00479400	0.23555200
C	-3.34409600	0.52788700	-0.41260200
C	-0.46676400	-3.38792700	-0.31946900
C	-1.60178500	-2.57663900	-0.34729300
C	-2.95929300	-3.05387100	-0.47013100
C	-3.76740500	-1.95457100	-0.49892200
C	-2.90930100	-0.79782500	-0.39296700
N	-1.59868800	-1.20225800	-0.29378400
H	-3.24445000	-4.09643500	-0.53864400
H	-4.84549100	-1.91854400	-0.59534400
C	-3.00559100	3.02661600	-0.45834500
C	-0.75355600	2.96527500	-0.30668200
C	-1.90492500	3.83151400	-0.41421100
H	-4.04485300	3.31750600	-0.54922000
H	-1.86360400	4.91263600	-0.46228100
C	2.01666000	-3.82196400	-0.30768900
C	2.63720800	-1.65266600	-0.23172400
C	3.11621800	-3.01555600	-0.27914600
H	1.97980200	-4.90335200	-0.35346300
H	4.15978400	-3.30457700	-0.29670400
C	0.57267900	3.39667000	-0.26772800
C	3.44832000	-0.51740300	-0.21100500
C	3.87891900	1.96633600	-0.23745700
C	1.70815100	2.58567300	-0.23401800
C	3.07092500	3.06537900	-0.25147600
H	4.96121100	1.93029400	-0.25131100
H	3.35966900	4.10877300	-0.27971000
C	3.01433200	0.80885300	-0.21130500
C	-2.53384700	1.66338000	-0.37591500
C	0.85954200	-2.95612400	-0.27784900
N	1.26433300	-1.64517700	-0.22935500
N	1.70183800	1.21277100	-0.20700000
O	0.00303200	0.00146700	2.05416500
C	-0.97947400	-0.09708000	3.04286100
H	-1.63515900	-0.96656800	2.88124500
H	-0.50401200	-0.21011800	4.02895800
H	-1.60936300	0.80619300	3.07466100
H	-4.41465700	0.69141200	-0.49485500
H	-0.63136700	-4.46051900	-0.36518200
H	4.52185400	-0.68180400	-0.21527500
H	0.73904800	4.46963600	-0.29399500

^{2}FP-EDA ub3lyp/def2tzvp, e. e. = -2784.021704 a.u.

C	0.52270400	3.10267200	-0.04437000
N	0.72690700	1.85463700	0.50171500
H	1.78078700	4.95091700	-0.20539600
C	2.00426100	1.88115800	1.01787300
C	2.61081500	3.16929100	0.79566500
H	3.61288900	3.43956500	1.10456600
C	1.69010000	3.92842800	0.13942700
C	2.62552200	0.82277800	1.66532300
N	0.77998400	-0.78727500	1.48469800
C	0.61914500	-2.08631000	1.91512900
C	1.81181900	-2.54533300	2.58171600
H	1.93867400	-3.53533200	3.00147100
C	2.70079200	-1.51416500	2.55558100
H	3.70752700	-1.47979100	2.95283700
C	2.05008300	-0.42215000	1.87774200
C	-0.62364200	3.51006900	-0.71297800
H	-0.64856300	4.52867700	-1.08798200
H	-4.72772000	2.02048900	-2.19180500
C	-3.74888500	2.08265100	-1.73293200
C	-3.10482300	1.00289100	-1.02711300
H	-3.02619100	4.13172500	-2.11278200
N	-1.86498900	1.39488600	-0.57460900
C	-1.72841600	2.70881400	-0.96391500
C	-2.89486800	3.14212500	-1.69307200
C	-0.51352000	-2.86595800	1.72662900
H	-0.49810100	-3.87392000	2.12931700
H	-4.66508100	-2.86391100	-0.27737500
C	-3.68782800	-2.57564700	0.08895300
C	-2.80061900	-3.31070900	0.81196900
H	-2.89580000	-4.33032600	1.16367900
C	-1.64876100	-2.46963200	1.03485300
N	-1.83029500	-1.23953000	0.44886900
C	-3.07830600	-1.28549700	-0.12711800
C	-3.67669500	-0.24477200	-0.82179700
H	-4.66906900	-0.41557700	-1.22754700
C	0.54684400	-1.00260600	-2.50387700
C	1.86449600	-1.56988800	-2.23454100
O	2.10517600	-2.75711900	-2.09729400
O	2.78604800	-0.58477900	-2.17336300
C	4.14788400	-1.00988300	-1.93199500
H	4.40552600	-1.79709800	-2.64710300
H	4.21027000	-1.43675400	-0.92555000
N	-1.29982100	-2.58515200	-2.73254300
N	-0.45023400	-1.83916700	-2.62565500
H	3.63549600	0.97937600	2.03161800
Fe	-0.62750400	0.37533100	0.65825200
C	5.03802500	0.20760500	-2.08563600
H	4.75603900	0.99306900	-1.37663300
H	6.08062100	-0.07163800	-1.89502300
H	4.97235100	0.61703700	-3.09949000
H	0.32914800	0.05026600	-2.61941800
O	-1.45239800	0.91697600	2.13908000
C	-0.75437600	1.52261800	3.19762700
H	-1.42951000	1.53067500	4.06700800
H	0.15529700	0.97796700	3.48699900
H	-0.48146800	2.56529000	2.97448400

^{4}FP-EDA ub3lyp/def2tzvp, e. e. = -2784.029255 a.u.

C	0.51411400	3.08712300	-0.07910200
N	0.69854000	1.84934400	0.49700300
H	1.77850600	4.93087800	-0.22096200
C	1.95418500	1.88051900	1.06123600
C	2.56663200	3.16603400	0.84283300
H	3.55507900	3.44284700	1.18771400
C	1.67369800	3.91393200	0.13576800
C	2.56528600	0.82741800	1.72792100
N	0.75396700	-0.81302000	1.47622500
C	0.61441400	-2.12979100	1.85391400
C	1.80449100	-2.58464800	2.52660800
H	1.94773600	-3.58614400	2.91225700
C	2.66685100	-1.53074600	2.55869700
H	3.66359600	-1.48695100	2.97953200
C	2.00520500	-0.43038900	1.90412400
C	-0.60932400	3.48675800	-0.78935000
H	-0.61832800	4.49637600	-1.18771700
H	-4.70782700	1.99588400	-2.28469700
C	-3.73397700	2.05963100	-1.81566000
C	-3.11014700	0.99167100	-1.07634300
H	-2.98459300	4.09026900	-2.24292300
N	-1.87491200	1.38740400	-0.61757400
C	-1.71619300	2.68771100	-1.04018100
C	-2.86855400	3.11153600	-1.79447400
C	-0.50291600	-2.92010500	1.62605400
H	-0.47658500	-3.94280200	1.98820500
H	-4.65343900	-2.88667700	-0.37805900
C	-3.67986000	-2.59953800	-0.00135500
C	-2.78319600	-3.35080600	0.69517100
H	-2.86697800	-4.38351100	1.00926600
C	-1.64171000	-2.50825300	0.94862600
N	-1.84210600	-1.25891700	0.40881000
C	-3.08601500	-1.29789300	-0.17663800
C	-3.68654800	-0.25317300	-0.86484300
H	-4.67408200	-0.42723800	-1.28019900
C	0.63312100	-0.96724600	-2.48284900
C	1.95519500	-1.52415900	-2.21667000
O	2.21309800	-2.71091000	-2.10854800
O	2.86140200	-0.52711800	-2.12067200
C	4.22615900	-0.93458800	-1.86843400
H	4.50947600	-1.69944800	-2.59801800
H	4.28106900	-1.38432700	-0.87140700
N	-1.19947900	-2.55684000	-2.76579100
N	-0.35520100	-1.80852000	-2.63143100
H	3.56245300	0.99399600	2.12306400
Fe	-0.68792300	0.38414000	0.69971700
C	5.09615900	0.30202900	-1.97860000
H	4.78879600	1.06446300	-1.25518400
H	6.14077700	0.03728600	-1.77867300
H	5.03785500	0.73506800	-2.98301100
H	0.40248000	0.08691300	-2.54938000
O	-1.48271900	0.95874000	2.27887500
C	-0.93203400	1.46986100	3.45028900
H	-1.70643700	1.53176200	4.23313900
H	-0.11822200	0.83743500	3.84459600
H	-0.52545900	2.48711300	3.31144800

^{6}FP-EDA ub3lyp/def2tzvp, e. e. = -2784.033194 a.u.

C	0.50747800	3.08118200	-0.12557300
N	0.69462700	1.85803300	0.47495800

H	1.78458900	4.92377300	-0.27226200
C	1.93679100	1.88868700	1.06208700
C	2.55071700	3.17561800	0.83129600
H	3.53362100	3.46494300	1.18241300
C	1.66711600	3.91259200	0.09731100
C	2.52633500	0.82959800	1.75366700
N	0.73283700	-0.83551800	1.52056700
C	0.59079800	-2.15388400	1.88160300
C	1.78318000	-2.60178000	2.56383400
H	1.93837700	-3.60337100	2.94517600
C	2.64027800	-1.54147100	2.60503800
H	3.63547100	-1.50145200	3.03040700
C	1.97712200	-0.43873200	1.94703700
C	-0.61698700	3.45202600	-0.86427400
H	-0.61898200	4.45466200	-1.28191900
H	-4.66769100	1.91862000	-2.49421100
C	-3.71583400	1.99288600	-1.98299200
C	-3.11350500	0.93760700	-1.20156800
H	-2.97173600	4.02185100	-2.41881400
N	-1.90507400	1.36242700	-0.70463900
C	-1.72869000	2.65429300	-1.13975500
C	-2.86007300	3.05492300	-1.94397600
C	-0.52464800	-2.94793700	1.61486500
H	-0.49002300	-3.97425600	1.96812200
H	-4.61330300	-3.00742200	-0.54922600
C	-3.66482500	-2.69398400	-0.13112900
C	-2.77902600	-3.43189000	0.59699300
H	-2.85707700	-4.47045700	0.89352800
C	-1.66305600	-2.56644500	0.90402200
N	-1.88035600	-1.32219500	0.36564100
C	-3.09374200	-1.37410400	-0.27563400
C	-3.67037900	-0.32487800	-0.99211000
H	-4.63610400	-0.51696800	-1.45050900
C	0.76265700	-0.94122600	-2.41827700
C	2.09000100	-1.49473000	-2.17650600
O	2.36234200	-2.68236300	-2.12393600
O	2.98501500	-0.49249000	-2.03481000
C	4.35206600	-0.89168000	-1.78669600
H	4.64771800	-1.63718500	-2.53114700
H	4.40724000	-1.36208200	-0.79888900
N	-1.04825400	-2.53559600	-2.79717600
N	-0.21442300	-1.78537200	-2.61448600
H	3.52039200	1.00031100	2.15684000
Fe	-0.80712100	0.43039300	0.83692600
C	5.20965000	0.35622000	-1.86423500
H	4.88895700	1.09988600	-1.12717600
H	6.25568600	0.09856400	-1.66245300
H	5.15327700	0.81007800	-2.85950800
H	0.51430300	0.11092100	-2.40892300
O	-1.59543500	1.02577700	2.36457400
C	-1.15909000	1.65038500	3.53660400
H	-1.96542600	1.63868400	4.28529300
H	-0.28560000	1.14011600	3.97112900
H	-0.88543300	2.70105700	3.35158500

[2]**TS2** ub3lyp/def2tzvp, e. e. = -2783.983504 a.u.
im. frequency -297.87

C	-0.52578500	3.02173300	-0.25938400
N	-0.83874900	1.71708800	-0.55862700
H	-1.68880500	4.93923200	-0.34775100
C	-2.14530500	1.71711900	-0.98417000
C	-2.67100400	3.06085700	-0.95716600
H	-3.67628400	3.33586100	-1.25154100
C	-1.67169200	3.86718000	-0.50059600
C	-2.85815400	0.58875200	-1.37264100
N	-1.09087300	-1.07943700	-0.99576200
C	-1.02434200	-2.44373200	-1.13702900
C	-2.29532700	-2.95375600	-1.59437400
H	-2.50741000	-3.99863400	-1.78430000
C	-3.12308600	-1.88102600	-1.74147600
H	-4.15280600	-1.86504800	-2.07690000
C	-2.36274000	-0.71236400	-1.36508600
C	0.71439400	3.47817000	0.17669300
H	0.80852700	4.54294000	0.36844100
H	5.08836200	2.17776200	0.88641900
C	4.02445700	2.17700800	0.68359000
C	3.25174500	1.00373000	0.34373200
H	3.36839900	4.27247600	0.89012000
N	1.93882100	1.34637200	0.15003900
C	1.85948500	2.70128000	0.33817900
C	3.15977100	3.22912800	0.68796900
C	0.10170000	-3.22840800	-0.91296700
H	-0.00105800	-4.29770800	-1.07122200
H	4.61805500	-3.00827100	0.01436100
C	3.57577400	-2.75851000	-0.14085300
C	2.53903500	-3.58852900	-0.43790900
H	2.55296800	-4.66176300	-0.58194600
C	1.36043300	-2.75745400	-0.55253500
N	1.68016100	-1.44986300	-0.30405100
C	3.02903100	-1.42157300	-0.06320000
C	3.76946400	-0.28315300	0.24013600
H	4.83342500	-0.41088900	0.41521000
C	-0.22891300	0.11874500	1.41679200
C	-1.33128100	-0.73586800	1.98581700
O	-1.20287800	-1.86152900	2.42005800
O	-2.48478200	-0.05675000	1.92642500
C	-3.68796800	-0.79171800	2.29043400
H	-3.54070200	-1.22237600	3.28494800
H	-3.82009400	-1.61111800	1.57764000
N	2.08610800	-1.10408200	2.98189300
N	1.18317400	-0.58648700	2.61108200
H	-3.88482400	0.73088000	-1.69655800
Fe	0.43069300	0.13178100	-0.45946400
C	-4.84335200	0.18637600	2.25815700
H	-4.96195200	0.62135200	1.26064900
H	-5.77042000	-0.33584800	2.52061400
H	-4.69214200	0.99941800	2.97626000
H	-0.16894100	1.09151000	1.90799500
O	1.01308100	0.31998400	-2.17444500
C	0.22432700	0.69730300	-3.26112400
H	0.84198300	0.65621000	-4.17296200
H	-0.63673500	0.02647300	-3.41540700
H	-0.16542500	1.72473700	-3.17227800

[4]**TS2** ub3lyp/def2tzvp, e. e. = -2783.956276 a.u.
im. frequency -327.72

C	-0.79930200	2.93238500	-0.42662800
N	-0.99706300	1.58909600	-0.63677300
H	-2.15635500	4.70919700	-0.67798600
C	-2.28274900	1.41090600	-1.08299000

C	-2.93230400	2.70232100	-1.15541300
H	-3.95220400	2.86600200	-1.48194500
C	-2.02297200	3.63637300	-0.74664200
C	-2.86497400	0.18056500	-1.41401200
N	-0.98601400	-1.33085700	-0.94024400
C	-0.75243800	-2.67424100	-1.04437600
C	-1.95733500	-3.32430900	-1.52052300
H	-2.06207000	-4.38843700	-1.69397400
C	-2.89203400	-2.34656900	-1.71219000
H	-3.90697900	-2.45738200	-2.07446000
C	-2.26829000	-1.08854100	-1.34982900
C	0.40094900	3.52723600	-0.01449500
H	0.38607100	4.60895600	0.08754200
H	4.88919300	2.70866600	0.86106100
C	3.83388300	2.58808200	0.64806600
C	3.17394600	1.32072100	0.39593200
H	3.00660100	4.63093000	0.66410900
N	1.84586000	1.54990400	0.17033300
C	1.63048700	2.89621300	0.23326200
C	2.88069600	3.56098300	0.55029000
C	0.46860300	-3.30673800	-0.77477700
H	0.49074700	-4.38400100	-0.91531300
H	4.94030900	-2.59873700	0.24574000
C	3.88164400	-2.45629100	0.06595300
C	2.94651200	-3.40117000	-0.23879700
H	3.09049700	-4.46798400	-0.35944000
C	1.67977500	-2.70872000	-0.39600700
N	1.86853300	-1.37769900	-0.15977900
C	3.19505600	-1.17751100	0.10290000
C	3.79243600	0.06271300	0.37166000
H	4.86091500	0.04788100	0.56898600
C	-0.28272700	0.18147900	1.39846600
C	-1.41267600	-0.64956600	1.95409800
O	-1.29801500	-1.77221100	2.39907600
O	-2.55600100	0.04292700	1.88352400
C	-3.76883200	-0.67427900	2.25263400
H	-3.62210500	-1.11079200	3.24452300
H	-3.91551800	-1.48869500	1.53689000
N	1.96667900	-1.03953700	3.02081700
N	1.07901400	-0.49270000	2.65379000
H	-3.89291300	0.21400500	-1.76490700
Fe	0.45740300	0.10554300	-0.46695200
C	-4.91046200	0.32023700	2.23119800
H	-5.03508400	0.75760100	1.23564600
H	-5.84163700	-0.18931000	2.50372700
H	-4.74090000	1.13095300	2.94778900
H	-0.22591500	1.17379300	1.84579400
O	1.06838500	0.19620000	-2.17310700
C	0.31147100	0.66878900	-3.25171900
H	0.89445800	0.49670400	-4.17057800
H	-0.64698500	0.13925200	-3.36429400
H	0.10321600	1.74810300	-3.18746000

[6]**TS2** ub3lyp/def2tzvp, e. e. = -2783.967982 a.u.
im. frequency -384.47

C	-1.53457200	2.28626600	-0.88974200
N	-1.29192500	0.92993900	-0.80383600
H	-3.31686100	3.44817300	-1.60394900
C	-2.39472400	0.26814700	-1.30080200
C	-3.38437400	1.24472600	-1.68129200
H	-4.35016900	1.01274500	-2.11382500
C	-2.85847500	2.48350700	-1.42354900
C	-2.51221200	-1.12137300	-1.43186800
N	-0.26090300	-1.82323700	-0.73932800
C	0.37944200	-3.01519200	-0.55433400
C	-0.53197800	-4.09777100	-0.87230600
H	-0.29054000	-5.15189200	-0.81171100
C	-1.71364000	-3.52676400	-1.24574200
H	-2.62803300	-4.02295400	-1.54708800
C	-1.53614000	-2.09005600	-1.15151800
C	-0.61696800	3.29397600	-0.56789100
H	-0.97950900	4.31463900	-0.65320300
H	3.74177000	4.14558800	0.78239600
C	2.82749500	3.65048200	0.47882500
C	2.66219700	2.21663300	0.34406300
H	1.37585700	5.27484800	0.14337600
N	1.38366100	1.94981500	-0.05794400
C	0.72394900	3.14258600	-0.18146000
C	1.63053900	4.22213000	0.15479000
C	1.70679600	-3.16157600	-0.13068700
H	2.07417100	-4.18010000	-0.04091600
H	5.54587300	-0.87381900	1.11151500
C	4.53689700	-1.11440000	0.79973700
C	3.98979400	-2.34980000	0.60361400
H	4.46494300	-3.31561200	0.72386200
C	2.61694700	-2.14578900	0.19030200
N	2.35619100	-0.80121500	0.15544300
C	3.50309800	-0.14201800	0.51173100
C	3.64444500	1.24929000	0.59657900
H	4.61941500	1.61730100	0.90380600
C	-0.59174600	0.32106900	1.36721900
C	-1.79423100	-0.41434500	1.90929100
O	-1.73720000	-1.50077400	2.45283600
O	-2.93934800	0.26981200	1.72087200
C	-4.15927600	-0.40679800	2.11891500
H	-4.08937700	-0.65321600	3.18302500
H	-4.23766800	-1.34547700	1.56131700
N	1.58569200	-0.49091600	3.21972200
N	0.61385000	-0.05912400	2.90363100
H	-3.46526100	-1.48927600	-1.80218300
Fe	0.64457300	0.07128400	-0.63276300
C	-5.31856600	0.52583700	1.83156400
H	-5.37465700	0.77022100	0.76599200
H	-6.25802500	0.04479700	2.12682100
H	-5.22117300	1.46039600	2.39433000
H	-0.63348800	1.37983100	1.63392700
O	1.10075800	0.16400400	-2.41548400
C	0.55220800	-0.14894200	-3.65430200
H	1.26351500	0.09599800	-4.45969600
H	0.31372100	-1.22255300	-3.73494200
H	-0.37449700	0.41683500	-3.84744100

[2]**TC** ub3lyp/def2tzvp, e. e. = -2674.429051 a.u.

C	2.47629100	2.15447200	0.59532200
N	1.21785100	1.86476500	0.13069400
H	3.50547900	4.06685000	1.15384700
C	0.54838000	3.06033700	0.04323100
C	1.41246100	4.13809500	0.45999400
H	1.12783400	5.18270900	0.47893600
C	2.60742800	3.57752000	0.79792100

C	-0.75807200	3.22184500	-0.40402600
N	-1.25339300	0.86433700	-0.89925400
C	-2.34495600	0.21169400	-1.41279700
C	-3.39001000	1.16157800	-1.71146400
H	-4.35091600	0.90301100	-2.13893400
C	-2.92074200	2.39070400	-1.35768700
H	-3.41678900	3.35028300	-1.43357200
C	-1.58412200	2.19778800	-0.85004000
C	3.47944100	1.22631400	0.85177100
H	4.43032700	1.60715100	1.21141600
H	4.37522300	-3.30128700	0.86759300
C	3.89290800	-2.33780600	0.76000500
C	2.53442000	-2.14626100	0.30666900
H	5.39459500	-0.83405000	1.35105800
N	2.23461700	-0.81082900	0.27831200
C	3.35753700	-0.14980000	0.70282800
C	4.40455400	-1.10003400	1.00206200
C	-2.45487800	-1.16134000	-1.59849300
H	-3.38121700	-1.53641700	-2.02239500
H	-0.18708100	-5.13036900	-0.97049300
C	-0.46132700	-4.08324500	-0.99695400
C	-1.61514600	-3.51882300	-1.44536300
H	-2.48770200	-4.00501100	-1.86347200
C	-1.47815400	-2.09174800	-1.26717000
N	-0.25539300	-1.80320200	-0.71784500
C	0.38750900	-3.00314000	-0.55096700
C	1.67650500	-3.17433800	-0.06430700
H	2.05141200	-4.19016500	0.01068400
C	-0.09986900	-0.05448100	1.44155800
C	-1.30752500	-0.67996600	2.01671700
O	-1.23927900	-1.77581800	2.54601900
O	-2.41618400	0.06081000	1.92323600
C	-3.62855300	-0.51562500	2.48221000
H	-3.42891700	-0.82398300	3.51272600
H	-3.88202400	-1.40824000	1.90155900
H	-1.15825700	4.23070500	-0.42394900
Fe	0.48449200	0.02820700	-0.31352000
C	-4.71238900	0.53996700	2.40345300
H	-4.87705200	0.85649900	1.36842500
H	-5.65097900	0.13073400	2.79422700
H	-4.44869000	1.42118700	2.99801600
H	0.56748500	0.35314200	2.20514100
O	1.17478200	0.12019700	-2.00868400
C	1.04670000	1.14492400	-2.94297700
H	1.58921100	0.86883300	-3.86026500
H	-0.00552700	1.33089800	-3.21177800
H	1.46611600	2.09532300	-2.57416000

[2]**TS3** ub3lyp/def2tzvp, e. e. = -2674.423794 a.u.
im. frequency -82.31

C	0.08988100	3.03931400	-0.17248000
N	-0.43832400	1.79595100	-0.46062800
H	-0.72397100	5.11950800	-0.33212300
C	-1.71087300	2.01295900	-0.94872400
C	-1.99339300	3.42466800	-0.95107200
H	-2.92264800	3.86472500	-1.29108500
C	-0.88597400	4.05705000	-0.46532700
C	-2.56870600	1.02032000	-1.39977500
N	-1.14643300	-0.93162700	-0.91870000
C	-1.31889200	-2.28685000	-1.05340200
C	-2.62181900	-2.56585600	-1.60628100
H	-3.00323000	-3.55820500	-1.81241600
C	-3.22626200	-1.36282700	-1.81830300
H	-4.20814900	-1.16475200	-2.23008100
C	-2.29999000	-0.34659800	-1.38000500
C	1.38371800	3.27646600	0.27298400
H	1.66580200	4.31062300	0.44576800
H	5.35777800	1.20589800	1.38943300
C	4.33937400	1.39832800	1.07561800
C	3.38635200	0.37781200	0.70468900
H	4.08886300	3.58900600	1.13399100
N	2.19558600	0.95355400	0.35046200
C	2.35928400	2.30603600	0.49729200
C	3.70238800	2.59477100	0.94719100
C	-0.37497700	-3.25628400	-0.73467700
H	-0.65843500	-4.29391200	-0.88219700
H	3.97244400	-3.82639600	0.70292900
C	3.02238300	-3.39773800	0.40929100
C	1.89059300	-4.03505100	0.00749000
H	1.71431800	-5.09809000	-0.09977800
C	0.91164400	-3.00651900	-0.27197100
N	1.44692600	-1.76967500	-0.03556500
C	2.73371000	-1.98018800	0.37951600
C	3.64315400	-0.98775000	0.72654900
H	4.63375700	-1.30468300	1.03820900
C	-0.22051600	0.30570900	1.36514700
C	-1.37490800	-0.29380700	2.05022700
O	-1.09364400	-1.15103300	2.87401800
O	-2.59441400	0.14776300	1.77001000
C	-3.68930600	-0.48071800	2.50100900
H	-3.49861800	-0.36806500	3.57231900
H	-3.69444800	-1.54824500	2.26264600
H	-3.53476300	1.33236000	-1.78469500
Fe	0.54227500	-0.00559600	-0.31506000
C	-4.97230800	0.20244000	2.07727200
H	-5.14928800	0.07773500	1.00438400
H	-5.81500000	-0.24172400	2.61901300
H	-4.94383400	1.27321900	2.30522200
H	0.24697200	1.07732400	1.98248100
O	1.22152000	-0.01311900	-2.01248900
C	0.75972300	0.75165600	-3.08323900
H	1.31965100	0.46966300	-3.98977200
H	-0.31046800	0.58591800	-3.29110000
H	0.90644700	1.83320400	-2.92700900

[2]**BC** ub3lyp/def2tzvp, e. e. = -2674.453351 a.u.

N	-0.93460900	1.65632100	-0.20574200
Fe	0.58354400	-0.10142900	-0.37864000
C	-0.56881700	0.83039200	0.95591700
C	0.83518100	3.37925900	0.03300700
C	3.68940800	-0.44007000	0.94747500
C	3.25187100	0.86485900	0.75279200
C	4.01366300	2.05200200	1.07709800
C	3.20084200	3.11258400	0.82385100
C	1.93929800	2.57304200	0.35311100
N	2.01086600	1.20613600	0.28343700
H	5.02545400	2.05280100	1.46257100
H	3.40835500	4.16713800	0.95724300
C	-1.38875100	3.72220100	-1.08703400
C	-2.08001900	1.55675400	-1.02261700

C	-2.37835900	2.86744000	-1.50100500
H	-1.25997200	4.76114200	-1.36187600
H	-3.19783000	3.08847300	-2.17276500
C	3.41594900	-2.93534800	0.91623200
C	1.31228000	-2.94251300	0.10387100
C	2.41077300	-3.77287200	0.53533700
H	4.39963300	-3.19017900	1.28992500
H	2.40180300	-4.85563500	0.53289200
C	-2.68463600	0.38852200	-1.45311300
C	0.11483700	-3.42871700	-0.41695700
C	-2.18750800	-3.17227400	-1.40306600
C	-2.20037200	-0.91788400	-1.33253300
C	-2.95967100	-2.09404700	-1.70682200
H	-2.41935500	-4.22321500	-1.52222900
H	-3.95912100	-2.07647800	-2.12338600
C	-0.94764300	-2.65372100	-0.86576200
C	-0.43515400	2.97124700	-0.34087300
C	2.93535800	-1.58916500	0.72089800
N	1.64515900	-1.61830600	0.24821000
N	-0.97601400	-1.28420300	-0.84579100
C	-1.57318200	0.14772100	1.81023300
O	-1.22657500	-0.51981000	2.77079500
O	-2.86905000	0.35443200	1.49357800
C	-3.83781900	-0.30823700	2.33902000
H	-3.69412600	0.02750000	3.37106500
H	-3.65389800	-1.38695100	2.31066600
C	-5.21874300	0.04167200	1.82070400
H	-5.97947800	-0.43347600	2.45072400
H	-5.38592000	1.12424000	1.83968600
H	-5.35570200	-0.31218000	0.79332400
O	1.08170800	-0.00920300	-2.13651800
C	2.35262700	0.28113800	-2.63384300
H	3.11016000	-0.45601400	-2.31343500
H	2.32533800	0.25357400	-3.73649200
H	2.71776900	1.27993600	-2.34106900
H	0.06662900	1.41387700	1.61320400
H	1.01874600	4.44953300	-0.00014100
H	4.69822300	-0.57783500	1.32481800
H	0.00563500	-4.50738100	-0.47692400
H	-3.61943500	0.51138400	-1.99234100

^{4}**BC** ub3lyp/def2tzvp, e. e. = -2674.471301 a.u.

C	0.80690400	2.98363400	0.37027900
N	1.23114000	1.67352200	0.15206200
H	1.70185400	4.60197900	1.61541700
C	2.25931700	1.36907400	1.04050600
C	2.63763400	2.60828600	1.66142700
H	3.41680300	2.69418700	2.40755500
C	1.76417400	3.58344200	1.25542500
C	2.68144200	0.09285400	1.40436700
N	0.78899100	-1.36288700	0.69507500
C	0.57780900	-2.72328800	0.68835600
C	1.75146600	-3.38864300	1.20899500
H	1.85890600	-4.46158600	1.30879900
C	2.64631100	-2.41685500	1.54768800
H	3.63568000	-2.53705200	1.97169700
C	2.04629400	-1.14567400	1.20509600
C	-0.43668100	3.49435000	-0.00427700
H	-0.54071000	4.57157600	0.09604400
H	-4.81575400	2.68173100	-1.26569600
C	-3.78776500	2.54512200	-0.95385700
C	-3.15857000	1.26211400	-0.71898700
H	-2.95028000	4.57546900	-0.78465400
N	-1.85348500	1.45855700	-0.33966900
C	-1.62504900	2.81297700	-0.33989600
C	-2.84741100	3.49993000	-0.70970000
C	-0.58392000	-3.38353900	0.27214300
H	-0.55676600	-4.46889300	0.31364500
H	-4.88552100	-2.65088700	-1.32196700
C	-3.87667500	-2.51897200	-0.95043700
C	-2.96781100	-3.48455800	-0.62629300
H	-3.08860900	-4.55948900	-0.68205100
C	-1.76905500	-2.79618500	-0.18376700
N	-1.96755100	-1.45027500	-0.24843300
C	-3.23229500	-1.24095800	-0.70745600
C	-3.79322800	0.02769400	-0.89518500
H	-4.82562400	0.06377300	-1.23182200
C	0.70297300	0.86050600	-0.91051600
C	1.58173600	0.02519200	-1.77969700
O	1.11851800	-0.67032800	-2.66638400
O	2.90471300	0.15205400	-1.55618500
C	3.76914700	-0.65322800	-2.39302200
H	3.59402900	-0.38890800	-3.44065600
H	3.50094300	-1.70665400	-2.26365600
H	3.59482600	0.05767500	1.99229700
Fe	-0.62782200	0.01427500	0.37466600
C	5.20002800	-0.37906700	-1.97444300
H	5.36330500	-0.65227100	-0.92657000
H	5.88359600	-0.97103000	-2.59387700
H	5.45299300	0.67933900	-2.10032400
H	0.10960700	1.47844800	-1.57499800
O	-1.03480100	0.32077200	2.13230800
C	-1.45905500	-0.60670100	3.08283800
H	-1.73704300	-0.08232700	4.01258600
H	-2.34461300	-1.17795300	2.75428100
H	-0.67168700	-1.33571700	3.34395200

^{6}**BC** ub3lyp/def2tzvp, e. e. = -2674.463879 a.u.

N	0.43976400	-2.15438400	0.30722500
Fe	-0.40400600	0.05625700	-0.60550200
C	0.42131800	-0.98153200	1.12602400
C	-1.91322400	-2.87262100	0.70406200
C	-3.16274700	1.83558600	0.30108400
C	-3.23360800	0.45915100	0.57257300
C	-4.38292600	-0.25105100	1.10182000
C	-4.02068200	-1.56207300	1.21522500
C	-2.64198900	-1.66729600	0.77146700
N	-2.20944700	-0.42338900	0.38509600
H	-5.33396600	0.20129900	1.35477700
H	-4.62113300	-2.38854400	1.57560600
C	-0.05442400	-4.28122800	-0.30308800
C	1.48633400	-2.60960900	-0.49025900
C	1.18968200	-3.98216500	-0.79590400
H	-0.61827800	-5.19244900	-0.45429700
H	1.81823300	-4.61065300	-1.41320300
C	-1.99952100	3.98463700	-0.38669100
C	-0.02023300	3.02913400	-0.94074300
C	-0.74581100	4.27644100	-0.84832800
H	-2.80965700	4.67494500	-0.18631400
H	-0.34030300	5.24991900	-1.09505300

C	2.50878000	-1.84384000	-1.06519000
C	1.30882100	2.87479500	-1.35807000
C	3.42000800	1.55892600	-1.89457300
C	2.59217600	-0.45650300	-1.27052200
C	3.74197000	0.23460000	-1.82276500
H	4.03877300	2.37692400	-2.24244000
H	4.67660700	-0.23856700	-2.09777300
C	2.05770300	1.68798100	-1.41272400
C	-0.58258100	-3.10474600	0.32670800
C	-2.06305400	2.55355500	-0.18857200
N	-0.85164900	2.00972200	-0.54260900
N	1.59684900	0.45667000	-1.04145800
C	1.64262900	-0.57053700	1.82490800
O	2.76925200	-1.02929500	1.72123900
O	1.34395300	0.45693000	2.66973000
C	2.44274000	0.96882800	3.44794000
H	3.22285800	1.33383900	2.77106400
H	2.87396300	0.15514900	4.04183600
C	1.90303500	2.07885900	4.32964500
H	2.71388300	2.49736200	4.93710500
H	1.47326300	2.88607300	3.72666100
H	1.12701100	1.70318300	5.00565400
O	-0.77318800	-0.93725100	-2.12397800
C	-1.56642200	-0.52809500	-3.19955000
H	-1.34547300	0.50410000	-3.52448800
H	-1.40031100	-1.18841400	-4.06618600
H	-2.64243900	-0.57361400	-2.95453700
H	-0.42959300	-0.98259400	1.79209600
H	-4.05589100	2.41560600	0.51630700
H	1.82042700	3.78120000	-1.66954200
H	3.32252100	-2.42474000	-1.49197800
H	-2.50293200	-3.77327400	0.85718800

[2]**TS4** ub3lyp/def2tzvp, e. e. = -2753.059195 a.u.
im. frequency -126.29

C	-0.78641300	2.82607700	-0.65893800
N	-0.94038700	1.46738300	-0.82169400
H	-2.14569200	4.57595300	-1.00474600
C	-2.21770100	1.27257900	-1.29315000
C	-2.88589000	2.54320400	-1.43452200
H	-3.89903900	2.66916800	-1.79591100
C	-2.00442000	3.50264400	-1.03386600
C	-2.78041400	0.03600400	-1.58214500
N	-0.85888200	-1.37434700	-0.96820300
C	-0.64259000	-2.72844300	-0.95283800
C	-1.82906200	-3.42333100	-1.39156500
H	-1.91802700	-4.49979900	-1.46917300
C	-2.75821900	-2.47186700	-1.68680600
H	-3.76752100	-2.60580700	-2.05588200
C	-2.14475500	-1.19259900	-1.41778200
C	0.37798400	3.46073100	-0.24368900
H	0.35161700	4.54387800	-0.17127800
H	4.82319900	2.73774100	0.77614600
C	3.77735300	2.59675500	0.53289700
C	3.15194900	1.31481500	0.29918600
H	2.89220100	4.61595900	0.46749900
N	1.82225800	1.48571500	0.01431200
C	1.59001400	2.83496100	0.04290800
C	2.80720600	3.53981600	0.37977800
C	0.55014700	-3.35484600	-0.60947500
H	0.57051900	-4.43954500	-0.65364200
H	4.96260400	-2.52721600	0.46195900
C	3.90861800	-2.41579100	0.23924000
C	2.98603300	-3.38454500	-0.00808800
H	3.12489000	-4.45816600	-0.03674500
C	1.73079500	-2.70913000	-0.25920500
N	1.89276700	-1.35547400	-0.13892900
C	3.21411700	-1.14929000	0.15327800
C	3.80962000	0.09103100	0.36038000
H	4.87078900	0.10431800	0.59031800
C	-0.32699300	0.23078900	1.24154500
C	-1.47693900	-0.56688100	1.78789800
O	-1.41403900	-1.71392900	2.18327500
O	-2.60352600	0.17119500	1.79338400
C	-3.82165100	-0.51384200	2.18761500
H	-3.67945100	-0.92600700	3.19140600
H	-3.98985200	-1.34845600	1.49990200
H	-3.80026900	0.02410800	-1.95453100
Fe	0.49464200	0.04548200	-0.49354400
C	-4.94893100	0.49768200	2.14433600
H	-5.07064800	0.90927700	1.13707600
H	-5.88807000	0.01343100	2.43517300
H	-4.76205200	1.32628300	2.83584700
H	-0.28639700	1.22889800	1.67503300
O	1.12735100	0.09613700	-2.20718400
C	0.39474300	0.43483600	-3.34386300
H	1.02570300	0.26956700	-4.23224300
H	-0.51195700	-0.18057700	-3.46296100
H	0.08391800	1.49273600	-3.35070500
C	0.62307100	0.22419800	3.95353200
H	-0.24939200	-0.18112700	4.46023100
H	0.87916400	1.25797200	4.17634300
C	1.34989600	-0.51371700	3.09896500
H	2.23784200	-0.11310300	2.62247200
H	1.11122800	-1.55322500	2.90258400

[4]**TS4** ub3lyp/def2tzvp, e. e. = -2753.036780 a.u.
im. frequency -465.29

N	-1.35772100	0.70507900	-0.91322400
Fe	0.66934000	0.02024100	-0.50400700
C	-0.72631400	0.23778300	1.11014300
C	-0.89904000	3.08932800	-0.68747400
C	3.51138300	1.48140400	0.74357100
C	2.42513500	2.29668000	0.40465800
C	2.43502700	3.73646500	0.51954900
C	1.20514300	4.18064600	0.13196600
C	0.42423900	3.01894300	-0.22566500
N	1.19414900	1.88819400	-0.05500600
H	3.28220900	4.32067600	0.85722000
H	0.84491800	5.20104600	0.08734700
C	-2.98604700	2.08511200	-1.74385900
C	-2.31324200	-0.06700800	-1.52726500
C	-3.35112100	0.79620400	-2.03544800
H	-3.50741100	2.99694400	-2.00902300
H	-4.22424300	0.46407700	-2.58398000
C	4.69098100	-0.75761400	1.01526100
C	2.91556300	-1.98209700	0.34438200
C	4.29293900	-2.04457700	0.80037000
H	5.65866000	-0.40400000	1.35003500
H	4.87039900	-2.95263200	0.92479000

C	-2.21224700	-1.45052100	-1.67310300
C	2.11869300	-3.06022100	-0.04907900
C	0.07078600	-4.14894400	-0.99830300
C	-1.12913600	-2.26109800	-1.30864700
C	-1.12689900	-3.69865600	-1.46391800
H	0.43162200	-5.16888600	-0.95041100
H	-1.94844900	-4.27494500	-1.87107900
C	0.82162000	-2.99085900	-0.56569200
C	-1.71775100	2.02302500	-1.06072200
C	3.55438600	0.08624900	0.69155100
N	2.50088900	-0.68757200	0.30922300
N	0.06670400	-1.86069100	-0.76306000
C	-1.92088600	-0.61459800	1.49250200
O	-1.88757200	-1.80385300	1.73945300
O	-3.05667000	0.11731100	1.56004700
C	-4.26575700	-0.60922200	1.88346000
H	-4.14170500	-1.08276400	2.86334800
H	-4.40724900	-1.40596200	1.14605900
C	-5.41267500	0.38168200	1.87458700
H	-6.34961300	-0.13517500	2.11193300
H	-5.25915400	1.17093000	2.61865400
H	-5.51730500	0.85095700	0.89068100
O	1.27811400	0.14652400	-2.21938600
C	1.05648600	1.13852300	-3.17260800
H	1.49924200	2.10694200	-2.88372100
H	1.52447100	0.83340500	-4.12308900
H	-0.01465300	1.30661400	-3.37410200
C	0.56875700	-0.21731500	2.88355700
H	1.42915200	0.33537500	2.52704300
H	0.57676400	-1.28858500	2.71651100
C	-0.34413900	0.35844300	3.70223700
H	-0.90153600	1.27943000	1.36156700
H	-0.30922100	1.41959900	3.93710100
H	-1.15523300	-0.21311200	4.14506100
H	-1.30842300	4.08861500	-0.80481800
H	4.40828800	1.99425300	1.07929400
H	2.55333200	-4.05298700	0.02475200
H	-3.05212000	-1.95756200	-2.13949000

[6]**TS4** ub3lyp/def2tzvp, e. e. = -2753.046289 a.u.
im. frequency -400.86

N	1.26872700	-0.63392200	-0.92673400
Fe	-0.73888800	0.03899800	-0.57990800
C	0.78452100	-0.26140300	1.09995700
C	0.83821700	-3.06473900	-0.79197400
C	-3.49406700	-1.51365000	0.79761800
C	-2.44338500	-2.36761900	0.42958200
C	-2.45442700	-3.81645700	0.51614700
C	-1.23796300	-4.25001200	0.07376500
C	-0.47234300	-3.06880200	-0.28121900
N	-1.23512100	-1.95803000	-0.05468900
H	-3.28689700	-4.41355500	0.86811300
H	-0.88193700	-5.27011100	-0.00374300
C	2.87894500	-1.98296800	-1.83552100
C	2.22364800	0.16834400	-1.52996800
C	3.24535800	-0.68318600	-2.07827900
H	3.40085000	-2.88248600	-2.13862000
H	4.11739900	-0.33419200	-2.61785600
C	-4.62238800	0.72705000	1.15769600
C	-2.88752900	1.98886600	0.43575600
C	-4.23542500	2.02239400	0.96485700
H	-5.56895700	0.36460400	1.53949100
H	-4.80504000	2.92320600	1.15791500
C	2.16230900	1.56304200	-1.63130200
C	-2.12084500	3.10902600	0.08115600
C	-0.06685200	4.29650400	-0.82706700
C	1.11404600	2.41167800	-1.24026000
C	1.12805400	3.86019900	-1.31962300
H	-0.41257300	5.31779800	-0.72439000
H	1.95270800	4.45514800	-1.69242800
C	-0.82637100	3.11742300	-0.45407800
C	1.62190000	-1.95748500	-1.13725900
C	-3.51416000	-0.11229200	0.75081300
N	-2.48325600	0.68585500	0.32729300
N	-0.08070200	2.00519500	-0.72050800
C	2.03380300	0.51019600	1.48223900
O	2.06256900	1.68744700	1.78517300
O	3.14503300	-0.26623100	1.46738000
C	4.39078900	0.39231600	1.79253500
H	4.30828100	0.82972100	2.79341700
H	4.55559100	1.21250600	1.08574700
C	5.49312600	-0.64653500	1.72093700
H	6.45567500	-0.18319800	1.96618800
H	5.31418400	-1.46067700	2.43173600
H	5.56412000	-1.07610500	0.71596700
O	-1.30455000	-0.15409500	-2.33243600
C	-1.19482600	-1.06423400	-3.37573200
H	-1.60558500	-2.05151400	-3.10399400
H	-1.75196100	-0.70542900	-4.25709700
H	-0.14749000	-1.21372400	-3.68906800
C	-0.44712900	0.16945800	3.02769000
H	-1.33468600	-0.33897100	2.66928900
H	-0.41335100	1.24397300	2.88487600
C	0.47803700	-0.47167800	3.77383700
H	0.92120400	-1.31766000	1.32593600
H	0.41303900	-1.53939600	3.97012800
H	1.32892300	0.05150100	4.20203800
H	1.28547500	-4.03842200	-0.97224100
H	-4.39630500	-1.99283500	1.16784900
H	-2.58796700	4.07831300	0.23144600
H	3.02418600	2.04428400	-2.08536200

[6]**FP-2** ub3lyp/def2tzvp, e. e. = -5168.831662 a.u.

N	3.71515700	-2.08402200	0.64905900
Fe	4.37930400	-0.10333300	0.42637000
C	4.75068800	-3.05059100	-1.39142300
C	5.07617500	1.71155100	-2.49705500
C	5.16354900	0.30709200	-2.56087900
C	5.75157500	-0.42285600	-3.65900900
C	5.68078200	-1.74599600	-3.34441400
C	5.03698500	-1.84149800	-2.05543900
N	4.74341600	-0.57754700	-1.59330800
H	6.17722700	0.02072900	-4.54718800
H	6.03596900	-2.58091800	-3.93028500
C	3.67617200	-4.38126800	0.46222100
C	3.05786800	-2.62172100	1.73434700
C	3.03332800	-4.06067100	1.61918500
H	3.83158700	-5.36661500	0.04789200
H	2.57897400	-4.73573000	2.32996400
C	4.49545000	3.87175500	-1.32452100

C	3.81624100	2.94049900	0.61484400
C	4.03706800	4.18660200	-0.08176900
H	4.74644900	4.55224700	-2.12488700
H	3.84580100	5.17028500	0.32311700
C	2.47809700	-1.90029600	2.79766500
C	3.29073000	2.84482900	1.91781000
C	2.22320800	1.55858800	3.80380600
C	2.51780700	-0.50024700	2.92182000
C	1.95363200	0.24327900	4.02659200
H	1.95417300	2.40332600	4.42203400
H	1.42457300	-0.18952900	4.86321600
C	2.92335100	1.64037000	2.54234100
C	4.10136400	-3.14298600	-0.14714800
C	4.59113100	2.43061400	-1.39071900
N	4.15290400	1.88790900	-0.20309700
N	3.09996900	0.37325500	2.02934100
O	6.03443200	-0.12057400	1.18116400
C	6.61044900	-0.70178300	2.31455700
H	6.48135200	-1.79537300	2.31994500
H	7.68977100	-0.48856000	2.33198700
H	6.17119900	-0.30024900	3.24141900
C	1.77143900	-2.68055900	3.87227000
C	2.38777500	-2.86454200	5.11623700
C	0.48406900	-3.23938200	3.65905500
C	1.76996400	-3.58700200	6.13700600
H	3.37369500	-2.43639100	5.27494800
C	-0.13728700	-3.96554200	4.68850900
C	0.50787100	-4.13621700	5.91229800
H	2.27180400	-3.72003800	7.09118600
H	-1.11626700	-4.38719500	4.51166400
H	0.01019300	-4.70190600	6.69560600
C	5.11928800	-4.33176100	-2.07683500
C	4.46844900	-4.72789600	-3.25669400
C	6.11750500	-5.16584200	-1.54746300
C	4.80666700	-5.92454200	-3.89044000
H	3.68719600	-4.09721200	-3.67151400
C	6.45751000	-6.36124300	-2.18353000
H	6.63203600	-4.87003300	-0.63740000
C	5.80310700	-6.74441800	-3.35626000
H	4.28673700	-6.21709200	-4.79884800
H	7.23628400	-6.99174100	-1.76267100
H	6.06648000	-7.67587000	-3.85015100
C	5.54749800	2.49470200	-3.68504500
C	6.70528100	3.28602000	-3.60733000
C	4.83934700	2.45595200	-4.89701100
C	7.14112700	4.02018800	-4.71166400
H	7.26689400	3.31717400	-2.67773600
C	5.27427400	3.19272400	-6.00009700
H	3.93827600	1.85310200	-4.96948300
C	6.42652800	3.97685000	-5.91088900
H	8.04215000	4.62301900	-4.63458000
H	4.70977400	3.15544000	-6.92810000
H	6.76505000	4.55020400	-6.76974500
C	3.12939000	4.12167600	2.69913700
C	1.94142600	4.89256600	2.66394500
C	4.20098900	4.56418400	3.48442900
C	1.86185300	6.07867100	3.41439300
C	4.12101400	5.74041700	4.22927000
H	5.10934300	3.96808600	3.50371900
C	2.94597000	6.49070300	4.18716200
H	0.94849300	6.65455900	3.38606900
H	4.96494000	6.06286300	4.83233600
H	2.86355200	7.41064500	4.76009200
N	0.88496400	4.43155400	1.85462600
N	-0.12311100	-3.03409100	2.40438000
C	-0.39399100	4.93142600	1.73138100
O	-0.81591700	5.89108700	2.37376500
C	-1.23994000	-3.63779100	1.86798200
O	-1.96401600	-4.40728400	2.49672400
C	-1.53055200	-3.29058900	0.43227300
C	-2.82362800	-3.56117200	-0.03627900
C	-0.57563800	-2.77036400	-0.45613000
C	-3.18602500	-3.30771300	-1.36219700
H	-3.54264300	-3.98052000	0.66050200
C	-0.92799300	-2.51009200	-1.77891100
H	0.45115600	-2.59478200	-0.14849100
C	-2.22308400	-2.77118600	-2.22583700
H	-0.18606500	-2.11060300	-2.46504300
H	-2.48907200	-2.56366600	-3.25927300
C	-1.27025100	4.19839800	0.75552900
C	-2.65178400	4.28036500	0.96196500
C	-0.78285800	3.47049200	-0.34152800
C	-3.56092800	3.63281400	0.11905400
H	-3.00852400	4.86237300	1.80700000
C	-1.68090600	2.83195200	-1.19459000
H	0.28147500	3.42807000	-0.55823400
C	-3.05708100	2.90538500	-0.96500400
H	-1.30796500	2.27166200	-2.04762400
H	-3.73777400	2.38514500	-1.63098500
H	0.34828100	-2.36223300	1.81489200
H	1.07264600	3.56254200	1.37172100
C	-5.04951700	3.77868700	0.42236300
H	-5.32487300	4.83200600	0.28121500
H	-5.18958000	3.58250400	1.49294100
C	-6.02029800	2.93386000	-0.37548600
C	-6.05612100	1.51437200	-0.21468000
C	-6.90560400	3.51712800	-1.25563400
C	-6.93747700	0.71324500	-0.92813800
C	-7.84867000	2.75285300	-1.99006200
H	-6.89285700	4.59648800	-1.39289500
C	-7.86934500	1.32834700	-1.82956900
C	-8.81912100	0.58114100	-2.58311500
C	-9.69710300	1.20728000	-3.44185900
H	-8.85055000	-0.49752800	-2.46986800
H	-10.41589700	0.61628000	-4.00341700
C	-9.67345500	2.61411000	-3.59701900
C	-8.76672200	3.36668700	-2.88491400
H	-10.37064100	3.09546300	-4.27754600
H	-8.73794300	4.44820400	-2.99660500
C	-6.87774900	-0.77496400	-0.75979600
C	-7.81532900	-1.46652600	0.07855300
C	-5.87568200	-1.49596000	-1.39274500
C	-7.68439500	-2.88329300	0.25053100
C	-5.72012000	-2.90363500	-1.20647800
C	-6.62040000	-3.56246500	-0.39784600
H	-6.52120000	-4.63535000	-0.24639500
C	-4.57934000	-3.64975300	-1.87507100
H	-4.75422600	-4.72361000	-1.73444500
H	-4.59964400	-3.47591500	-2.95681500
C	-8.87558900	-0.80128700	0.75719700

C	-9.75909600	-1.49921800	1.55254700
H	-8.98441500	0.27201500	0.64012800
H	-10.56208400	-0.97043700	2.05928300
C	-8.61200400	-3.57256600	1.07777500
C	-9.62974300	-2.89924000	1.71548800
H	-8.49919800	-4.64731500	1.20077800
H	-10.33187400	-3.43787300	2.34614000
O	-4.95499100	-0.84855300	-2.19099700
O	-5.12703200	0.94923900	0.63012700
C	-5.32903100	-0.71280700	-3.56377100
H	-5.49238000	-1.68864900	-4.04106800
H	-6.23682600	-0.10958700	-3.67660200
H	-4.49727700	-0.20649900	-4.06074400
C	-5.56327800	0.71415200	1.97159900
H	-5.92040700	1.63591600	2.45046900
H	-6.35893100	-0.03760100	2.00987000
H	-4.69033900	0.34389700	2.51460100

^{6}FP-2-EDA ub3lyp/def2tzvp, e. e. = -5584.991732 a.u.

N	-3.34313000	2.28615500	1.22993300
Fe	-4.29761200	0.42949000	0.92062800
C	-2.27679200	-2.64434300	-3.37590800
C	-4.72394600	3.67483400	-0.29499700
C	-6.01294200	-0.77714600	-1.88977300
C	-5.90636700	0.61977100	-1.73923500
C	-6.65102100	1.58214500	-2.51733200
C	-6.30465900	2.81865200	-2.06440500
C	-5.33712900	2.62848900	-1.01017200
N	-5.11727200	1.28075400	-0.82487500
H	-7.35751100	1.34321300	-3.29857300
H	-6.67530000	3.77305000	-2.40878600
C	-3.06827000	4.56856100	1.38620300
C	-2.38608800	2.56688600	2.17870900
C	-2.21619400	3.99766700	2.28394400
H	-3.19935600	5.62118700	1.18176100
H	-1.53315400	4.49891500	2.95514700
C	-5.42561000	-3.15275700	-1.26114300
C	-4.11961600	-2.63968300	0.50754300
C	-4.67637200	-3.71876000	-0.27523100
H	-5.96610500	-3.65994400	-2.04681900
H	-4.49123700	-4.76995900	-0.10918200
C	-1.66389900	1.62089700	2.93317900
C	-3.24595000	-2.81998700	1.59826300
C	-1.58011200	-1.97101600	3.29307700
C	-1.83268700	0.22766200	2.83619200
C	-1.11054700	-0.73965400	3.63104200
H	-1.25740200	-2.92404000	3.68658500
H	-0.34450700	-0.50567800	4.35575200
C	-2.56873000	-1.77821200	2.25705500
C	-3.77520600	3.49480900	0.72777000
C	-5.35120800	-1.72011200	-1.08278600
N	-4.53360100	-1.43609200	-0.01186200
N	-2.71081800	-0.42942000	1.99973700
C	-1.45389500	-2.46868700	-2.18790500
O	-1.25788300	-3.34718000	-1.35562500
O	-0.95024000	-1.22612900	-2.16056200
C	0.01526100	-0.88130600	-1.12647100
H	-0.36301800	0.03999100	-0.67623400
O	-5.67774300	0.48152800	2.10610100
C	-5.81541300	0.80179300	3.46003300
H	-5.39474800	1.79360600	3.68804600
H	-6.88165300	0.81576800	3.73157000
H	-5.31341600	0.06058700	4.10184700
H	-2.44957900	-1.88596300	-4.12643100
C	-0.65992200	2.12890400	3.93105200
C	-0.96914900	2.07519400	5.29643300
C	0.60488100	2.63768000	3.53785800
C	-0.06587500	2.50286400	6.26866700
H	-1.94027200	1.68652500	5.59049400
C	1.51670400	3.06205400	4.51958400
C	1.17753800	2.99209300	5.86935100
H	-0.33095500	2.45065700	7.32074500
H	2.48385100	3.43123700	4.21046100
H	1.89845200	3.32627800	6.61089800
C	-5.09001600	5.08216500	-0.65972500
C	-4.71138900	5.62996100	-1.89629800
C	-5.81646900	5.88429500	0.23555400
C	-5.04921900	6.94333100	-2.22778000
H	-4.14342900	5.02411000	-2.59711200
C	-6.15624600	7.19682400	-0.09738900
H	-6.12116900	5.47114700	1.19332400
C	-5.77296600	7.73068600	-1.32962800
H	-4.74237100	7.35139800	-3.18736800
H	-6.72310300	7.80055200	0.60650400
H	-6.03629100	8.75283000	-1.58830200
C	-6.89353600	-1.29258600	-2.98727000
C	-8.07325600	-1.99656600	-2.69479700
C	-6.55604100	-1.07940900	-4.33415400
C	-8.89244300	-2.47159600	-3.72048200
H	-8.35146600	-2.16078600	-1.65753700
C	-7.37435000	-1.55618600	-5.35952200
H	-5.64525800	-0.53749800	-4.57426500
C	-8.54530700	-2.25410200	-5.05567400
H	-9.80447500	-3.00928100	-3.47483200
H	-7.09475700	-1.38432500	-6.39577100
H	-9.18341700	-2.62452300	-5.85355500
C	-3.09714500	-4.21559200	2.13178500
C	-1.91083000	-4.97499800	2.05459200
C	-4.21432700	-4.78644700	2.77003500
C	-1.86465200	-6.25067900	2.63949200
C	-4.16210400	-6.05221200	3.34632800
H	-5.13046400	-4.20541600	2.82635700
C	-2.97407200	-6.78397600	3.28576000
H	-0.94514800	-6.82069500	2.57443500
H	-5.03847000	-6.45998300	3.84236200
H	-2.91315200	-7.77370900	3.73011500
H	0.02479600	-1.66735100	-0.37186000
N	-0.76223000	-4.49769500	1.36920500
N	0.88577900	2.72690700	2.15929700
C	0.50248200	-4.54753000	1.91644500
O	0.70176500	-4.81010600	3.10201100
C	2.05634600	3.09390800	1.53193800
O	3.11623300	3.28988300	2.12577400
C	1.96091100	3.24361400	0.03809000
C	3.15660700	3.14522400	-0.69028400
C	0.76477800	3.52779600	-0.63465400
C	3.17504400	3.29006500	-2.07786600
H	4.07200600	2.94966900	-0.14430500
C	0.77306100	3.68235900	-2.02238700

H	-0.16449300	3.67531900	-0.09226000
C	1.96330800	3.55642100	-2.73608000
H	-0.15222900	3.91047100	-2.54463300
H	1.95725400	3.67585000	-3.81767400
C	1.64623100	-4.28286500	0.97982400
C	2.83532000	-3.78551100	1.52818500
C	1.59615500	-4.59878500	-0.38585000
C	3.97451300	-3.58115200	0.74282700
H	2.85962100	-3.56921200	2.59245900
C	2.72969300	-4.40933100	-1.17533300
H	0.69136300	-5.00444100	-0.82477600
C	3.90629200	-3.90193700	-0.61937600
H	2.70072200	-4.66682200	-2.23109700
H	4.77812000	-3.75882100	-1.25022500
H	0.12483700	2.45068200	1.55322000
H	-0.89506200	-4.03995100	0.46854600
C	1.38059700	-0.68041800	-1.75701300
H	1.34911400	0.10100500	-2.52298100
H	2.10564700	-0.37990800	-0.99354400
H	1.73871400	-1.60968500	-2.21198300
C	5.25855600	-3.09332000	1.41308900
H	5.72441800	-3.95137500	1.91688000
H	4.98282400	-2.38881500	2.20320700
C	6.28775200	-2.47153500	0.49301000
C	6.21449800	-1.08715800	0.14647300
C	7.29798000	-3.23501800	-0.05171300
C	7.11375900	-0.50238500	-0.73840200
C	8.24469100	-2.69337900	-0.95816100
H	7.37388700	-4.28833000	0.21097200
C	8.14656700	-1.31074100	-1.32416500
C	9.08293900	-0.80028100	-2.26847800
C	10.06627200	-1.60483800	-2.80282600
H	9.01725100	0.24035600	-2.56705400
H	10.76983500	-1.19115700	-3.52059900
C	10.16964300	-2.96515000	-2.42500100
C	9.27417200	-3.49452100	-1.52344700
H	10.95197800	-3.58760000	-2.85090600
H	9.33704100	-4.54032000	-1.23122900
C	6.97804000	0.94965700	-1.08088900
C	7.92163300	1.91848300	-0.59398000
C	5.90325400	1.38203700	-1.84650400
C	7.74104100	3.30096100	-0.92885600
C	5.69315600	2.76169800	-2.14863900
C	6.61165300	3.68335400	-1.69679800
H	6.46738500	4.73862700	-1.91973500
C	4.45787900	3.20065400	-2.90297500
H	4.65306100	4.18846100	-3.34098500
H	4.27174600	2.52713700	-3.74726400
C	9.03284600	1.56858000	0.22519100
C	9.92063300	2.52541800	0.66799100
H	9.17759800	0.53056800	0.50369900
H	10.76063400	2.23241700	1.29231300
C	8.67765700	4.26191800	-0.46009700
C	9.74844700	3.88616000	0.31889100
H	8.52620000	5.30587300	-0.72479400
H	10.45667700	4.63015300	0.67337400
O	4.95769300	0.48725000	-2.30145000
O	5.18772900	-0.34864900	0.68896400
C	5.29772200	-0.19045800	-3.51229500
H	5.45002300	0.51491000	-4.34143800
H	6.20259600	-0.79747100	-3.39516100
H	4.45321800	-0.84324000	-3.74708100
C	5.52602800	0.41288100	1.85438600
H	5.81806000	-0.23992800	2.68868600
H	6.34174400	1.11515100	1.65126000
H	4.63133200	0.97523700	2.13016900
N	-2.86820600	-3.79750300	-3.53832300
N	-3.37266200	-4.80720800	-3.66840100

^{2}TS2-2 ub3lyp/def2tzvp, e. e. = -5584.944754 a.u.
im. frequency -309.24

N	-3.44476900	2.14686500	0.73354500
Fe	-3.96867000	0.21576100	0.37835400
C	-2.32032200	0.21285800	-0.71552600
C	-4.61964900	3.22214000	-1.17096700
C	-6.03832400	-1.39822600	-1.87873400
C	-5.88226000	-0.00928200	-2.00056200
C	-6.59792200	0.80522600	-2.95497500
C	-6.17073100	2.08366900	-2.78275300
C	-5.20736200	2.06365600	-1.70513000
N	-5.03944000	0.77733300	-1.25314100
H	-7.33651800	0.44178400	-3.65417300
H	-6.49755800	2.96826600	-3.30942000
C	-3.28159200	4.44238800	0.57128800
C	-2.70159400	2.63321500	1.78531900
C	-2.61446500	4.07179500	1.69776500
H	-3.41758300	5.44149400	0.18414300
H	-2.10686400	4.71080300	2.40606700
C	-5.41244200	-3.61590100	-0.87675100
C	-4.00275300	-2.80578700	0.68323300
C	-4.59024100	-4.00699100	0.13043400
H	-6.01974400	-4.23785700	-1.51790700
H	-4.39983600	-5.01098400	0.48079000
C	-2.09190200	1.86713400	2.79222600
C	-3.15034200	-2.80164600	1.79863300
C	-1.79407700	-1.63919700	3.55921100
C	-2.16167800	0.46676200	2.84051000
C	-1.50292200	-0.34195100	3.84095200
H	-1.48048800	-2.52215100	4.09731600
H	-0.89976400	0.03999600	4.65153800
C	-2.62975400	-1.63927100	2.38140900
C	-3.81889200	3.23875700	-0.01976500
C	-5.32868900	-2.17385900	-0.95195200
N	-4.44221300	-1.70782800	-0.01088600
N	-2.83687500	-0.34636000	1.95570300
C	-1.05166300	-0.50160200	-0.33347000
O	-0.85699600	-1.69332700	-0.48443400
O	-0.20174200	0.36718500	0.21726300
C	1.07096500	-0.16097000	0.72450800
H	1.33027000	0.52244900	1.53430600
O	-5.49729300	0.32933700	1.36291500
C	-5.62614400	0.95402300	2.60275400
H	-5.41104300	2.03448300	2.56307800
H	-6.66580900	0.83695600	2.94892500
H	-4.97128900	0.51087100	3.37136700
H	-2.13759500	1.11623000	-1.29982200
C	-1.37369900	2.57482000	3.90749200
C	-1.97074700	2.59936800	5.17592500
C	-0.11259100	3.20419800	3.73995500
C	-1.35890400	3.21866300	6.26455900

H	-2.93703600	2.11864900	5.29877100
C	0.50537100	3.82367200	4.84020900
C	-0.11773900	3.82829600	6.08636600
H	-1.84681700	3.22285800	7.23505200
H	1.47113800	4.28752500	4.70326300
H	0.37944800	4.31444400	6.92167400
C	-4.90105000	4.52622200	-1.85735600
C	-4.33375900	4.80713000	-3.11071800
C	-5.74299300	5.48585700	-1.27234800
C	-4.59431300	6.01602200	-3.75859200
H	-3.68221300	4.07329800	-3.57778900
C	-6.00538600	6.69422900	-1.92088800
H	-6.19796300	5.27629500	-0.30815200
C	-5.43087700	6.96364600	-3.16511400
H	-4.14143700	6.21705500	-4.72607600
H	-6.66327200	7.42292900	-1.45461200
H	-5.63498700	7.90427800	-3.66971600
C	-7.00769600	-2.09120800	-2.78724700
C	-8.19645100	-2.63621800	-2.27518300
C	-6.75075900	-2.21304700	-4.16255800
C	-9.10341100	-3.28377300	-3.11594600
H	-8.40975700	-2.54289100	-1.21390700
C	-7.65727100	-2.86218800	-5.00285000
H	-5.83099700	-1.80347900	-4.57175200
C	-8.83659500	-3.39913100	-4.48202300
H	-10.02047400	-3.69518700	-2.70228100
H	-7.43869800	-2.95138100	-6.06390300
H	-9.54258300	-3.90403200	-5.13610700
C	-2.90920600	-4.11106600	2.49696300
C	-1.72259000	-4.86725800	2.35747400
C	-3.92881700	-4.60101400	3.32919200
C	-1.60107700	-6.08707800	3.04934900
C	-3.79934900	-5.80301700	4.01958300
H	-4.83560400	-4.01132400	3.43197200
C	-2.62637800	-6.54407700	3.87107400
H	-0.69403700	-6.66463700	2.94555100
H	-4.60160800	-6.15323700	4.66289400
H	-2.50312300	-7.48891300	4.39394900
H	0.89133000	-1.15452200	1.13677100
N	-0.69578600	-4.42450000	1.48921000
N	0.47359500	3.19785600	2.45543300
C	0.64092600	-4.76459800	1.59490700
O	1.10240800	-5.36659600	2.56219700
C	1.67774800	3.73700100	2.04961300
O	2.51093100	4.21026800	2.81915100
C	1.92550600	3.70449100	0.56603300
C	3.26089800	3.70241800	0.13788500
C	0.89447800	3.73622700	-0.38481700
C	3.58627700	3.70255900	-1.22151200
H	4.04241600	3.70604400	0.89105400
C	1.20898500	3.74020900	-1.74317200
H	-0.14583200	3.80193100	-0.07990100
C	2.54089400	3.71257600	-2.15605000
H	0.41316200	3.77599400	-2.48256500
H	2.77476700	3.71540000	-3.21817900
C	1.52875900	-4.39323900	0.44104900
C	2.90342000	-4.31205900	0.70635900
C	1.07482000	-4.23088800	-0.87474200
C	3.83417100	-4.07032700	-0.30596200
H	3.23605900	-4.46578200	1.72845500
C	1.99637200	-3.98837000	-1.89484000
H	0.01985400	-4.31709300	-1.11271400
C	3.36191600	-3.90653600	-1.61591000
H	1.64797300	-3.87336000	-2.91796000
H	4.06580600	-3.71955700	-2.42251400
H	-0.07496000	2.73484200	1.74155300
H	-0.94217100	-3.70537400	0.81571900
C	2.11335100	-0.17237600	-0.37467200
H	2.23294200	0.82146400	-0.81437700
H	3.08015200	-0.48003900	0.03605800
H	1.84161800	-0.88288700	-1.16113800
C	5.32201700	-4.08739800	0.02769200
H	5.69945900	-5.10320900	-0.15142000
H	5.43047700	-3.91509100	1.10364300
C	6.21007000	-3.12813200	-0.74023800
C	6.21759800	-1.73104700	-0.43272300
C	7.04489000	-3.58719600	-1.73550900
C	7.03540100	-0.83345400	-1.10445200
C	7.91106700	-2.72010800	-2.45001000
H	7.05662800	-4.64791600	-1.97802000
C	7.91555200	-1.32321200	-2.13074500
C	8.79908000	-0.47354200	-2.85547500
C	9.62618600	-0.97653200	-3.83688000
H	8.81712400	0.58661100	-2.62693200
H	10.29357500	-0.30753000	-4.37380200
C	9.61583100	-2.35650000	-4.15056100
C	8.77409300	-3.20663800	-3.46921100
H	10.27248400	-2.74046200	-4.92653100
H	8.75753600	-4.26931100	-3.70036800
C	7.01961100	0.62876300	-0.76518600
C	8.02134400	1.17961100	0.10580200
C	6.06054700	1.47176600	-1.31015300
C	8.00273300	2.58208800	0.39735200
C	6.04430700	2.87611400	-1.03735500
C	7.00199400	3.39522500	-0.19387600
H	7.00765800	4.46231100	0.01913400
C	5.02897900	3.78664600	-1.70291200
H	5.37439900	4.81936700	-1.56222000
H	5.03470000	3.61486400	-2.78492300
C	9.04263400	0.38499300	0.69989900
C	9.98723500	0.94588400	1.53259300
H	9.07154100	-0.67904500	0.49063800
H	10.75740700	0.31861400	1.97367800
C	8.98999500	3.12931600	1.26097100
C	9.96378800	2.33169100	1.81853300
H	8.96018600	4.19525100	1.47427900
H	10.71337600	2.76117100	2.47760000
O	5.05052700	0.97432600	-2.10736300
O	5.33424700	-1.29646600	0.53348600
C	5.35255100	0.83052100	-3.49731900
H	5.74808000	1.76035700	-3.92729800
H	6.07271900	0.02519900	-3.67507900
H	4.40891400	0.58146300	-3.99006200
C	5.85970000	-1.16435600	1.85867800
H	6.36256100	-2.08359200	2.18622700
H	6.56215500	-0.32811700	1.93201400
H	5.00270600	-0.97294200	2.50910500
N	-2.62522700	-0.81076500	-2.36607800
N	-3.02675500	-1.47356100	-3.15325200
